# Ethnic sensitivity assessment of fluticasone furoate/vilanterol in East Asian asthma patients from randomized double-blind multicentre Phase IIb/III trials

**DOI:** 10.1186/s12890-015-0159-z

**Published:** 2015-12-24

**Authors:** Annette S. Gross, Caroline Goldfrad, Soichiro Hozawa, Mark H. James, Christine S. Clifton, Yutaro Sugiyama, Loretta Jacques

**Affiliations:** Clinical Pharmacology Modelling & Simulation, GSK R&D, 82 Hughes Ave, Ermington, Sydney, NSW 2115 Australia; Quantitative Sciences Division, GSK, Uxbridge, UK; Hiroshima Allergy and Respiratory Clinic, Hiroshima, Japan; Medical Affairs, GSK K.K., Tokyo, Japan; Medicines Development Respiratory, GSK K.K., Tokyo, Japan; Respiratory Medicines Discovery and Development, GSK, Uxbridge, UK

**Keywords:** β_2_-adrenergic receptor agonists, Asthma, Glucocorticoids, Japan, Treatment outcome

## Abstract

**Background:**

Fluticasone furoate (FF)/vilanterol (VI) is a once daily (OD) inhaled corticosteroid/long-acting β_2_-agonist combination asthma therapy approved in Japan and the EU. FF/VI efficacy and safety data from asthma studies including patients in East Asia were evaluated to assess ethnic sensitivity.

**Methods:**

Randomized, double-blind, multicenter Phase IIb/III trials were assessed. Change from baseline relative to placebo or twice-daily fluticasone propionate 500 μg in trough FEV_1_ was compared between patients from Japan (*N* = 148) and Not-Japan (*N* = 3,066; three studies). Adverse events (AEs), laboratory results, and electrocardiograms were compared between patients from Japan + Korea (*N* = 188) and Not-Japan + Korea (*N* = 3,840; five studies).

**Results:**

For trough FEV_1_, improvements from baseline (least-squares mean difference [95 % confidence interval]) were reported for FF/VI 100/25 μg OD versus placebo at Week 12 (Japan: 0.323 L [0.104–0.542]; Not-Japan: 0.168 L [0.095–0.241]). Improvements from baseline (least-squares mean change [standard error]) were reported with FF/VI 200/25 μg OD at Week 24 (Japan: 0.355 L [0.1152]; Not-Japan: 0.396 L [0.0313]). A greater proportion of patients from Japan + Korea versus Not-Japan + Korea reported AEs in all treatment arms including placebo (FF/VI 100/25 μg: 79 % versus 57 %; FF/VI 200/25 μg: 64 % versus 45 %; placebo: 41 % versus 23 %). There were no notable differences in treatment-related or class-related AEs. No clinically significant changes in electrocardiogram assessments or statistically significant differences in 24 h urinary cortisol excretion were observed between the Japan + Korea and Not-Japan + Korea cohorts.

**Conclusions:**

Good efficacy and an acceptable safety profile were observed for FF/VI 100/25 μg and 200/25 μg OD in East Asian asthma patients; these globally recommended doses are appropriate for asthma patients in Japan.

**Trial registration:**

Clinicaltrials.gov registration numbers: NCT01165138, NCT01134042, NCT01086384, NCT00603278, NCT00603382.

**Electronic supplementary material:**

The online version of this article (doi:10.1186/s12890-015-0159-z) contains supplementary material, which is available to authorized users.

## Background

Asthma represents a considerable disease burden worldwide. The prevalence of asthma in Japan is amongst the highest in Asia [1]. Japanese [2] and global asthma guidelines [3] recommend the use of a low-dose inhaled corticosteroid (ICS) and long-acting β_2_-agonist (LABA) combination as step-up therapy for asthma patients uncontrolled using reliever medication and low-dose ICS alone. The inhaled ICS and LABA combination of fluticasone furoate (FF) and vilanterol (VI) is suitable for once-daily (OD) dosing having 24 h efficacy in asthma patients [4, 5]. FF/VI is delivered via the ELLIPTA® dry powder inhaler (DPI; ELLIPTA® is a trademark of the GSK group of companies).

The Japanese and global asthma guidelines support the use of ICS/LABA combination inhalers [2, 3]; they reduce the number of inhalation procedures, increase patient adherence, and reduce the risk of the LABA being delivered alone [2]. Additionally, OD therapy has been shown to improve treatment adherence in asthma patients [6, 7] and is associated with treatment success and decreased healthcare costs [8]. OD FF/VI 100/25 μg and 200/25 μg are approved in Japan, the USA, and the European Union for the treatment of asthma.

The multiregional global development program of FF/VI included patients from a range of ethnic groups, including East Asian patients from Japan and Korea. Based on the totality of data from the global development program, the 100/25 μg and 200/25 μg strengths of FF/VI were recommended for the treatment of asthma. As responses to pharmacotherapy can vary across ethnic groups [9, 10], the potential ethnic sensitivity of FF/VI efficacy and safety in East Asian patients was assessed, using data from the studies that included asthma patients in Japan and Korea. This analysis also supported the asthma FF/VI submission for regulatory approval in Japan. Consequently, the focus of this assessment is asthma patients in Japan and therefore, efficacy data from patients in Japan have been compared with data from patients in all countries other than Japan. This comparison is important to determine whether the results in the subjects in Japan are comparable with all other subjects studied and, therefore, whether there is a major difference in drug response in patients in Japan relative to all other subjects studied. To provide more reliable conclusions on the safety profile of FF/VI in East Asian asthma patients, safety data from patients in Korea were included to increase the number of patients in the safety assessment. The safety results of asthma patients from Japan and/or Japan + Korea versus Not-Japan and/ or Not-Japan + Korea have therefore been compared.

## Methods

This was a pre-specified efficacy and safety subgroup analysis of all multicenter, randomized, double-blind, parallel-group, multiregional studies that included East Asian asthma patients recruited in Japan (three Phase III studies: efficacy assessment) and Japan and/or Korea (five studies: safety assessment). Inclusion and exclusion criteria are presented in each clinical trial summary (Clinicaltrials.gov; registration numbers: NCT01165138, NCT01134042, NCT01086384, NCT00603278 and NCT00603382) and asthma-related clinical criteria are described in Additional Files 1 and 2. In brief, non-smoking patients with a diagnosis of asthma as defined by the National Institutes of Health [11] aged ≥12 years with a pre-bronchodilator % predicted forced expiratory volume in one second (FEV_1_) of 40–90 %, and FEV_1_ reversibility of ≥12 % and ≥200 mL within 10–40 min following 200–400 μg inhaled salbutamol were included. Permitted baseline asthma therapies are described in Additional Files 1 and 2. All studies included in the analysis were approved by local ethics committees (Additional File 3) and carried out in accordance with the Declaration of Helsinki [12]. All patients provided written informed consent prior to undertaking any study-related procedures.

### Efficacy analysis

Efficacy data are presented from three Phase III studies that included asthma patients in Japan: HZA106827 (Clinicaltrials.gov registration number NCT01165138) [4]; HZA106829 (NCT01134042) [5]; HZA106837 (NCT01086384) [13] (Additional File 1). FF/VI 100/25 μg OD and FF 100 μg OD were investigated in studies HZA106827 (placebo-controlled) and HZA106837. FF/VI 200/25 μg OD, FF 200 μg OD, and fluticasone propionate (FP) 500 μg twice daily (BD) were investigated in study HZA106829. FF/VI and placebo were delivered via the ELLIPTA DPI and FP was delivered via the DISKUS® (DISKUS® is a trademark of the GSK group of companies) DPI. A range of efficacy endpoints were assessed in the individual studies, but only trough FEV_1_ measured at the end of a dosing interval (the primary endpoint in studies HZA106827 and HZA106829 and a secondary endpoint in study HZA106837) was analyzed in the Japan subgroup, as pre-specified in the analysis plan. Efficacy data were analyzed for patients recruited from Japan (Japan cohort), all patients that were not recruited from Japan (Not-Japan cohort), and all patients studied (Overall population). Patients were assigned to these cohorts according to the country in which they were recruited and not according to race. Ease of use of the ELLIPTA inhaler was also assessed by questionnaire in study HZA106827.

Change from baseline in trough FEV_1_ at Week 12 was pooled for each treatment arm from studies HZA106827 and HZA106837. Statistical analyses for treatment comparisons at Week 12 were conducted for FF/VI 100/25 μg versus placebo, FF/VI 100/25 μg versus FF 100 μg, and FF 100 μg versus placebo. In the individual studies included in this analysis, sensitivity analyses (using repeated measures analyses) supported the primary analysis using last observation carried forward (LOCF) data for imputation of missing data. Consequently, LOCF data were used to perform the current statistical analysis using an analysis of covariance (ANCOVA) model. Effects due to geographic region, baseline FEV_1_, gender, age, treatment group, and study were modeled. An interaction term for treatment by geographic region (Japan/Not-Japan) was also included in the model. A similar modeling process was used to analyze the results of the FP 500 μg BD-controlled study HZA106829, in which FF/VI 200/25 μg and FF 200 μg were assessed at Weeks 12 (post-hoc analysis) and 24, using last observation carried forward data.

Withdrawals due to lack of efficacy were summarized for each cohort in the efficacy dataset.

### Safety analysis

Safety data were pooled from five clinical studies: the three studies included in the efficacy assessment and an additional two placebo-controlled FF Phase IIb studies that included East Asian patients in Korea: FFA109685 (NCT00603278) [14]; FFA109687 (NCT00603382) [15] (Additional File 2). These two studies were included in the integrated safety analysis to increase the number of East Asian patients assessed and thereby increase confidence in the results. There are similarities in intrinsic and extrinsic ethnic factors in the populations of Japan and Korea [16–18], supporting the pooling of patient results in these two East Asian populations, which has increased the number of East Asian patients assessed and thereby increased confidence in the conclusions that can be drawn.

All five studies assessed a comprehensive range of safety endpoints, which are presented in the relevant publications; however, for brevity we only assessed key safety data in the Japan subgroup, as pre-specified in the analysis plan. Safety data were compared between treatment arms for patients recruited from Japan and Korea (Japan + Korea cohort), patients recruited from all other countries (Not-Japan + Korea cohort), and all patients studied (Overall population). The safety results are considered for OD placebo, FF/VI 100/25 μg, FF/VI 200/25 μg, FF 100 μg, and FF 200 μg from studies HZA106827, HZA106837, HZA106829, FFA109685, and FFA109687. Results for the doses of FF monotherapy that were not progressed to Phase III for FF/VI from these five studies are not presented. FP 100 μg BD, FP 250 μg BD, and FP 500 μg BD were also included in the analysis of urinary cortisol data.

The incidence of all adverse events (AEs), treatment-related AEs, and serious AEs (SAEs), including exacerbations, was summarized for patients in Japan + Korea, Not-Japan + Korea, and the Overall population of the pooled data set. Electrocardiogram (ECG) results including QT corrected using Frederica’s correction (QTcF) were also assessed in a subset of patients from HZA106827 and HZA106829. AEs related to known ICS or LABA class effects were a focus, including lower respiratory tract infections (bronchitis and pneumonia), cardiovascular effects (heart rate and ECG), and effects on glucose and potassium. An Asthma Composite Endpoint was also derived to assess independently adjudicated asthma-related hospitalizations, intubations, and deaths in studies HZA106837, HZA106827, and HZA106829.

The urinary cortisol excretion over 24 h was measured at baseline and at the end of the treatment period in a subset of patients from studies FFA109685, FFA109687, HZA106827, and HZA106829 (Urinary Cortisol population). For treatment comparisons, the urinary cortisol excretion ratio (end of treatment/baseline) was analyzed using an ANCOVA model of the log-transformed urinary cortisol excretion with effects due to geographic region, log baseline urinary cortisol, gender, age, treatment group, and study. For the analysis by geographic region and for estimation of regional treatment differences, an interaction term for treatment by geographic region (Japan + Korea/Not-Japan + Korea) was also included in the model.

Withdrawals due to AEs were summarized for each cohort in the safety dataset.

## Results

### Efficacy analysis

The intent-to-treat populations in the three efficacy studies comprised 3,214 patients recruited in 11 countries (overall population); including 148 patients (5 %) recruited from Japan (Additional File 1). Eighty-five percent of patients in the Overall population and 70 % of the Japan cohort completed the respective study. A higher proportion of patients recruited from Japan (14 %; *N* = 21/148) than Not-Japan (3 %; *N* = 104/3,066) withdrew due to lack of efficacy. In the Japan cohort, a greater proportion of patients withdraw due to lack of efficacy with placebo (42 %) than with FF/VI (7–9 %). A similar pattern was observed in the Not-Japan group (placebo 13 %, FF/VI 1–3 %).

Demographic and baseline characteristics for the Japan and Not-Japan patients are presented in Table 1. All patients recruited from Japan were of East Asian ancestry and patients from the Not-Japan cohort were principally White/Caucasian (81 %), but included small proportions of patients of a range of geographic ancestries (Additional File 1). Mean baseline pre-bronchodilator FEV_1_ was lower in patients recruited from Japan than Not-Japan, consistent with the literature and the lower average height of the Japan patients [19, 20]. Furthermore, patients recruited from Japan had greater mean % predicted FEV_1_ values than the Not-Japan patients.

**Table 1:** Patient demographics and lung function at screening and baseline (Efficacy population)

	Japan *N* = 148	Not-Japan *N* = 3,066	Overall *N* = 3,214
Demographic characteristics			
Age, years*	47.5 (14.66)	41.9 (16.63)	42.2 (16.59)
Range: min, max	20, 82	12, 84	12, 84
Male/female, %	38/62	36/64	36/64
Weight, kg*	62.5 (13.63)	76.2 (19.29)	75.5 (19.28)
Height, cm*	160.7 (8.05)	165.7 (9.98)	165.5 (9.96)
Screening and baseline lung function characteristics			
Screening			
Pre-bronchodilator FEV_1_, L*	1.87 (0.505)	2.13^†^ (0.619)	2.12^‡^(0.617)
% predicted pre-bronchodilator FEV_1_, %*	72.63 (10.715)	67.39^†^ (11.179)	67.63^‡^ (11.210)
Post-bronchodilator FEV_1_, L *	2.34 (0.638)	2.67^§^ (0.768)	2.65^¶^ (0.766)
Absolute reversibility in FEV_1_, mL*	463.6 (258.51)	537.5^‖^ (306.75)	534.1** (305.05)
Range: min,max	197.0, 2055.0	6.0, 2628.0	6.0, 2628.0
FEV_1_ reversibility, %*	25.3 (14.00)	26.1^‖^ (14.95)	26.1** (14.90)
Range: min, max	12.0–129.9	0.3–125.2	0.3–129.9
Baseline			
Baseline FEV_1_, L*	1.93^††^ (0.548)	2.23^‡‡^ (0.647)	2.22^§§^ (0.646)
% predicted^¶¶^ FEV_1_ at baseline, %*	74.66^††^ (11.127)	70.57^‡‡^ (11.182)	70.75^§§^ (11.210)

*FEV*_*1*_ forced expiratory volume in one second, *min* minimum, *max* maximum

Efficacy population consists of data from studies HZA106827, HZA106829, and HZA106837. *Mean (SD); ^†^ *N* = 3,055; ^‡^ *N* = 3,203; ^§^ *N* = 3,063; ^¶^ *N* = 3,211; ^‖^ *N* = 3,052; **N = 3,200; ^††^ *N* = 147; ^‡‡^ *N* = 3,059; ^§§^ *N* = 3,206; ^¶¶^Study HZA106837 used the prediction equation of Hankinson et al. 1999 [34]; studies HZA106827 and HZA106829 used Hankinson et al. 2010 [35]

**Fig. 1:**
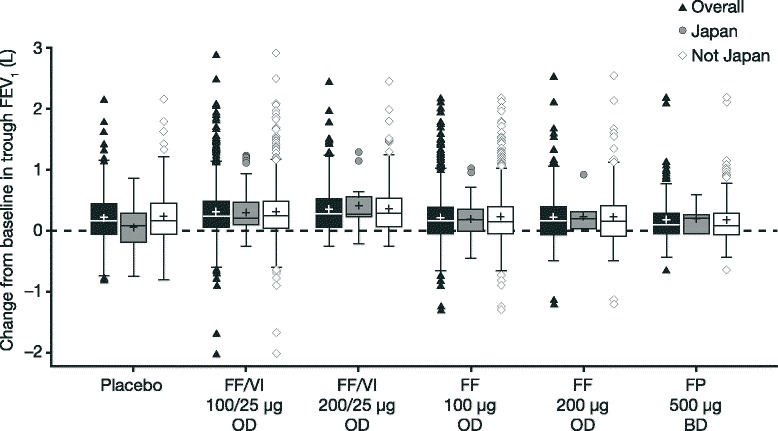
Change from baseline in trough FEV_1_ at Week 12 (Efficacy population). Efficacy population consists of data from studies HZA106827, HZA106829, and HZA106837. Number of patients analyzed in a) Overall population: *N* = 193 placebo, *N* = 1,201 FF/VI 100/25 μg OD, *N* = 187 FF/VI 200/25 μg OD, *N* = 1,203 FF 100 μg OD, *N* = 186 FF 200 μg OD, *N* = 190 FP 500 μg BD; b) Japan cohort: *N* = 18 placebo, *N* = 46 FF/VI 100/25 μg OD, *N* = 13 FF/VI 200/25 μg OD, *N* = 46 FF 100 μg OD, *N* = 11 FF 200 μg OD, *N* = 10 FP 500 μg BD; c) Not-Japan cohort: *N* = 175 placebo, *N* = 1,155 FF/VI 100/25 μg OD, *N* = 174 FF/VI 200/25 μg OD, *N* = 1,157 FF 100 μg OD, *N* = 175 FF 200 μg OD, *N* = 180 FP 500 μg BD. The limits of the box represent the interquartile range (IQR) from the 25th to 75th percentiles, respectively, for the lower and upper limits of the box; error bars represent the minimum and maximum values within 1.5 × IQR below and above the 25th and 75th percentiles, respectively; the horizontal line within the box represents the median; + represents the mean; symbols represent data that are either <1.5 × IQR below the 25th percentile or >1.5 × IQR above the 75th percentile. *BD* twice daily, *FEV*_*1*_ forced expiratory volume in one second, *FF* fluticasone furoate, *FP* fluticasone propionate, *OD* once daily, *VI* vilanterol

At Week 12, increases from baseline in trough FEV_1_ were observed for FF/VI 100/25 μg, FF/VI 200/25 μg, and all active treatments in the Japan and Not-Japan patients (Tables 2 and 3, and Fig. 1). The least-squares mean change from baseline in the placebo group showed a slight decrease in the Japan cohort (−0.022 L) compared with an increase in the Not-Japan cohort (0.133 L). Consequently, greater improvements from baseline for FF/VI 100/25 μg OD versus placebo were observed in patients recruited from Japan than Not-Japan, although the variability was greater in the Japan population due to the smaller number of subjects (Table 2). Adjusted treatment differences for FF/VI 100/25 μg versus placebo, FF 100 μg versus placebo, and FF/VI 100/25 μg versus FF 100 μg were directionally the same in the Japan and Not-Japan populations (Table 2, Additional File 4). Furthermore, there was no evidence of a statistically significant difference in trough FEV_1_ treatment effect between Japan and Not-Japan patients at Week 12 (*P* = 0.403).

**Table 2:** Treatment differences for change from baseline in trough FEV_1_ (L) at Week 12 (Efficacy population)

		Placebo	FF/VI 100/25 μg OD	FF 100 μg OD
Overall	N	203	1,210	1,215
	n^†^	193	1,201	1,203
	LS mean change from baseline (SE)	0.120 (0.0333)	0.301 (0.0116)	0.225 (0.0116)
	Difference *vs* placebo (95 % CI)	–	0.181* (0.111–0.252)	0.105* (0.034–0.175)
	Difference *vs* FF 100 μg (95 % CI)	–	0.077* (0.045–0.108)	–
Japan	N	19	47	46
	n^†^	18	46	46
	LS mean change from baseline (SE)	−0.022 (0.0955)	0.301 (0.0590)	0.194 (0.0591)
	Difference *vs* placebo (95 % CI)	–	0.323 (0.104–0.542)	0.216 (−0.003–0.436)
	Difference *vs* FF 100 μg (95 % CI)	–	0.107 (−0.056–0.270)	–
Not-Japan	N	184	1,163	1,169
	n^†^	175	1,155	1,157
	LS mean change from baseline (SE)	0.133 (0.0345)	0.301 (0.0118)	0.226 (0.0118)
	Difference *vs* placebo (95 % CI)	–	0.168 (0.095–0.241)	0.093 (0.020–0.166)
	Difference *vs* FF 100 μg (95 % CI)	–	0.075 (0.043–0.108)	–

*CI* confidence interval, *FF* fluticasone furoate, *LS* least-squares, *OD* once daily, *SE* standard error, *VI* vilanterol

All values are in L. Data from studies HZA106827 and HZA106837. ^†^Number of patients with analyzable data at Week 12; *P ≤ 0.003; Overall: FF/VI 100/25 μg *vs* placebo, FF100 μg *vs* placebo, FF/VI 100/25 μg *vs* FF100 μg

**Table 3:** Treatment differences and change from baseline in trough FEV1 (HZA106829; Efficacy population)

		FP 500 μg BD		FF/VI 200/25 μg OD		FF 200 μg OD	
		Week 12	Week 24	Week 12	Week 24	Week 12	Week 24
Overall	N	195	195	197	197	194	194
	n^†^	190	190	187	187	186	186
	LS mean change from baseline (SE)	0.178 (0.0291)	0.183 (0.0300)	0.364 (0.0293)	0.394 (0.0302)	0.209 (0.0294)	0.201 (0.0303)
	Difference *vs* FP 500 μg BD (95 % CI)	–	–	0.187 (0.106–0.268)	0.210* (0.127–0.294)	0.032 (−0.050–0.113)	0.018 (−0.066–0.102)
	Difference *vs* FF 200 μg OD (95 % CI)	–	–	0.155 (0.074–0.237)	0.193* (0.108–0.277)	–	–
Japan^‡^	N	11	11	14	14	11	11
	n^†^	10	10	13	13	11	11
	LS mean change from baseline (SE)	0.136(0.1271)	0.110(0.1310)	0.399 (0.1118)	0.355 (0.1152)	0.097 (0.1226)	0.086 (0.1263)
Not-Japan	N	184	184	183	183	183	183
	n^†^	180	180	174	174	175	175
	LS mean change from baseline (SE)	0.183 (0.0300)	0.191 (0.0309)	0.361 (0.0304)	0.396 (0.0313)	0.214 (0.0304)	0.206 (0.0314)
	Difference *vs* FP 500 μg BD (95 % CI)	–	–	0.178 (0.094–0.262)	0.205 (0.119–0.291)	0.031 (−0.053–0.115)	0.015 (−0.072–0.101)
	Difference *vs* FF 200 μg OD (95 % CI)	–	–	0.147 (0.062–0.231)	0.190 (0.103–0.277)	–	–

*BD* twice daily, *CI* confidence interval, *FEV*_*1*_ forced expiratory volume in one second, *FF* fluticasone furoate, *FP* fluticasone propionate, *LS* least-squares, *OD* once daily, *SE* standard error, *VI* vilanterol

All values are in L. Analysis of covariance (ANCOVA) model using last observation carried forward. ^†^Number of patients with analyzable data; ^‡^Differences *vs* FP 500 μg BD and FF 200 μg BD are not presented for the Japan group due the low number of patients in this analysis; *P ≤ 0.001

In the separate FP 500 μg BD-controlled study that assessed FF/VI 200/25 μg and FF 200 μg at Weeks 12 and 24, the least-squares mean changes from baseline in trough FEV_1_ in all treatment arms were consistent in the Japan and Not-Japan patients (Table 3). The results in the Japan subset should be interpreted with caution due to the low number of patients in each treatment arm. Nevertheless in the Japan patients, the least-squares mean changes from baseline in trough FEV_1_ at Week 24 were numerically greater with OD FF/VI 200/25 μg (0.355 L) than with OD FF 200 μg (0.086 L), or BD FP 500 μg (0.110 L). This pattern was consistent with the results in the Not-Japan patients (FF/VI 200/25 μg: 0.396 L; FF 200 μg: 0.206 L; FP 500 μg: 0.191 L).

In the Japanese asthma patients, the ELLIPTA DPI was considered easy or very easy to use by the majority of subjects (77 %), which is consistent with results for the Overall patient population (Additional Files 5 and 6).

### Safety analysis

The safety analysis included 188 patients (5 %) recruited from Japan and Korea (Japan *N* = 148, Korea *N* = 40) included in the five studies (Overall Safety population *N* = 4,028 recruited from 21 countries (Additional Files 1 and 2). Demographic and baseline characteristics of the Japan + Korea patients in the Safety population (data not shown) were consistent with the Efficacy population (Table 1). Studies were completed by 139 (74 %) patients in the Japan + Korea cohort and 3,266 (85 %) patients in the Not-Japan + Korea cohort. The number of patients who withdrew due to an AE with FF/VI 100/25 μg, FF/VI 200/25 μg, and placebo in Japan + Korea was 3 (6 %), 1 (7 %), and 0 patients, respectively, and for Not-Japan + Korea was 14 (1 %), 6 (3 %), and 1 (<1 %), respectively.

Exposure to treatment varied across treatment arms as a consequence of the different durations of the studies integrated (Additional File 7) and should be considered when interpreting the incidence of AEs in each treatment group. For example, in both Japan + Korea and Not-Japan + Korea patients, there was a >5-fold longer mean duration of exposure in the FF/VI 100/25 μg treatment group relative to the placebo group.

On-treatment AEs (Table 4) were reported by a greater proportion of Japan + Korea patients (placebo: 41 %; FF/VI: 64–79 %; FF: 69–73 %) than Not-Japan + Korea patients (placebo: 23 %; FF/VI: 45–57 %; FF: 36–54 %). The most frequently reported on-treatment AEs in all populations were nasopharyngitis, headache, bronchitis, and upper respiratory tract infections (Additional File 8). A greater proportion of Japan + Korea patients reported nasopharyngitis (placebo: 21 %; FF/VI: 49–50 %; FF: 23–29 %) than Not-Japan + Korea patients (placebo: 5 %; FF/VI: 10–13 %; FF: 8–10 %). A lower proportion of Japan + Korea patients reported headache (placebo: 3 %; FF/VI: 0–6 %; FF: 7–9 %) than Not-Japan + Korea patients (placebo: 6 %; FF/VI: 6–17 %; FF: 7–15 %). For the pooled dataset, the incidences of AEs deemed to be treatment related by the investigator, as well as fatal SAEs and non-fatal SAEs, were all low and generally reported for similar proportions of patients recruited from Japan + Korea and Not-Japan + Korea, with no trends across active treatment arms (Table 4). The most commonly reported treatment-related AEs in Japan + Korea were the well described local ICS effects of candidiasis and dysphonia, which were also among the most common treatment-related AEs in the Not-Japan + Korea cohort. For other potential class-related effects, the incidence of AEs related to blood glucose or potassium was low in the patients recruited from Japan + Korea and Not-Japan + Korea. Changes in heart rate ≥6 bpm relative to placebo were uncommon. Changes from baseline in the maximum post-baseline QTcF were consistent across all treatment arms, including placebo, in the Japan + Korea and Not-Japan + Korea patients. The incidence of ECG abnormalities of potential clinical importance did not increase for any post-dose assessment relative to pre-dose assessments for any treatment arm in any population.

**Table 4:** Incidence of on-treatment AEs by treatment group and patient cohort (Safety population)

	Incidence of AEs, n/N (%)				
	Placebo	FF/VI 100/25 μg OD	FF/VI 200/25 μg OD	FF 100 μg OD	FF 200 μg OD
Range of treatment duration of studies integrated (weeks)	8–12	12–76	24	8–76	8–24
On-treatment All AEs					
Japan + Korea	12/29 (41)	37/47 (79)*	9/14 (64)*	38/55 (69)	16/22 (73)
Not-Japan + Korea	87/375 (23)	658/1,163 (57)	83/183 (45)	744/1,375 (54)	134/368 (36)
Overall	99/404 (25)	695/1,210 (57)	92/197 (47)	782/1,430 (55)	150/390 (38)
On-treatment Drug-Related AEs					
Japan + Korea	0/29 (0)	4/47 (9)*	3/14 (21)*	1/55 (2)	2/22 (9)
Not-Japan + Korea	6/375 (2)	79/1,163 (7)	14/183 (8)	90/1,375 (7)	17/368 (5)
Overall	6/404 (1)	83/1,210 (7)	17/197 (9)	91/1,430 (6)	19/390 (5)
On-treatment AEs Leading to Permanent Discontinuation of Investigative Product or Withdrawal from the Study					
Japan + Korea	0/29 (0)	3/47 (6)*	1/14 (7)*	2/55 (4)	1/22 (5)
Not-Japan + Korea	1/375 (<1)	15/1,163 (1)	6/183 (3)	21/1,375 (2)	4/368 (1)
Overall	1/404 (<1)	18/1,210 (1)	7/197 (4)	23/1,430 (2)	5/390 (1)
On-treatment Non-fatal Serious AEs					
Japan + Korea	0/29 (0)	1/47 (2)*	0/14 (0)*	1/55 (2)	0/22 (0)
Not-Japan + Korea	0/375 (0)	39/1,163 (3)	6/183 (3)	29/1,375 (2)	1/368 (<1)
Overall	0/404 (0)	40/1,210 (3)	6/197 (3)	30/1,430 (2)	1/390 (<1)
On-treatment Fatal AEs					
Japan + Korea	0/29 (0)	0/47 (0)*	0/14 (0)*	0/55 (0)	0/22 (0)
Not-Japan + Korea	0/375 (0)	1/1,163 (<1)	0/183 (0)	1/1,375 (<1)	0/368 (0)
Overall	0/404 (0)	1/1,210^†^(<1)	0/197 (0)	1/1,430 (<1)	0/390 (0)

*AE* adverse event, *FF* fluticasone furoate, *OD* once daily, *VI* vilanterol

Safety population consists of data from studies HZA106827, HZA106829, HZA106837, FFA109685, and FFA109687. *Only patients recruited from Japan; ^†^One additional patient (South East Asian) died during follow-up (91 days after withdrawal)

There were two SAEs (both subarachnoid hemorrhage) in Japan + Korea patients (FF/VI 100/25 μg *N* = 1; FF 100 μg N = 1), which were not considered treatment related. In the Overall population, the most frequent SAE was asthma exacerbation (FF/VI 100/25 μg *N* = 11; FF 100 μg *N* = 9; FF 200 μg *N* = 1). For the Asthma Composite Endpoint, no events (asthma-related hospitalizations, intubations, and deaths) were reported for patients recruited from Japan + Korea, and the incidence was <1 % in each treatment arm in Not-Japan + Korea patients. The incidence of respiratory-related SAEs and pneumonia-related events was low in both the Japan + Korea and Not-Japan + Korea cohorts.

**Fig. 2:**
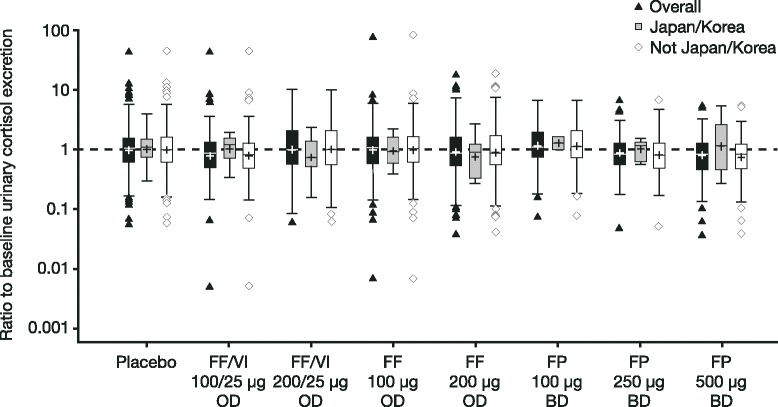
24h urinary cortisol excretion at the end of treatment relative to baseline (Urinary Cortisol population). Urinary cortisol population consists of data from studies HZA106827, HZA106829, FFA109685, and FFA109687. Number of patients analyzed in a) Overall population: *N* = 258 placebo, *N* = 153 FF/VI 100/25 μg OD, *N* = 140 FF/VI 200/25 μg OD, *N* = 301 FF 100 μg OD, *N* = 270 FF 200 μg OD, *N* = 70 FP 100 μg BD, *N* = 70 FP 250 μg BD, *N* = 123 500 μg BD; b) Japan/Korea cohort: *N* = 14 placebo, *N* = 12* FF/VI 100/25 μg OD, *N* = 10* FF/VI 200/25 μg OD, *N* = 21 FF 100 μg OD, *N* = 14 FF 200 μg OD, *N* = 2 FP 100 μg BD, *N* = 7 FP 250 μg BD, *N* = 8* FP 500 μg BD; c) Not-Japan/Korea cohort: *N* = 244 placebo, *N* = 141 FF/VI 100/25 μg OD, *N* = 130 FF/VI 200/25 μg OD, *N* = 280 FF 100 μg OD, *N* = 256 FF 200 μg OD, *N* = 68 FP 100 μg BD, *N* = 63 FP 250 μg BD, *N* = 115 FP 500 μg OD. See Fig. 1 for details of parameters represented by the box plot. *Patients from Japan only. *BD* twice daily, *FP* fluticasone propionate, *OD* once daily, *FF* fluticasone furoate, *VI* vilanterol

The urinary cortisol population comprised 1,385 patients (88 [6 %] recruited from Japan + Korea). Geometric mean ratios of 24 h urinary cortisol excretion at the end of treatment relative to baseline were generally close to one (Fig. 2). Treatment comparisons for the Japan + Korea and Not-Japan + Korea cohorts were performed for FF/VI 100/25 μg versus FF 100 μg, FF/VI 200/25 μg versus FF 200 μg, and FF 100 μg or FF 200 μg versus placebo, and were not statistically significant (the 95 % confidence intervals encompassed one; Table 5). In the Overall population, only FF 200 μg versus placebo was associated with statistically lower cortisol ratios (16 % reduction in urine cortisol excretion [ratio of end of treatment to baseline]), which is unlikely to be clinically relevant (Table 5). No statistically significant differences in the treatment comparisons were observed between the Japan + Korea and the Not-Japan + Korea cohorts (Additional File 9).

**Table 5:** Treatment comparison of 24 h urinary cortisol excretion at end of treatment (nmol/24 h) (Urinary Cortisol population)

		Placebo	FF/VI 100/25 μg OD	FF/VI 200/25 μg OD	FF 100 μg OD	FF 200 μg OD
Overall	N	258	153	140	301	270
	n^†^	258	153	140	301	270
	LS geometric mean	64.29	56.01	56.90	61.43	54.10
	LS ratio to baseline	1.03	0.9	0.91	0.99	0.87
	Ratio to placebo (95 % CI)	—	—	—	0.96 (0.85–1.08)	0.84 (0.71–0.99)*
	Ratio to FF 100 μg (95 % CI)	—	0.91 (0.78–1.06)	—	—	—
	Ratio to FF 200 μg (95 % CI)	—	—	1.05 (0.88–1.26)	—	—
Japan + Korea	N	14	12	10	21	14
	n^†^	14	12	10	21	14
	LS geometric mean	71.40	68.27	47.52	66.13	49.20
	LS ratio to baseline	1.15	1.10	0.76	1.06	0.79
	Ratio to placebo (95 % CI)	–	–	–	0.93 (0.56–1.52)	0.69 (0.40–1.20)
	Ratio to FF 100 μg (95 % CI)	–	1.03 (0.61–1.74)	–	–	–
	Ratio to FF 200 μg (95 % CI)	–	–	0.97 (0.53–1.78)	–	–
Not-Japan + Korea	N	244	141	130	280	256
	n^†^	244	141	130	280	256
	LS geometric mean	63.73	55.11	58.00	61.09	54.34
	LS ratio to baseline	1.02	0.89	0.93	0.98	0.87
	Ratio to placebo (95 % CI)	–	–	–	0.96 (0.84–1.09)	0.85 (0.72–1.01)
	Ratio to FF 100 μg (95 % CI)	–	0.90 (0.77–1.06)	–	–	–
	Ratio to FF 200 μg (95 % CI)	–	–	1.07 (0.89–1.28)	–	–

*CI* confidence interval, *FF* fluticasone furoate, *LS* least-squares, *OD* once daily, *VI* vilanterol

Data from studies FFA109685, FFA109687, HZA106827, and HZA106829. Analysis performed using ANCOVA with covariates of study, gender, age, treatment group, geographical region, geographical region-by-treatment interaction (Japan + Korea and Not-Japan + Korea analysis only), and the log of baseline values. ^†^Number of patients with analyzable data at end of treatment; *P = 0.038; Overall FF 200 μg *vs* placebo

## Discussion

The ethnic sensitivity of the novel OD treatment FF/VI in East Asian asthma patients included in Phase IIb and Phase III clinical studies has been assessed in this analysis. The efficacy (trough FEV_1_) results in asthma patients in Japan and the safety profile observed in asthma patients in Japan + Korea did not indicate that a different clinical dose of FF/VI is required in East Asian asthma patients, relative to non-East Asian patients.

In both the Japan and Not-Japan cohorts, a notably higher proportion of patients receiving placebo withdrew due to lack of efficacy, relative to patients receiving FF/VI. The improvements in trough FEV_1_ at Week 12 show that asthma patients in Japan benefited from FF/VI 100/25 μg or FF/VI 200/25 μg, and that response to treatment in Japanese patients is similar to that seen in the global population, suggesting the doses used in the global population are appropriate for use in Japanese patients. Patients recruited from Japan had a lower placebo response than patients from Not-Japan, resulting in the observation of greater numerical improvements with FF/VI versus placebo in the Japan cohort. For the FF/VI 100/25 μg versus FF 100 μg treatment comparison, differences between populations were less pronounced. The results for FF/VI 200/25 μg OD versus FP 500 μg BD at Week 12 and Week 24 also demonstrate that the change from baseline data for trough FEV_1_ appeared to be similar between the Japan and Not-Japan asthma patients.

There was a trend towards a greater proportion of patients in Japan + Korea reporting on-treatment AEs with FF/VI or FF than in the Not-Japan + Korea cohort. This trend was also observed with placebo, which indicates that patients in Japan + Korea had a higher tendency to report AEs in general. Regional differences in AE reporting have been observed for other drugs [21]. Furthermore, there was no evidence of ethnic differences for treatment-related AEs or class-related AEs. There were no deaths or treatment-related SAEs in patients recruited from Japan + Korea. The slightly greater proportion of withdrawals due to AEs in the Japan + Korea versus Not-Japan + Korea patients should be interpreted with caution due to low patient numbers; generally, withdrawals due to AEs were low and there was no clear indication of an ethnic difference. The safety profile of FF/VI in this analysis is consistent with that reported previously for Japanese asthma patients from a 12-month safety study of FF/VI [22], as well as in non-Japanese asthma patients in other multiregional studies [23, 24].

Systemic exposure to high-dose ICS has been associated with unwanted changes in hypothalamic–pituitary–adrenal (HPA) axis function and cortisol suppression [25]. Previous studies in Japanese subjects [26, 27] and other multiregional studies [25, 28] have assessed the dose-dependent effect of inhaled FF (doses up to 4,000 μg administered as a single dose, including 800 μg OD in Japanese) on urine or plasma cortisol. In the current pooled analysis for the treatment comparison of urine cortisol effect (FF versus placebo, or FF/VI versus dose-matched FF), no statistically significant differences were observed between the Japan + Korea and the Not-Japan + Korea populations. In the Overall population of the current pooled safety analysis, the FF 200 μg treatment arm demonstrated a 16 % reduction in urine cortisol excretion (ratio of end of treatment to baseline) relative to placebo, which was statistically significant but unlikely to be clinically relevant. However, modeling of the plasma FF concentration effect on urine or serum cortisol in multinational populations [25] and in healthy Japanese subjects [26] has demonstrated that clinical doses of FF (≤200 μg) have not been associated with decreases in cortisol of clinical concern. In addition, the definitive study of the effect of FF/VI 100/25 μg and 200/25 μg on the HPA axis, in which 24-h serum cortisol was assessed in patients with asthma, did not show a significant reduction of cortisol levels relative to placebo with either strength of FF/VI [28].

The absence of treatment-related changes of concern in blood glucose or potassium concentrations for VI administered as FF/VI are consistent with previous reports [26, 29, 30] in healthy Western and Japanese subjects. In the current analysis, there was no indication that VI administered as FF/VI was related to ECG abnormalities, consistent with previous reports in healthy Japanese subjects [26] and a multiregional study [29], suggesting that the clinical dose of VI 25 μg does not have a clinically relevant impact on QT/QTc interval. Increases in heart rate are a known LABA effect [31] and the small increases from baseline in heart rate observed in the current analysis are consistent with the small, transient effects reported in other clinical studies with inhaled VI [23, 32].

The pharmacokinetics of FF and VI have been characterized during the FF/VI development program in healthy subjects, including East Asians [26], as well as in patients with asthma or COPD [33]. In healthy subjects, higher FF systemic exposure (<2-fold) following oral inhalation has been observed in East Asians than Caucasians [27], which may be related to differences in absorption from the lung [27]. However, as previously described, clinical doses of FF (≤200 μg) have not been associated with urine cortisol suppression of clinical concern in Japanese healthy subjects and asthma patients. Therefore, the modest increase in FF systemic exposure anticipated in East Asian asthma patients does not result in clinically significant effects on HPA axis function. As previously noted, effects potentially associated with systemic VI concentrations (effects on glucose, potassium, and cardiovascular effects) were limited and were not of clinical concern in Japan + Korea and Not-Japan + Korea patients with asthma.

The current analysis has some limitations. Only a limited total number of patients in Japan and Korea were included in these multiregional clinical studies and the Japan and Japan + Korea cohorts were not formally powered for statistical analysis. Furthermore, even though these were pre-specified, a number of subgroup analyses were performed and multiplicity issues should be considered when drawing inference from the results of these analyses. It is also important to consider the differences in duration of exposure to study drug across the five studies, in particular, the markedly lower number of patient-years of exposure to placebo that limits the ability to directly compare safety data. In addition, differences in study design restrict the treatment arms that could be pooled for statistical comparisons of efficacy data, preventing the statistical comparison of FF/VI 200/25 μg versus placebo.

## Conclusion

The totality of the data from the current analysis, taken together with previously published results in Japanese asthma patients and Japanese healthy subjects [22, 26, 27], provide confidence that the efficacy and safety of FF/VI is of low ethnic sensitivity and this supports the conclusion that the same clinical doses of FF/VI (100/25 μg OD and 200/25 μg OD) are appropriate for both East Asian and non-East Asian asthma patients.

### Availability of data

Further details of each of the 5 studies included in this analysis, and a summary of the efficacy and safety results obtained for each study, are provided in the Clinicaltrials.gov registry (https://clinicaltrials.gov) and the GlaxoSmithKline Clinical Study Register: 

HZA106827 – http://www.gsk-clinicalstudyregister.com/study/106827#ps

HZA106829 – https://clinicaltrials.gov/ct2/show/NCT01134042?term=HZA106829&rank=1

HZA106837 – https://clinicaltrials.gov/ct2/show/NCT01086384?term=106837&rank=1

FFA109685 – https://clinicaltrials.gov/ct2/show/NCT00603278?term=FFA109685&rank=1

FFA109687 – https://clinicaltrials.gov/ct2/show/NCT00603382?term=FFA109687&rank=1

The studies are also fully published in peer-reviewed journals (Bleecker et al. J Allergy Clin Immunol Pract. 2014;2:553–61. O'Byrne et al. Eur Respir J. 2014;43:773–82. Bateman et al. Thorax. 2014;69:312–9. Bleecker et al. Ann Allergy Asthma Immunol. 2012;109:353–8. Bateman et al. Respir Med. 2012;106:642–50).

## Additional files

Additional file 1:
**Profile of the three Phase III studies included in both the efficacy and safety analyses.** (DOCX 47.4 KB) Additional file 2:
**Profile of the two additional Phase IIb studies included in only the safety analyses.** (DOCX 25.6 KB) Additional file 3:
**List of Institutions and Independent Ethics Committees/Institutional Review Boards for Studies HZA106829, HZA106827, HZA106837, FFA109685, FFA109687 Study HZA106829: 63 Centres.** (DOCX 100 KB) Additional file 4:
**Adjusted treatment differences from baseline in trough FEV1 at Week 12 (Efficacy population).** (DOCX 1.49 MB) Additional file 5:
**Ease of use of the ELLIPTA® dry powder inhaler (DPI; ELLIPTA® is a trademark of the GlaxoSmithKline group of companies).** (DOCX 22.5 KB) Additional file 6:
**Summary of ease of use assessment and responses to questions on the ELLIPTA DPI by patients from Japan (HZA106827 ITT population).** (DOCX 25.2 KB) Additional file 7:
**Summary of exposure in each treatment arm (Safety population).** (DOCX 26.5 KB) Additional file 8:
**AEs reported by ≥3 patients in the Japan+Korea cohort of any relevant treatment arm and the corresponding incidence in the Not-Japan+Korea cohort and the Overall population (Safety population).** (DOCX 26.9 KB) Additional file 9:
**Region ratios of ‘treatment effect’ in Japan+Korea and Not-Japan+Korea patients, where ‘treatment effect’ is the comparison between treatment arms of 24h urinary cortisol excretion ratio (end of treatment/baseline; Urinary Cortisol population).** (DOCX 944 KB)

## Abbreviations

AE: Adverse event
ANCOVA: Analysis of covariance
BD: Twice daily
CI: Confidence interval
DPI: Dry powder inhaler
ECG: Electrocardiogram
FEV_1_: Forced expiratory volume in one second
FF: Fluticasone furoate
FP: Fluticasone propionate
HPA: Hypothalamic-pituitary-adrenal
ICS: Inhaled corticosteroid
ITT: Intent-to-treat
LABA: Long-acting β_2_-agonist
LS: Least-squares
min: Minimum
max: Maximum
OD: Once daily
QTcF: QT corrected using Frederica’s correction
SAE: Serious adverse event
SD: Standard deviation
SE: Standard error
VI: Vilanterol
