# Supplementary material for: Ethnic sensitivity assessment of fluticasone furoate/vilanterol in East Asian asthma patients from randomized double-blind multicentre Phase IIb/III trials
Source: BMC Pulm Med. 2015 Dec 24;15:165. doi: 10.1186/s12890-015-0159-z (PMC4690330; doi:10.1186/s12890-015-0159-z)
Supplement: Additional file 1: — Profile of the three Phase III studies included in both the efficacy and safety analyses. (DOCX 47.4 KB) [file 12890_2015_159_MOESM1_ESM.docx]

**Additional File 1 Profile of the three Phase III studies included in both the efficacy and safety analyses**

|  | Multiregional  HZA106827 | Multiregional  HZA106829 | Multiregional  HZA106837 |
| --- | --- | --- | --- |
| Study design and objectives | Phase III, multicenter, stratified, randomized, double-blind, placebo-controlled (with rescue medication), parallel group study to evaluate the efficacy and safety of FF/VI, FF, VI, and placebo when administered via ELLIPTA | Phase III, multicenter, stratified, randomized, double-blind, double-dummy, parallel group study to evaluate the efficacy and safety of FF/VI and FF when administered via ELLIPTA. The secondary objective of this study was to compare the efficacy of FF 200 μg administered OD each evening with FP 500 μg administered BD | Phase III, multicenter, randomized, double-blind, parallel group study. The primary objective of this study was to demonstrate that treatment with FF/VI administered via ELLIPTA significantly decreased the risk of severe asthma exacerbations as measured by time to first severe asthma exacerbation when compared with the same dose of FF alone |
| FF/VI, FF, or VI dose regimen | FF/VI 100/25 μg  FF 100 μg  Placebo  Administered OD (1 inhalation) in the evening | FF/VI 200/25 μg  FF 200 μg  Administered OD (1 inhalation) in the evening  FP 500 μg  Administered BD (1 inhalation) in the morning and evening | FF/VI 100/25 μg  FF 100 μg  Administered OD (1 inhalation) in the evening |
| Duration of treatment period | 12 weeks | 24 weeks | 24 weeks up to 76 weeks. The duration of the treatment period is variable. The duration of the study depended on the number of events (number of patients with one or more severe asthma exacerbations) that occurred |
| Duration of run-in and follow-up | Run-in: 4 weeks  Follow-up: 2 weeks | Run-in: 4 weeks  Follow-up: 1 week | Run-in: 2 weeks  Follow-up: 1 week |
| Study population | Patients with a diagnosis of asthma as defined by the National Institutes of Health [1] | Patients with a diagnosis of asthma as defined by the National Institutes of Health [1] | Patients with a diagnosis of asthma as defined by the National Institutes of Health [1] |
| Countries | Germany, Japan, Poland, Romania, Ukraine, and the USA | Germany, Japan, Poland, Romania, Russian Federation, and the USA | Argentina, Australia, Germany, Japan, Mexico, Philippines, Poland, Romania, Russian Federation, Ukraine, and the USA |
| Total randomized patients/ ITT population | 610 patients randomized to treatment and 609 received at least one dose of study medication: ITT population | 586 patients randomized to treatment, and received at least one dose of study medication: ITT population | 2,020 patients randomized and 2,019 received at least one dose of study medication: ITT population |
| Race/ancestry/heritage | N = 609  White n = 512 (84%); Japanese  n = 50 (8%) from Japan; South East Asian n = 1 (<1%); African American n = 43 (7%); American Indian or Alaska Native n = 1 (<1%); Mixed Race n = 2 (<1%) | N = 586  White n = 492 (84%); Japanese  n = 36 (6%) from Japan; East Asian* n = 3 (<1%) from US; South East Asian n = 1 (<1%); African American n = 51 (9%); American Indian or Alaska Native n = 1 (<1%); Mixed Race n = 2 (<1%) | N = 2019  White n = 1483 (73%); Japanese  n = 62 (3%) from Japan; East Asian* n = 1 (<1%); Central/South Asian n = 2 (<1%); South East Asian n = 157 (8%); African American n = 87 (4%); American Indian or Alaska Native n = 13 (<1%); Mixed Race n = 212 (11%); Native Hawaiian or other Pacific Islander n = 2 (<1%) |
| Baseline ICS exposure and disease status | At the time of recruitment, patients must have been currently receiving FP 200–500 μg daily OR equivalent ICS daily OR FP/Salmeterol 200/100 μg OR equivalent ICS/LABA. Patients had to have a best pre-bronchodilator FEV_1_ of 40–90% of the predicted normal^†^ value at Visit 1. Patients required to be symptomatic at entry to randomization period with an asthma symptom score of ≥1 and/or daily use of short-acting β_2_-agonist on ≥4 of the last 7 days during run-in period | At the time of recruitment, patients must have been currently receiving FP 1,000 μg daily OR equivalent ICS daily OR FP/Salmeterol 500/100 μg daily OR equivalent ICS/LABA. Patients had to have a best pre-bronchodilator FEV_1_ of 40-90% of the predicted normal^†^ value at Visit 1. Patients required to be symptomatic at entry to randomization period with an asthma symptom score of ≥3 and/or daily use of short-acting β_2_-agonist on ≥4 of the last 7 days during run-in period | At the time of recruitment, patients must have been currently receiving FP 200–1,000 μg daily OR FP/Salmeterol 200/100–500/100 μg daily OR equivalent ICS OR ICS/LABA. Patients had to have a best pre-bronchodilator FEV_1_ of 50–90% of the predicted normal^†^ value at Visit 1. Patients required to be symptomatic at entry to randomization period with an asthma symptom score of 1 or greater and/or daily salbutamol use on ≥3 of the last 7 consecutive days of run-in AND must have experienced an exacerbation requiring systemic corticosteroids OR hospital visit within the last 12 months |
| Concomitant asthma medications | Patients must have been maintained on a stable dose of the same ICS for four weeks prior to Visit 1 and throughout the run-in period. Salbutamol was permitted for symptom relief | Patients must have been maintained on a stable dose of the same ICS for four weeks prior to Visit 1 and throughout the run-in period. Salbutamol was permitted for symptom relief | Patients must have been maintained on a stable dose of the same ICS for four weeks prior to Visit 1 and throughout the run-in period. Salbutamol was permitted for symptom relief |

BD, twice daily; FEV_1_, forced expiratory volume in one second; FF, fluticasone furoate; FP, fluticasone propionate; ICS, inhaled corticosteroid; ITT, intent-to-treat; LABA, long-acting β_2_-agonist; OD, once daily; VI, vilanterol.

*East Asian: all patients of East Asian ancestry excluding patients of Japanese ancestry; ^†^Predicted spirometry values were based upon NHANES III [2]; if a patient was of Asian race then the Asian adjustment was used.

1. National Institutes of Health (NIH). Guidelines for the Diagnosis and Management of Asthma - Expert Panel Report 3 2007. U.S. Department of Health and Human Services, Bethesda, MD; 2007. <http://www.nhlbi.nih.gov/guidelines/asthma/asthgdln.pdf>.
2. Hankinson JL, Kawut SM, Shahar E, Smith LJ, Stukovsky KH, Barr RG. Performance of American Thoracic Society-recommended spirometry reference values in a multiethnic sample of adults: the multi-ethnic study of atherosclerosis (MESA) lung study. Chest. 2010;137:138-45.
