# Supplementary material for: Ethnic sensitivity assessment of fluticasone furoate/vilanterol in East Asian asthma patients from randomized double-blind multicentre Phase IIb/III trials
Source: BMC Pulm Med. 2015 Dec 24;15:165. doi: 10.1186/s12890-015-0159-z (PMC4690330; doi:10.1186/s12890-015-0159-z)
Supplement: Additional file 2: — Profile of the two additional Phase IIb studies included in only the safety analyses. (DOCX 25.6 KB) [file 12890_2015_159_MOESM2_ESM.docx]

**Additional File 2 Profile of the two additional Phase IIb studies included in only the safety analyses**

|  | Multiregional  FFA109685 | Multiregional  FFA109687 |
| --- | --- | --- |
| Study design  and objectives | Phase IIb, multicenter, randomized, double-blind, double-dummy, placebo-controlled, parallel-group, dose-ranging study to evaluate the efficacy and safety of FF administered OD and FP BD compared with placebo for 8 weeks | Phase IIb, multicenter, randomized, double-blind, double-dummy, placebo-controlled, parallel-group, dose-ranging study to evaluate the efficacy and safety of FF administered OD and FP BD compared with placebo for 8 weeks |
| FF/VI, FF, or VI dose  regimen (integrated arms only) | FF 100 μg, 200 μg  Administered OD (one inhalation) in the evening  FP 250 μg BD  Placebo BD  Administered BD (one morning and one evening inhalation) | FF 100 μg, 200 μg  Administered OD (one inhalation) in the evening  FP 100 μg BD  Placebo BD  Administered BD (one morning and one evening inhalation) |
| Duration of treatment period | 8 weeks | 8 weeks |
| Duration of run-in and follow-up | Run-in: 4 weeks  Follow-up: 1 week | Run-in: 4 weeks  Follow-up: 1 week |
| Study population | Patients with a diagnosis of asthma as defined by the National Institutes of Health [1] | Patients with a diagnosis of asthma as defined by the National Institutes of Health [1] |
| Countries | Canada, Estonia, Germany, Greece, Korea, Mexico, Philippines, Poland, Slovakia, Romania, Russian Federation, South Africa, USA | Bulgaria, Slovakia, Canada, France, Germany, Sweden, Korea, Mexico, Peru, Philippines, Poland, Estonia, Russian Federation, USA |
| Total randomized patients/ ITT population | 622 patients randomized to treatment and 615 (99%) received at least one dose of study medication and were included in the ITT population | 601 patients randomized to treatment and 598 (>99%) received at least one dose of study medication and were included in the ITT population |
| Race/ancestry/heritage | N = 615  Arms integrated in Safety Analyses (N = 413):  Placebo: N = 107; White n = 62 (58%); East Asian*  n = 9 (8%, eight from Korea and one from Philippines); Central/South Asian n = 1 (<1%); South East Asian  n = 16 (15%); African American n = 5 (5%); Mixed Race n = 14 (13%)  FF 100 μg OD: N = 105; White n = 64 (61%); Japanese n = 1 (<1%) East Asian* n = 7 (7%) from Korea; Central/South Asian n = 1 (<1%); South East Asian n = 16 (15%); African American n = 2 (2%); American Indian n = 1 (<%); Mixed Race n = 13 (12%)  FF 200 μg OD: N = 101; White n = 65 (65%); East Asian* n = 7 (7%) from Korea; South East Asian n = 16 (16%); Mixed Race n = 13 (13%)  FP 250 μg BD: N = 100; White n = 61 (61%); East Asian* n = 7 (7%) from Korea; South East Asian n = 16 (16%); African American n = 3 (3%); Mixed Race  n = 13 (13%) | N = 598  Arms integrated in Safety Analyses (N = 401):  Placebo: N = 94; White n = 69 (73%); East Asian*  n = 2 (2%) from Korea; South East Asian n = 5 (5%); African American n = 5 (5%); American Indian n = 5 (5%); Mixed Race n = 8 (9%)  FF 100 μg OD: N = 110; White n = 76 (69%); East Asian* n = 2 (2%) from Korea; Central/South Asian n = 1 (<1%); South East Asian n = 7 (6%); African American n = 8 (7%); American Indian n = 6 (5%); Mixed Race n = 10 (9%)  FF 200 μg OD: N = 95; White n = 64 (67%); East Asian* n = 4 (4%) from Korea; South East Asian  n = 6 (6%); African American n = 6 (6%); American Indian n = 6 (6%); Mixed Race n = 9 (9%)  FP 100 μg BD: N = 102; White n = 74 (73%); East Asian* n = 3 (3%) from Korea; Central/South Asian n = 1 (<1%); South East Asian n = 6 (6%); African American n = 5 (5%); American Indian n = 5 (5%); Mixed Race n = 8 (8%) |
| Permitted asthma pharmacotherapies | Short-acting β_2_-agonists | Short-acting β_2_-agonists |
| Baseline ICS exposure | At the time of recruitment patients must have been currently receiving a stable dose of ICS equivalent to ≤FP 200 μg daily for 4 weeks prior to visit 1. Patients were maintained on this ICS throughout the run-in period | Patients must not have received any ICS for 6 weeks prior to Screening (10 weeks prior to first dose of investigational product) |
| Primary efficacy endpoints | Mean change from baseline to the end of the 8-week treatment period in trough FEV_1_ | Mean change from baseline to the end of the 8-week treatment period in trough FEV_1_ |
| Safety endpoints | Incidence of AEs; examination of the oropharynx for evidence of oral candidiasis; hematology, clinical chemistry, and urinalysis parameters; 24h urinary cortisol excretion assessment; vital signs (including pulse and blood pressure) | Incidence of AEs; examination of the oropharynx for evidence of oral candidiasis; hematology, clinical chemistry, and urinalysis parameters; 24h urinary cortisol excretion assessment; vital signs (including pulse and blood pressure) |

AE, adverse event; BD, twice daily; OD, once daily FEV_1_, forced expiratory volume in one second; FF, fluticasone furoate; FP, fluticasone propionate; ICS, inhaled corticosteroid; ITT, intent-to-treat; VI, vilanterol.

*East Asian: all patients of East Asian ancestry excluding patients of Japanese ancestry.
