# Supplementary material for: Ethnic sensitivity assessment of fluticasone furoate/vilanterol in East Asian asthma patients from randomized double-blind multicentre Phase IIb/III trials
Source: BMC Pulm Med. 2015 Dec 24;15:165. doi: 10.1186/s12890-015-0159-z (PMC4690330; doi:10.1186/s12890-015-0159-z)
Supplement: Additional file 3: — List of Institutions and Independent Ethics Committees/Institutional Review Boards for Studies HZA106829, HZA106827, HZA106837, FFA109685, FFA109687 Study HZA106829: 63 Centres. (DOCX 100 KB) [file 12890_2015_159_MOESM3_ESM.docx]

**Additional File 3 List of Institutions and Independent Ethics Committees/Institutional Review Boards for Studies HZA106829, HZA106827, HZA106837, FFA109685, FFA109687**

**Study HZA106829: 63 Centres**

| **HZA106829**  **Hospital/ Institution and Address** | **HZA106829**  **IEC/IRB Committee Chair and Name of Committee** |
| --- | --- |
| **Germany** |  |
| Praxis Dr. med. Wolfram Feussner, Schoenfelder Street 1, Kassel, Hessen, 34121, Germany. | Ethik-Kommission der Aerztekammer Schleswig, Holstein, Bismarckallee 8–12, Bad Segeberg, 23795, Germany.  Chairperson: Hintze, Gerhard |
| Hamburger Institut für Therapieforschung GmbH, Colonnaden 72, Hamburg, Germany, North, 20354, Germany. | Ethik-Kommission der Aerztekammer Schleswig, Holstein, Bismarckallee 8–12, Bad Segeberg, 23795, Germany.  Chairperson: Hintze, Gerhard |
| Praxis Dr. med. Matthias John, Auguststrasse 23, Schwedt, Brandenburg, 16303, Germany. | Ethik-Kommission der Aerztekammer Schleswig, Holstein, Bismarckallee 8–12, Bad Segeberg, 23795, Germany.  Chairperson: Hintze, Gerhard |
| Praxis Dr. med. Claus Keller, Usinger Strasse 5, Frankfurt, Hessen, 60389, Germany. | Ethik-Kommission der Aerztekammer Schleswig, Holstein, Bismarckallee 8–12, Bad Segeberg, 23795, Germany.  Chairperson: Hintze, Gerhard |
| KLB Gesundheitsforschung Luebeck GmbH, Pferdemarkt 6-8, Luebeck, Schleswig-Holstein, 23552, Germany. | Ethik-Kommission der Aerztekammer Schleswig, Holstein, Bismarckallee 8–12, Bad Segeberg, 23795, Germany.  Chairperson: Hintze, Gerhard |
| Facharztzentrum in den Main-Kinzig Kliniken Gelnhausen, Haus E, Ebene 10, Herzbachweg 14, Gelnhausen, Hessen, 63571, Germany. | Ethik-Kommission der Aerztekammer Schleswig, Holstein, Bismarckallee 8–12, Bad Segeberg, 23795, Germany.  Chairperson: Hintze, Gerhard |
| Gem Praxis Dres., Volgmann und Hoffmann, Tulpenstrasse 1, Hannover, Niedersachsen, 30167, Germany. | Ethik-Kommission der Aerztekammer Schleswig, Holstein, Bismarckallee 8–12, Bad Segeberg, 23795, Germany.  Chairperson: Hintze, Gerhard |
| Pneumologisches, Forschungsinstitut, Hohegeest GbR, Norderstrasse. 12, Geesthacht, Schleswig-Holstein, 21502, Germany. | Ethik-Kommission der Aerztekammer Schleswig, Holstein, Bismarckallee 8–12, Bad Segeberg, 23795, Germany.  Chairperson: Hintze, Gerhard |
| **Japan** |  |
| Hiroshima Allergy and Respiratory Clinic, 6F, Daiichi Teraoka Building, 9-28, 1-chome, Hikarimachi, Higashi-ku, Hiroshima-shi, Hiroshima, 732-0052, Japan. | Hiroshima Allergy and Respiratory Clinic, 6F, Daiichi Teraoka Building, 9-28, 1-chome, Hikarimachi, Higashi-ku, Hiroshima-shi, Hiroshima, 732-0052, Japan.  Chairperson: Maeda, Akihiro |
| Okusawa Hospital & Clinics, 2-11-11, Okusawa, Setagaya-ku, Tokyo, 158-0083, Japan. | Medical Corporation Shinkenkai Suzuki Internal Medicine Cardiovascular Medicine, 202, Maison de Statue, 1-39-5, Sangenjaya, Setagaya-ku, Tokyo, 154-0024, Japan.  Chairperson: Nakajima, Osamu |
| Kawai Chest Clinic, 43-3, Koyamakitaohnocho, Kita-ku, Kyoto-city, Kyoto, 603-8161, Japan. | Kawai Chest Clinic, 43-3, Koyamakitaohnocho, Kita-ku, Kyoto-city, Kyoto, 603-8161, Japan.  Chairperson: Sawaoka, Heiwa |
| Seirei Hamamatsu General Hospital, 2-12-12, Sumiyoshi, Naka-Ku, Hamamatsu-shi, Shizuoka, 430-8558, Japan. | Seirei Hamamatsu General Hospital, 2-12-12, Sumiyoshi, Naka-Ku, Hamamatsu-shi, Shizuoka, 430-8558, Japan.  Chairperson: Nakamura, Hidenori |
| Medical Corporation HSR Nakamura Clinic, 4-2-1, Iso, Urasoe-city, Okinawa, 901-2132, Japan. | Kobari General Hospital, 29-1, Yokochi, Noda-shi, Chiba, 278-8501, Japan.  Chairperson: Ninomiya, Hiroki |
| Sakaide City Hospital, 6-43, Bunkyo-cho 1-chome, Sakaide-city, Kagawa, 762-0031, Japan. | Sakaide City Hospital, 6-43, Bunkyo-cho 1-chome, Sakaide-city, Kagawa, 762-0031, Japan.  Chairperson: Taoka, Teruhisa |
| Sano Toranomon Clinic, 102, Toranomonhousou BLD, 1-20-3, NishiShinbashi, Minato-ku, Tokyo, Japan, 105-0003. | Sekino Hospital, 3-28-3, Ikebukuro, Toshima-ku, Tokyo, Japan, 171-0014.  Chairperson: Akiyama, Kenji |
| National Hospital Organization Fukuoka National Hospital, 4-39-1, Yakatabaru, Minami-ku, Fukuoka-city, Fukuoka, 811-1394, Japan. | National Hospital Organization Fukuoka National Hospital, 4-39-1, Yakatabaru, Minami-ku, Fukuoka-city, Fukuoka, 811-1394, Japan.  Chairperson: Odajima, Hiroshi |
| Tanaka Clinic, 1F, Tanaka Kosan Building, 1-192, Miyake, Shikama-ku, Himeji-city, Hyogo, 672-8048, Japan. | Nose Clinic, 1-5-10, Nishitenma, Kita-Ku, Osaka-Shi, Osaka, 530-0047, Japan.  Chairperson: Hoshi, Mitsuru |
| **Poland** |  |
| NZOZ Allmed s.c., ul. Pilsudzkiego 2, Piekary Slaskie, 41-940, Poland. | Komisja Bioetyczna przy Okregowej Izbie Lekarskiej, ul. Swietojanska 7, Bialystok, 15-082, Poland.  Chairperson: Pedich, Wojciech |
| Specjalistyczna Praktyka Lekarska, ul.Zygmunta Starego 16a, Gliwice, 44-100, Poland. | Komisja Bioetyczna przy Okregowej Izbie Lekarskiej, ul. Swietojanska 7, Bialystok, 15-082, Poland.  Chairperson: Pedich, Wojciech |
| Krakowski Szpital Specjalistyczny im Jana Pawla II, II Oddzial Chorob Pluc, ul. Pradnicka 80, Krakow, 31-202, Poland. | Komisja Bioetyczna przy Okregowej Izbie Lekarskiej, ul. Swietojanska 7, Bialystok, 15-082, Poland.  Chairperson: Pedich, Wojciech |
| Medcare NZOZ Przychodnia Lekarska, Suchanino ul.Otwarta 4, Gdansk, 80-169, Poland. | Komisja Bioetyczna przy Okregowej Izbie Lekarskiej, ul. Swietojanska 7, Bialystok, 15-082, Poland.  Chairperson: Pedich, Wojciech |
| NZOZ Osrodek Terapii Uzaleznien, Rydygiera 6, Dzialdowo, 13-200, Poland. | Komisja Bioetyczna przy Okregowej Izbie Lekarskiej, ul. Swietojanska 7, Bialystok, 15-082, Poland.  Chairperson: Pedich, Wojciech |
| Prywatny Gabinet Internistyczno-Alergologiczny, Ogrodowa 5, Bialystok, 15-010, Poland. | Komisja Bioetyczna przy Okregowej Izbie Lekarskiej, ul. Swietojanska 7, Bialystok, 15-082, Poland.  Chairperson: Pedich, Wojciech |
| Medical Centre “Kardiotel", Department of Allergology and Lung Diseases, Jana z Kolna 16, Sopot, 81-741, Poland. | Komisja Bioetyczna przy Okregowej Izbie Lekarskiej, ul. Swietojanska 7, Bialystok, 15-082, Poland.  Chairperson: Pedich, Wojciech |
| **Romania** |  |
| Centrul Medical Valahia SRL, Street Mihai Bravu No 2, Ploiesti, 100550, Romania. | Comisia Naţională de Etică, 48 Aviator Sanatescu Street, District 1, Bucuresti, 011478, Romania.  Chairperson: Dumitrescu, Sava |
| Spitalul Clinic de Boli Infectioase si Pneumoftiziologie "Dr Victor Babes", Street Gheorghe Adam No 13, Timisoara, 300310, Romania. | Comisia Naţională de Etică, 48 Aviator Sanatescu Street, District 1, Bucuresti, 011478, Romania.  Chairperson: Dumitrescu, Sava |
| Spitalul de Pneumoftiziologie Oradea, Street Izvorului No 57, Oradea, 410176, Romania. | Comisia Naţională de Etică, 48 Aviator Sanatescu Street, District 1, Bucuresti, 011478, Romania.  Chairperson: Dumitrescu, Sava |
| Spitalul Judetean de Urgenta "Sf. Ioan cel nou", Strada 1 Decembrie 1918 No 21, Suceava, 720284, Romania. | Comisia Naţională de Etică, 48 Aviator Sanatescu Street, District 1, Bucuresti, 011478, Romania.  Chairperson: Dumitrescu, Sava |
| Spitalul Clinic Colentina, Sos. Street Stefan Cel Mare No 19-21, Bucharest, 020125, Romania. | Comisia Naţională de Etică, 48 Aviator Sanatescu Street, District 1, Bucuresti, 011478, Romania.  Chairperson: Dumitrescu, Sava |
| Spitalul Clinic de Boli Infectioase si Tropicale "Dr. Victor Babes", Soseaua Street Mihai Bravu No 281-283, Sector 3, Bucharest, 030317, Romania. | Comisia Naţională de Etică, 48 Aviator Sanatescu Street, District 1, Bucuresti, 011478, Romania.  Chairperson: Dumitrescu, Sava |
| Spitatul Clinic De Pneumoftiziologie Lasi, Street Iosif Cihac No 30, Lasi, 700115, Romania. | Comisia Naţională de Etică, 48 Aviator Sanatescu Street, District 1, Bucuresti, 011478, Romania.  Chairperson: Dumitrescu, Sava |
| Spitalul Clinic de boli infectioase si pneumoftiziologie "Dr Victor Babes", Clinica de Pneumologie, Street Gheorghe Adam No 13, Timisoara, 300310, Romania. | Comisia Naţională de Etică, 48 Aviator Sanatescu Street, District 1, Bucuresti, 011478, Romania.  Chairperson: Dumitrescu, Sava |
| **Russian Federation** |  |
| Saint Petersburg Non-Profit Institution “Metropolitan Out-Patient Department”, 22 building 2, Tramvayniy pr, St. Petersburg, 198216, Russian Federation. | Saint Petersburg Non-Profit Institution “Metropolitan Out-Patient Department”, 22 building 2, Tramvayniy pr, St. Petersburg, 198216, Russian Federation.  Chairperson: Kolesnikova, Lyudmila Mikhaylovna |
| Federal State Institution of Science “Kazan Scientific Research Institute of Epidemiology and Microbiology of Respotrebnadzor”, 67 Bolshaya Krasnaya, Kazan, 420015, Russian Federation. | Federal State Institution of Science “Kazan Scientific Research Institute of Epidemiology and Microbiology of Respotrebnadzor”, 67 Bolshaya Krasnaya, Kazan, 420015, Russian Federation.  Chairperson: Sergey Valerievich, Andreev |
| Russian Academy of Advanced Medical Studies of Federal Agency of Health and Social Development, Allergology Department based at City Hospital No 52 of Moscow Healthcare Committee, Building 3, ul. Pekhotnaya 3, Moscow, 123182, Russian Federation. | Ethics Committee attached to State Educational Institution of Additional Professional Education of Russian Medical Academy of Post-Graduate Education, 2/1, Barricadnaya Street, Moscow, 123995, Russian Federation.  Chairperson: Ermolov, Alexander |
| Clinical Hospital No 2, d. 24, UI Popova Street, Yaroslavl, 150010, Russian Federation. | Local Ethics Committee attached to Yaroslavl Medical Academy, 5, Revolyuzionnaya Street, Yaroslavl, 150000, Russian Federation.  Chairperson: Chizhov, Petr |
| State Educational Institution of the Highest Professional Education “Ural State Medical Academy of Roszdrav”, based on LLC Medical Association"Novaya Bolnitsa", 29, Zavodskaya Street, Ekaterinburg, 620109, Russian Federation. | State Educational Institution of the Highest Professional Education “Ural State Medical Academy of Roszdrav”, 3a Repina Street, Ekateinburg, 620219, Russian Federation.  Chairperson: Rudnov, Vladimir A. MD, PhD |
| Central Clinical Hospital No 1 RZHD, Pulmonology Department, Volokolamskoe Shosse, 84, Moscow, 125367, Russian Federation. | Central Hospital of Railways Ministry, Volokolamskoe Shosse, 84, Moscow, 125367, Russian Federation.  Chairperson: Andrey, Dmitriev |
| State Educational Institution of Higher Professional Education ”Smolensk State Medical Academy of Roszdrav” based on Municipal Medical and Prophylactic Institution "Clinical Hospital No 1", Frunze Street 40, Smolensk, 2140018, Russian Federation. | State Educational Institution of Higher Professional Education ”Smolensk State Medical Academy of Roszdrav”, Krupskoy Street 40, Smolensk, 214019, Russian Federation.  Chairperson: Aleksandr, Litvinov |
| Moscow State Healthcare Institution, City Clinical Hospital No 13, 1/1 Volozavodskaya Street, Moscow, 115280, Russian Federation. | Moscow State Healthcare Institution, City Clinical Hospital No 13, 1/1 Volozavodskaya Street, Moscow, 115280, Russian Federation.  Chairperson: Meshkina, O.B. |
| Moscow State Healthcare Institution, City Clinical Hospital No 11, 6 Dvintsev Street, Moscow, 127018, Russian Federation. | Moscow State Healthcare Institution, City Clinical Hospital No 11, 6 Dvintsev Street, Moscow, 127018, Russian Federation.  Chairperson: Evgeniy, Ivanovich |
| City Clinical Hospital No 7, MMA, 4 Kolomenskii Proezd Street, Moscow, 115446, Russian Federation. | Local Ethics Committee attached to City Clinical Hospital No 7, MMA, 4 Kolomenskii Proezd Street, Moscow, 115446, Russian Federation.  Chairperson: Vlasenko, Nadezhda A |
| Ryazan State Medical University, 9, Vysokovoltnaya Street, Ryazan, 390026, Russian Federation. | Ethics Committee attached to Ryazan State Medical University, 9, Vysokovoltnaya Street, Ryazan, 390026, Russian Federation.  Chairperson: Abrosimov, Vladimir |
| **United States** |  |
| Baker Allergy, Asthma and Dermatology Research Center, LLC, Suite 165, 3975 Southwest Mercantile Drive, Lake Oswego, Oregon, 97035, United States. | Quorum Review Institutional Review Board, Suite 1000, 1601 Fifth Avenue, Seattle, Washington, 98101, United States.  Chairperson: Kelley, David B. |
| Bernstein Clinical Research Center, LLC, 8444 Winton Road, Cincinnati, Ohio, 4523, United States. | Quorum Review Institutional Review Board, Suite 1000, 1601 Fifth Avenue, Seattle, Washington, 98101, United States.  Chairperson: Kelley, David B. |
| Pioneer Research Solutions, Inc., 3533 Town Center Boulevard, Suite 100, Sugar Land, Texas, 77479, United States. | Quorum Review Institutional Review Board, Suite 1000, 1601 Fifth Avenue, Seattle, Washington, 98101, United States.  Chairperson: Kelley, David B. |
| IPS Research Company, 1111 North Lee, Suite 400, Oklahoma City, Oklahoma, 73103, United States. | Quorum Review Institutional Review Board, Suite 1000, 1601 Fifth Avenue, Seattle, Washington, 98101, United States.  Chairperson: Kelley, David B. |
| Regional Allergy and Asthma Consultants, P.A, 14 McDowell Street, Asheville, North Carolina, 28801, United States. | Quorum Review Institutional Review Board, Suite 1000, 1601 Fifth Avenue, Seattle, Washington, 98101, United States.  Chairperson: Kelley, David B. |
| Clinical Research Institute of Southern Oregon, PC, Suite B, 3860 Crater Lake Avenue, Medford, Oregon, 97504, United States. | Quorum Review Institutional Review Board, Suite 1000, 1601 Fifth Avenue, Seattle, Washington, 98101, United States.  Chairperson: Kelley, David B. |
| Trinity Health and Wellness, Quality Control Research, Inc., Suite 220, 576 N. Sunrise Avenue, Roseville, California, 95661, United States. | Quorum Review Institutional Review Board, Suite 1000, 1601 Fifth Avenue, Seattle, Washington, 98101, United States.  Chairperson: Kelley, David B. |
| Austin Center for Clinical Research, Suite 202, 4201 Marathon Boulevard, Austin, Texas, 78756, United States. | Quorum Review Institutional Review Board, Suite 1000, 1601 Fifth Avenue, Seattle, Washington, 98101, United States.  Chairperson: Kelley, David B. |
| Allergy and Asthma Care Center of Southern California, Suite 209, 3816 Woodruff Avenue, Long Beach, California, 90808, United States. | Quorum Review Institutional Review Board, Suite 1000, 1601 Fifth Avenue, Seattle, Washington, 98101, United States.  Chairperson: Kelley, David B. |
| Allergy and Asthma Research Center of El Paso, PA, Suites 100 and 203, 10470 Vista Del Sol., El Paso, Texas, 79925, United States. | Quorum Review Institutional Review Board, Suite 1000, 1601 Fifth Avenue, Seattle, Washington, 98101, United States.  Chairperson: Kelley, David B. |
| Emerald Coast Research Associates, Suite B, 221 East 23rd Street, Panama City, Florida, 32405, United States. | Quorum Review Institutional Review Board, Suite 1000, 1601 Fifth Avenue, Seattle, Washington, 98101, United States.  Chairperson: Kelley, David B. |
| Sneeze, Wheeze and Itch Associates, LLC, 2010 Jacobssen Drive, Normal, Illinois, 61761, United States. | Quorum Review Institutional Review Board, Suite 1000, 1601 Fifth Avenue, Seattle, Washington, 98101, United States.  Chairperson: Kelley, David B. |
| Florida Center for Allergy and Asthma Research, Suite 202, 9035 Sunset Drive, Miami, Florida, 33173, United States. | Quorum Review Institutional Review Board, Suite 1000, 1601 Fifth Avenue, Seattle, Washington, 98101, United States.  Chairperson: Kelley, David B. |
| Integrated Research Group Inc., Suites 101/201/202/203/302, 4646 Brockton Avenue, Riverside, California, 92506, United States. | Quorum Review Institutional Review Board, Suite 1000, 1601 Fifth Avenue, Seattle, Washington, 98101, United States.  Chairperson: Kelley, David B. |
| Pediatric Care Medical Group Inc., Suite 263/278/400, 17822 Beach Boulevard, Huntington Beach, California, 92647, United States. | Quorum Review Institutional Review Board, Suite 1000, 1601 Fifth Avenue, Seattle, Washington, 98101, United States.  Chairperson: Kelley, David B. |
| Respiratory Diseases of Children and Adolescents, Suite 330 Baptist Medical Plaza Building D, 3366 Northwest Expressway, Oklahoma City, Oklahoma, 73112, United States. | Quorum Review Institutional Review Board, Suite 1000, 1601 Fifth Avenue, Seattle, Washington, 98101, United States.  Chairperson: Kelley, David B. |
| Carolina Research, 2227 Saint Matthews Road, Orangeburg, South Carolina, 29118, United States. | Quorum Review Institutional Review Board, Suite 1000, 1601 Fifth Avenue, Seattle, Washington, 98101, United States.  Chairperson: Kelley, David B. |
| Innovative Research of West Florida, Inc., 1573 South Fort Harrison Avenue, Clearwater, Florida, 33756, United States. | Quorum Review Institutional Review Board, Suite 1000, 1601 Fifth Avenue, Seattle, Washington, 98101, United States.  Chairperson: Kelley, David B. |
| Southern California Institute for Respiratory Diseases, Inc., Suite 965 West, 8635 West Third Street, Los Angeles, California, 90048, United States. | Quorum Review Institutional Review Board, Suite 1000, 1601 Fifth Avenue, Seattle, Washington, 98101, United States.  Chairperson: Kelley, David B. |

**Study HZA106827: 64 Centres**

| **HZA106827**  **Hospital/ Institution and Address** | **HZA106827**  **IEC/IRB Committee Chair and Name of Committee** |
| --- | --- |
| **Germany** |  |
| Institut Medars GmbH i.G., Kaiserdamm 9, Berlin, 14057 Germany. | Ethik-Kommission der Ladesarztekammer Hessen,  Im Vogelgesang 3, Frankfurt, 60488, Germany.  Chairperson: Harder,S |
| Colonnaden 72, Hamburg, 20354 Germany. | Ethik-Kommission der Ladesarztekammer Hessen,  Im Vogelgesang 3, Frankfurt, 60488, Germany.  Chairperson: Harder,S. |
| Grunewaldstr. 6, Berlin, 15165, Germany. | Ethik-Kommission der Ladesarztekammer Hessen,  Im Vogelgesang 3, Frankfurt, 60488, Germany.  Chaiperson: Harder,S |
| Praxis Dr. med. Grit Lammert-Huenger, Lehnitzstrasse 21a,  Oranienburg, Brandenburg,  16515 Germany. | Ethik-Kommission der Ladesarztekammer Hessen,  Im Vogelgesang 3, Frankfurt, 60488, Germany Chairperson: Harder,S |
| KFB- Klinsche Forschung Berlin, Ansbacher Str. 17-19, Berlin, 10787, Germany. | Ethik-Kommission der Ladesarztekammer Hessen,  Im Vogelgesang 3, Frankfurt, 60488, Germany.  Chairperson: Harder,S |
| Facharztzentrum in den Main-Kinzig Kliniken Gelnhausen,  Haus E, Ebene 10,  Herzbachweg 14, Gelnhausen, 63571, Germany. | Ethik-Kommission der Ladesarztekammer Hessen,  Im Vogelgesang 3, Frankfurt, 60488, Germany.  Chairperson: Harder,S |
| Collinistr. 11, Mannheim, Baden-Wuerttemberg 68161, Germany. | Ethik-Kommission der Ladesarztekammer Hessen,  Im Vogelgesang 3, Frankfurt, 60488, Germany  Chairperson: Harder,S |
| Marburger Str. 12-13, Berlin, 10789, Germany. | Ethik-Kommission der Ladesarztekammer Hessen,  Im Vogelgesang 3, Frankfurt, 60488, Germany  Chairperson: Graff, Jochen |
| Medaimun GmbH,  Kennedyallee 97a, Frankfurt am Main, 60596, Germany. | Ethik-Kommission der Ladesarztekammer Hessen,  Im Vogelgesang 3, Frankfurt, 60488, Germany  Chairperson: : Harder,S |
| **Japan** |  |
| Hiroshima Allergy&Respiratory Clinic  6F, Daiichi Teraoka Building  9-28, 1-chome, Hikarimachi, Higashi-ku, Hiroshima-shi  Hiroshima  732-0052, Japan. | Hiroshima Allergy&Respiratory Clinic,  6F, Daiichi Teraoka Building,  9-28, 1-chome, Hikarimachi, Higashi-ku, Hiroshima-shi, Hiroshima, 732-0052, Japan.  Chairperson: Maeda, Akihiro. |
| Okusawa Hospital & Clinics  2-11-11, Okusawa, Setagaya-ku, Tokyo 158-0083, Japan. | Medical Corporation Shinkenkai Suzuki Internal Medicine Cardiovascular Medicine,  202, Maison de Statue,  1-39-5, Sangenjaya, Setagaya-ku, Tokyo, 154-0024, Japan.  Chairperson: Nakajima, Osamu |
| Machida Municipal Hospital  2-15-41, Asahimachi, Machida-city, Tokyo 194-0023, Japan. | Machida Municipal Hospital,  2-15-41, Asahimachi, Machida-city, Tokyo, 194-0023, Japan.  Chairperson: Mizuno, Ryoji |
| Kawai Chest Clinic, 43-3, Koyamakitaohnocho, Kita-ku, Kyoto-city, Kyoto 603-8161, Japan. | Kawai Chest Clinic,  43-3, Koyamakitaohnocho, Kita-ku, Kyoto-city, Kyoto, 603-8161, Japan.  Chairperson: Sawaoka, Heiwa |
| Koizumi Clinic of Respiratory and Internal Medicine,  1-41, Minami 1-jo Nishi, 27-chome, Chuo-ku, Sapporo-city  Hokkaido, 064-0801, Japan. | Koizumi Clinic of Respiratory and Internal Medicine,  1-41, Minami 1-jo Nishi, 27-chome, Chuo-ku, Sapporo-city, Hokkaido, 064-0801, Japan.  Chairperson: Mitani, Ikuo |
| Medical Corporation HSR Nakamura Clinic, 4-2-1, Iso, Urasoe-city  Okinawa 901-2132, Japan | Sakaide City Hospital,  6-43, Bunkyo-cho 1-chome, Sakaide-city, Kagawa, 762-0031, Japan. Chairperson: Taoka, Teruhisa |
| Department of Internal Medicine, Medical Corporation HSR Nakamura Clinic 4-2-1, Iso, Urasoe-city  Okinawa 901-2132, Japan. | Kobari General Hospital,  29-1, Yokochi, Noda-shi, Chiba, 278-8501, Japan.  Chairperson: Ninomiya, Hiroki |
| Ishikawa Prefectural Central Hospital, 2-1, Kuratsukihigashi, Kanazawa-city, Ishikawa,  920-8530, Japan. | Ishikawa Prefectural Central Hospital,  2-1, Kuratsukihigashi, Kanazawa-city, Ishikawa, 920-8530, Japan.  Chairperson: Shimamura, Masayoshi |
| National Hospital Organization Fukuoka National Hospital,4-39-1, Yakatabaru, Minami-ku, Fukuoka-city, Fukuoka, 811-1394, Japan. | National Hospital Organization Fukuoka National Hospital,  4-39-1, Yakatabaru, Minami-ku, Fukuoka-city, Fukuoka, 811-1394, Japan. Chairperson: Odajima, Hiroshi |
| Shinada Respiratory and Cardiovascular Clinic, Hashimoto Medical Bldg. 2F, 3-14-1, Hashimoto, Midori-Ku, Sagamihara-city, Kanagawa, 252-0143, Japan. | Arima Kondo Memorial Medical Foundation Tomisaka Clinic,  Institutional Review Board, 2-5-7, Koishikawa, Bunkyo-ku, Tokyo, 112-0002, Japan. Chairperson: Tsushima, Keizo |
| Tanaka Clinic, 1F, Tanaka Kosan Building 1-192, Miyake, Shikama-ku, Himeji-city, Hyogo,  672-8048, Japan. | Nose Clinic,  1-5-10, Nishitenma, Kita-ku, Osaka-shi, Osaka, 530-0047, Japan.  Chairperson: Hoshi, Mitsuru |
| Sekino Hospital, 3-28-3, Ikebukuro, Toshima-ku, Tokyo, Japan. | Sekino Hospital,  3-28-3, Ikebukuro, Toshima-ku, Tokyo, 171-0014, Japan.  Chairperson: Akiyama, Kenji |
| **Poland** |  |
| Centrum Medyczne Lucyna Andrzej Dymek, Warynskiego 4, Zawadzkie, 47-120, Poland. | Komisja Bioetyczna przy DIL,  Al. J. Matejki 6, Wroclaw, 50-333, Poland  Chairperson: Zynda, Lech |
| DOBROSTAN Gabinety Lekarskie, Slezna 27, Wroclaw 53-301, Poland. | Komisja Bioetyczna przy DIL,  Al. J. Matejki 6, Wroclaw, 50-333, Poland  Chairperson: Zynda, Lech |
| Specjalistyczna Przychodnia Lekarska ALEGORM-MED,  UL. PCK 26, Tarnow, 33-100, Poland. | Komisja Bioetyczna przy DIL,  Al. J. Matejki 6, Wroclaw, 50-333, Poland  Chairperson: Zynda, Lech |
| **Romania** |  |
| Spitalul Judetean de Urgenta Pitesti, Ale. Spitalului nr 36  Pitesti 110084, Romania. | Comisia Naţională de Etică,  Str. Av. Sănătescu 48, Bucureşti, 11478, Romania.  Chairperson: Dumitrescu, Sava |
| Spitalului Clinic de Pneumoftiziologie “Leon Daniello” Cluj Str. B. P. Hasdeu nr 6, cluj napoca 400371, Romania. | Comisia Naţională de Etică,  Str. Av. Sănătescu 48, Bucureşti, 11478, Romania.  Chairperson: Dumitrescu, Sava |
| Spitalul Clinic Judetean de Urgenta Deva, Str. 22 Decembrie nr. 58, Deva, 330084, Romania. | Comisia Naţională de Etică,  Str. Av. Sănătescu 48, Bucureşti, 11478, Romania.  Chairperson: Dumitrescu, Sava |
| Centrul Medical Valahia SRL,  Str. Mihai Bravu nr 2, Ploiesti 100550, Romania | Comisia Naţională de Etică,  Str. Av. Sănătescu 48, Bucureşti, 11478, Romania.  Chairperson: Dumitrescu, Sava |
| Romclinic SRL, Str. Ardeleni nr. 3A, Bucharest, 20674, Romania. | Comisia Naţională de Etică,  Str. Av. Sănătescu 48, Bucureşti, 11478, Romania.  Chairperson: Dumitrescu, Sava |
| Spitalul Judetean de Urgenta "Sf. Ioan cel Nou"  Strada 1 Decembrie 1918 nr. 21  Suceava 720284, Romania. | Comisia Naţională de Etică,  Str. Av. Sănătescu 48, Bucureşti, 11478, Romania.  Chairperson: Dumitrescu, Sava |
| Spitalul Clinic Judetean de Urgenta Craiova  Str Tabaci nr. 1, Craiova 200642, Romania. | Comisia Naţională de Etică,  Str. Av. Sănătescu 48, Bucureşti, 11478, Romania.  Chairperson: Dumitrescu, Sava |
| Spitalul Clinic de boli infectioase si pneumoftiziologie "Dr Victor Babes"  Str. Gheorghe Adam nr 13  Timisoara 300310, Romani | Comisia Naţională de Etică,  Str. Av. Sănătescu 48, Bucureşti, 11478, Romania.  Chairperson: Dumitrescu, Sava |
| **Ukraine** |  |
| Kharkiv City Hospital #13, Department of Pulmonology 137 Gagarina Str, Kharkiv 61035, Ukraine. | Central Ethics Committee of Ministry of Health of Ukraine,  5-Narodnogo opolchennia Str, Kyiv, 3680, Ukraine. Chairperson: Kornatskyi, Vasyl |
| City Clinical Hospital #7  Department of Allergology  26, Shmidta Str., Dnipropetrovsk  49006, Ukraine. | Central Ethics Committee of Ministry of Health of Ukraine,  5-Narodnogo opolchennia Str, Kyiv, 3680, Ukraine.  Chairperson: Kornatskyi, Vasyl |
| Institute of Phthisiatry and Pulmonology,  Dep. of technologies of treatment of NSPD, 10, Amosova str., Kyiv, 03680, Ukraine. | Central Ethics Committee of Ministry of Health of Ukraine,  5-Narodnogo opolchennia Str, Kyiv, 3680, Ukraine. Chairperson: Kornatskyi, Vasyl |
| Institute of Phthisiatry and Pulmonology,  Department of Pulmonology, 10, Amosova Str, Kiev, 3680, Ukraine. | Central Ethics Committee of Ministry of Health of Ukraine,  5-Narodnogo opolchennia Str, Kyiv, 3680, Ukraine. Chairperson: Kornatskyi, Vasyl |
| Institute of Phthisiatry and Pulmonology,  Dept of Clinical Functional Diagnostics, 10, Amosova Str, Kiev, 3680, Ukraine. | Central Ethics Committee of Ministry of Health of Ukraine,  5-Narodnogo opolchennia Str, Kyiv, 3680, Ukraine. Chairperson: Kornatskyi, Vasyl |
| Institute of Phthisiatry and Pulmonology,  Department of the Diagnostics, Therapy and Clinica, 10, Amosova Str, Kiev, 3680, Ukraine. | Central Ethics Committee of Ministry of Health of Ukraine,  5-Narodnogo opolchennia Str, Kyiv, 3680, Ukraine. Chairperson: Kornatskyi, Vasyl |
| Zaporizhia City multi specialty children’s hospital # 5, Department of allergology 28-A, Novgorodskaya Str., Zaporizhia 69076, Ukraine. | Central Ethics Committee of Ministry of Health of Ukraine,  5-Narodnogo opolchennia Str, Kyiv, 3680, Ukraine.  Chairperson: Kornatskyi, Vasyl |
| Regional Center of Phthisiology and Pulmonology, Department of Pulmonology, 17 I.Franka Str., Ivano-Frankivsk 76018, Ukraine. | Central Ethics Committee of Ministry of Health of Ukraine,  5-Narodnogo opolchennia Str, Kyiv, 3680, Ukraine. Chairperson: Kornatskyi, Vasyl |
| City Clinical Hospital # 6,  Department of therapy with pulmonological wards, 13 Batumska street, Dnipropetrovsk, 49051, Ukraine. | Central Ethics Committee of Ministry of Health of Ukraine,  5-Narodnogo opolchennia Str, Kyiv, 3680, Ukraine. Chairperson: Kornatskyi, Vasyl |
| Kyiv City Clinical Hospital # 8  Allergological department # 1  Kondratyuka Str. 8 Kyiv 4201, Ukraine. | Central Ethics Committee of Ministry of Health of Ukraine,  5-Narodnogo opolchennia Str, Kyiv, 3680, Ukraine.  Chairperson: Kornatskyi, Vasyl |
| Simferopol Railroad Hospital  Therapy and Allergology Department  142, Kyivska str., Simferopol 95043, Ukraine. | Central Ethics Committee of Ministry of Health of Ukraine,  5-Narodnogo opolchennia Str, Kyiv, 3680, Ukraine.  Chairperson: Kornatskyi, Vasyl |
| **United States** |  |
| Baker Allergy, Asthma and Dermatology Research Center, LLC 3975 SW Mercantile Drive Suite 165 Lake Oswego, OR 97035, United States. | Quorum Review IRB 1601 Fifth Avenue Suite 1000 Seattle, WA 98101.  Chairperson: Kelley, David |
| Bernstein Clinical Research Center, 8444 Winton Road, Cincinnati, Ohio 45231, United States. | Quorum Review IRB 1601 Fifth Avenue Suite 1000 Seattle, WA 98101.  Chairperson: Kelley, David |
| IPS Research Company 1111 N.Lee, Ste. 400 Oklahoma City, OK 73103, United States. | Quorum Review IRB 1601 Fifth Avenue Suite 1000 Seattle, WA 98101.  Chairperson: Kelley, David |
| Clinical Research Institute of Southern Oregon, PC 3860 Crater Lake Avenue, Suite B Medford OR 97504, United States. | Quorum Review IRB 1601 Fifth Avenue Suite 1000 Seattle, WA 98101.  Chairperson: Kelley, David |
| Quality Control Research, Inc 568 N Sunrise Avenue, Suite 180 Roseville, CA 65661, United States. | Quorum Review IRB 1601 Fifth Avenue Suite 1000 Seattle, WA 98101.  Chairperson: Kelley, David |
| Huntington Beach Internal Medicine Group, Inc.,  Suite 111 1501 Superior Ave,  Newport Beach, California 92663, United States. | Quorum Review IRB 1601 Fifth Avenue Suite 1000 Seattle, WA 98101.  Chairperson: Kelley, David |
| Asthma And Allergy Center of Chicago, SC 7420 Central, Suite 2020 River Forest, Tllinois 60305, United States. | Quorum Review IRB 1601 Fifth Avenue Suite 1000 Seattle, WA 98101.  Chairperson: Kelley, David |
| Allergy & Asthma Care Center of Southern California 3816 Wooddrugg Ave, #209 Long Beach, California 90808, United States. | Quorum Review IRB 1601 Fifth Avenue Suite 1000 Seattle, WA 98101.  Chairperson: Kelley, David |
| Sneeze, Wheeze, & Itch Associates, LLC 2010 Jacobssen Drive Normal, Illinois 61761, United States. | Quorum Review IRB 1601 Fifth Avenue Suite 1000 Seattle, WA 98101.  Chairperson: Kelley, David |
| Allergy Associates Research Center Suite 310 545 N.E. 47th Avenue, Portland Oregon 97213, United States. | Quorum Review IRB 1601 Fifth Avenue Suite 1000 Seattle, WA 98101.  Chairperson: Kelley, David |
| Pulmonary Disease and Critical Care Asscoiates, P.A.  10710.Charter Drive Suiite 310 Columbia, Maryland 21044, United States. | Quorum Review IRB 1601 Fifth Avenue Suite 1000 Seattle, WA 98101.  Chairperson: Kelley, David |
| Florida Center For Allergy and Asthma Research 9035 Sunset Drive  Miami, Florida 33173, United States. | Quorum Review IRB 1601 Fifth Avenue Suite 1000 Seattle, WA 98101.  Chairperson: Kelley, David |
| Integrated Research Group, Inc. 4646 Brockton Avenue Suites 101/201/202/302, Riverside California 92506, United States. | Quorum Review IRB 1601 Fifth Avenue Suite 1000 Seattle, WA 98101.  Chairperson: Kelley, David |
| Allergy Associates Medical Group, Inc., 6386 Alvarado Ct. Ste. 210 San Diego, California 92120, United States. | Quorum Review IRB 1601 Fifth Avenue Suite 1000 Seattle, WA 98101.  Chairperson: Kelley, David |
| Pediatric Care Medical Group, Inc. 17822 Beach Blvd., Suite 278 Huntington Beach California 92647, United States. | Quorum Review IRB 1601 Fifth Avenue Suite 1000 Seattle, WA 98101.  Chairperson: Kelley, David |
| Respiratory Diseases of Children and Adolescents Suite 330 Baptist Medical Plaza Bldg D 3366 NW Expressway Oklahoma City Oklahoma 73112, United States. | Quorum Review IRB 1601 Fifth Avenue Suite 1000 Seattle, WA 98101.  Chairperson: Kelley, David |
| SunRise Clinical Research 5985 Florence Ave #N Bell Gardens California 90201, United States. | Quorum Review IRB 1601 Fifth Avenue Suite 1000 Seattle, WA 98101.  Chairperson: Kelley, David |
| Carolina Research, 2227 Saint Mathews Road, Orangeburg, South Carolina 29118, United States. | Quorum Review IRB 1601 Fifth Avenue Suite 1000 Seattle, WA 98101.  Chairperson: Kelley, David |
| Clinical Research Of The Ozarks, Inc., 509 East 10th Street Rolla, Missouri 6540, United States. | Quorum Review IRB 1601 Fifth Avenue Suite 1000 Seattle, WA 98101.  Chairperson: Kelley, David |
| Southern California Institute for Respiratory Diseases, Inc., 8635 West Third Street Suite 965W, Los Angeles California 90048, United States | Quorum Review IRB 1601 Fifth Avenue Suite 1000 Seattle, WA 98101.  Chairperson: Kelley, David |

**Study HZA106837: 167 Centres**

| **HZA106837**  **Hospital/ Institution and Address** | **HZA106837**  **IEC/IRB Committee Chair and Name of Committee** |
| --- | --- |
| **Argentina** |  |
| Centro Médico Dra De Salvo, Av. Cabildo 1548 1°A, Ciudad Autónoma de Buenos Aires, C1426ABO, Argentina. | Comite de Etica Independiente en Investigacion Clinica Dr Carlos A Barclay Larrea 1381 3°A, C1117ABK, Ciudad Autonoma de Buenos Aires, Argentina.  Chairperson: Barclay, Carlos A  Comite Independiente de Etica para Ensayos en Farmacologia Clinica Av, Cabildo 1548 1°A, C1426ABP, Ciudad Autonoma de Buenos Aires, Argentina.  Chairperson: De Girolamo, Silvia |
| INSARES, José Rondeau 335, M5500CCG, Mendoza Argentina. | Comite de Etica Independiente en Investigacion Clinica, "Dr. Carlos A. Barclay", Larrea 1381 3° A, C1117ABK Ciudad Autonoma de Buenos Aires Argentina.  Chairperson: Barclay, Carlos A  Comite de Docencia e Investigacion, INSARES, Jose Rondeau 335, M5500CCG Mendoza, Argentina.  Chairperson: Agüero, Luis. MD |
| Instituto Cardiovascular de Rosario, Boulevar Oroño 450, S2000DSR Rosario, Santa Fe Argentina. | Comite de Etica Independiente en Investigacion Clinica, "Dr. Carlos A. Barclay", Larrea 1381 3° A, C1117ABK Ciudad Autonoma de Buenos Aires Argentina.  Chairperson: Barclay, Carlos A  Comite de Docencia e Investigacion, Instituto Cardiovascular de Rosario, Boulevar Oroño 450, S2000DSR Rosario, Santa Fe. Argentina.  Chairperson: Zapata, Gerardo. MD |
| Centro Privado de Medicina Respiratoria, Alsina 549, E3100BHK, Parana, Entre Ríos, Argentina. | Comite de Etica Independiente en Investigacion Clinica, "Dr. Carlos A. Barclay", Larrea 1381 3° A, C1117ABK Ciudad Autonoma de Buenos Aires Argentina.  Chairperson: Barclay, Carlos A  Comite de Docencia, Centro Privado de Medicina Respiratoria, Alsina 549, E3100BHK, Parana, Entre Ríos, Argentina.  Chairperson: Dagnino, Diego |
| Fundacion CIDEA, Paraguay 2035, 3er Cuerpo - 2do Subsuelo, C1121ABE, Ciudad Autónoma de Buenos Aires, Argentina. | Comite de Etica Independiente en Investigacion Clinica, "Dr. Carlos A. Barclay", Larrea 1381 3° A, C1117ABK Ciudad Autonoma de Buenos Aires Argentina.  Chairperson: Barclay, Carlos A  Comite de Docencia e Investigaciön, Fundacion CIDEA, Billinghurst 1677 3º Piso, C1425DTG, Ciudad Autónoma de Buenos Aires, Argentina.  Chairperson: Diaz Couselo, Fernando. MD |
| Centro de Enfermedades Respiratorias e Investigaciones, Av. Pedro Goyena 551, C1424BSF. Ciudad Autonoma de Buenos Aires, Argentina. | Comite de Etica Independiente en Investigacion Clinica, "Dr. Carlos A. Barclay", Larrea 1381 3° A, C1117ABK Ciudad Autonoma de Buenos Aires Argentina.  Chairperson: Barclay, Carlos A  CIRE- Comite Independiente de Revision y Etica, Av. Pedro Goyena 551, C1424BSF, Ciudad Autonoma de Buenos Aires, Argentina.  Chairperson: Rey,Rodolfo |
| Investigaciones en, Patologias Respiratorias, Balcarce 874, 4000 Tucuman, Argentina. | Comite de Etica Independiente en Investigacion Clinica, "Dr. Carlos A. Barclay", Larrea 1381 3° A, C1117ABK Ciudad Autonoma de Buenos Aires Argentina.  Chairperson: Barclay, Carlos A  Comite de Docencia e Investigacion de Investigaciones en, Patologias Respiratorias, Balcarce 874, 4000 Tucuman, Argentina.  Chairperson: Gerez, Liliana. MD |
| Instituto InAER, Arenales 3146 - Piso 1º Depto B, C1425BEN Ciudad Autonoma de Buenos Aires, Argentina. | Comite de Etica Independiente en Investigacion Clinica, "Dr. Carlos A. Barclay", Larrea 1381 3° A, C1117ABK Ciudad Autonoma de Buenos Aires, Argentina.  Chairperson: Barclay, Carlos A  Comite de Docencia e Investigacion Instituto InAER, Arenales 3146 - Piso 1º B, C1425BEN Ciudad Autonoma de Buenos Aires, Argentina.  Chairperson: Fantin, Sara. MD |
| **Australia** |  |
| The Canberra Hospital, Clinical Trials Unit, Level 2, Building 1, Yamba Drive, Garran, ACT 2065 Australia. | ACT Health Human Research,Ethics Committee,  11 Moore Street, Canberra City, ACT 2601, Australia.  Chairperson:Biggs,John |
| Respiratory Clinica Trials Pty--Ltd, C/- Clinical Trials Centre, 120 Kensington Road, Toorak Gardens, South Australia, 5065, Australia. | Bellberry Human Research Ethics Committee, Bellberry Limited, 229 Greenhill Road Dulwich - South Australia 5065 Australia.  Chairperson : Stoffel, Brian |
| Austrials Pty Ltd, 19 lang Parade, Auchenflower – Queensland 4066, Australia. | Bellberry Human Research Ethics Committee, Bellberry Limited, 229 Greenhill Road Dulwich – South Australia 5065 Australia.  Chairperson : Stoffel, Brian |
| Monash Mediacl Centre, 246 Clayton Road, Clayton- Victoria 3168 Australia. | Southern Health Human Research Ethics Committee, Research Directorate Souther Health, 246 Clayton Road, Clayton- Victoria 3168.  Chairperson: Munro,Judith |
| **Germany** |  |
| IFG Institute fuer Gesundheitsfoerderung GmbH, Otto-Nuschke-Strasse 2, 15562, Ruedersdorf, Germany. | Ethik-Kommission der Bayerischen Landesaerztekamer  Mühlbaurstraße 16, 81677 München, Germany.  Chairperson : Hasford, Joerg |
| Institut Dr. Benedix und Dr. Schnorr für Gesundheitsförderung u. Beratungs GmbH Gaeblerstr. 18 13086 Berlin, Germany. | Ethik-Kommission der Bayerischen Landesaerztekamer  Mühlbaurstraße 16, 81677 München, Germany.  Chairperson : Hasford, Joerg |
| Dr. med. Hans-Christian Blum, Hermannstr. 48-52, 44263 Dortmund, Germany. | Ethik-Kommission der Bayerischen Landesaerztekamer  Mühlbaurstraße 16, 81677 München, Germany.  Chairperson : Hasford, Joerg |
| IKF Pneumologie GmbH & Co. KG, Institut für klinische Forschung, Strsemannallee 3, 60596, Frankfurt am Main Germany. | Ethik-Kommission der Bayerischen Landesaerztekamer  Mühlbaurstraße 16, 81677 München, Germany.  Chairperson : Hasford, Joerg |
| Dr.med. Karl-Heinz Franz, MD, Theodor-Heuss-Str. 2, 58452 Witten, Germany. | Ethik-Kommission der Bayerischen Landesaerztekamer  Mühlbaurstraße 16, 81677 München, Germany.  Chairperson : Hasford, Joerg |
| Dr. Gesine Groth, Erdkampsweg 49, 22335 Hamburg, Germany. | Ethik-Kommission der Bayerischen Landesaerztekamer  Mühlbaurstraße 16, 81677 München, Germany.  Chairperson : Hasford, Joerg |
| Susanne Holtz. MD, Puehlstrasse. 37, 55624 Rhaunen, Germany. | Ethik-Kommission der Bayerischen Landesaerztekamer  Mühlbaurstraße 16, 81677 München, Germany.  Chairperson : Hasford, Joerg |
| Dr. med. Peter, Hofbauer, Bahnhofstr. 3-9, 69469, Weinheim, Germany. | Ethik-Kommission der Bayerischen Landesaerztekamer  Mühlbaurstraße 16, 81677 München, Germany.  Chairperson : Hasford, Joerg |
| Pulmologisches Institut Bamberg in Potsdam GmbH, Hebbelstr. 6, 14469 Potsdam, Germany. | Ethik-Kommission der Bayerischen Landesaerztekamer  Mühlbaurstraße 16, 81677 München, Germany.  Chairperson : Hasford, Joerg |
| PD Dr. med. Gerhard Hoheisel, MD, August-Bebel-Str. 69, 04275, Leipzig, Germany. | Ethik-Kommission der Bayerischen Landesaerztekamer  Mühlbaurstraße 16, 81677 München, Germany.  Chairperson : Hasford, Joerg |
| Dr. med. Martin Hoster, MD, Kurt-Schumacher-Platz 4, Bochum, Germany, 44787. | Ethik-Kommission der Bayerischen Landesaerztekamer  Mühlbaurstraße 16, 81677 München, Germany.  Chairperson : Hasford, Joerg |
| Dr. med. Thomas Jung, Ditzenbacher Str. 33, 73326 Deggingen, Germany. | Ethik-Kommission der Bayerischen Landesaerztekamer  Mühlbaurstraße 16, 81677 München, Germany.  Chairperson : Hasford, Joerg |
| Dr. med. Frank Kaessner, MD, MECS cottbus GmbH, Thiemstr. 124, 3050 Cottbus, Germany. | Ethik-Kommission der Bayerischen Landesaerztekamer  Mühlbaurstraße 16, 81677 München, Germany.  Chairperson : Hasford, Joerg |
| Dr. med. Claus Keller, Usinger Strasse 5, 60389 Frankfurt, Germany. | Ethik-Kommission der Bayerischen Landesaerztekamer  Mühlbaurstraße 16, 81677 München, Germany.  Chairperson : Hasford, Joerg |
| Dr. med. Stephanie Korn, MD, Klinische Forschung Pneumologie, III Medizinische Klinik Universitätsmedizin Langenbeckstr. 1, 55131 Mainz, Germany. | Ethik-Kommission der Bayerischen Landesaerztekamer  Mühlbaurstraße 16, 81677 München, Germany.  Chairperson : Hasford, Joerg |
| Dr. med. Karl-Heinz Krause, MD, medicoKIT, Institut für klinische Arzneimitlelprüfungen, Brückenstr. 42, 47574 Goch, Germany. | Ethik-Kommission der Bayerischen Landesaerztekamer  Mühlbaurstraße 16, 81677 München, Germany.  Chairperson : Hasford, Joerg |
| Dr. med. Roland Schätzl, MD Altenbanzer Weg 3, 96269 Großheirath, Germany. | Ethik-Kommission der Bayerischen Landesaerztekamer  Mühlbaurstraße 16, 81677 München, Germany.  Chairperson : Hasford, Joerg |
| Dr. med. Olaf Schmidt, MD, Emil-Schueller-Str. 29, 56068 Koblenz, Germany. | Ethik-Kommission der Bayerischen Landesaerztekamer  Mühlbaurstraße 16, 81677 München, Germany.  Chairperson : Hasford, Joerg |
| Dr. med, Tibor Schmoller, MD, Dorotheenstr. 174, 22299 Hamburg, Germany. | Ethik-Kommission der Bayerischen Landesaerztekamer  Mühlbaurstraße 16, 81677 München, Germany.  Chairperson : Hasford, Joerg |
| Dr. med. Wolfgang Schuermann, MD, Bahnhofstr. 30, 35037 Marburg, Germany. | Ethik-Kommission der Bayerischen Landesaerztekamer  Mühlbaurstraße 16, 81677 München, Germany.  Chairperson : Hasford, Joerg |
| Dr. med. Gerhard, Stuchlik, Stadtplatz 43, 94474 Vilshofen, Germany. | Ethik-Kommission der Bayerischen Landesaerztekamer  Mühlbaurstraße 16, 81677 München, Germany.  Chairperson : Hasford, Joerg |
| Dr. med. Bernd-Georg Truemper, MD, Neuwerkstrasse 51, 99084 Erfurt, Germany. | Ethik-Kommission der Bayerischen Landesaerztekamer  Mühlbaurstraße 16, 81677 München, Germany.  Chairperson : Hasford, Joerg |
| Dr. med. Weber, Unterstr. 75, 45359 Essen, Germany. | Ethik-Kommission der Bayerischen Landesaerztekamer  Mühlbaurstraße 16, 81677 München, Germany.  Chairperson : Hasford, Joerg |
| **Japan** |  |
| Asamoto Naika Iin, Haimuwisutaria Bldg. 1F, 1 Fukakusahotta-cho, Fushimi-ku, Kyoto-city, Kyoto, 612-0026, Japan. | Asamoto Naika Iin Institutional Review Board,  Haimuwisutaria Bldg. 1F, 1 Fukakusahotta-cho,  Fushimi-ku, Kyoto-city, Kyoto, 612-0026, Japan.  Chairperson : Hioki, Shinichiro |
| Nagata Hospital, 523-1, Shimomiyanaga-machi, Yanagawa-shi, Fukuoka, 832-0059, Japan. | Nagata Hospital Institutional Review Board, 523-1, Shimomiyanaga-machi, Yanagawa-shi,  Fukuoka, 832-0059, Japan.  Chairperson:Nagata, Eisuke |
| Koizumi Clinic of Respiratory and Internal Medicine, 1-41, Minami 1-jo Nishi, 27-chome, Chuo-ku, Sapporo-city, Hokkaido, 064-0801, Japan. | Koizumi Clinic of Respiratory and Internal Medicine Institutional Review Board, 1-41, Minami 1-jo Nishi, 27-chome, Chuo-ku, Sapporo-city, Hokkaido, 064-0801, Japan.  Chairperson: Mitani, Ikuo |
| Okano Clinic, 7-2, Akayamahoncho, Koshigaya-shi, Saitama, 343-0808, Japan. | Kousei Medical Clinic Institutional Review Board, 5-2-7, Tachibana, Sumida-ku, Tokyo, 131-0043, Japan.  Chairperson:Hirano, Kei |
| Oki Clinic, 43-6, Takanodai, Kodaira-city, Tokyo, 187-0024, Japan. | Institutional Review Board of Sekino Hospital, 3-28-3 Ikebukuro, Toshima-ku, Tokyo, 171-0014, Japan.  Chairperson: Akiyama, Kenji |
| Medical Corp. Junyo-kai, Musashino Polyclinic, Sanfuku Bldg. 3,4F, 8-30, Motomachi, 1-chome, Kiyose-shi, Tokyo, 204-0021, Japan. | Medical Corp. Jozenkai Shinagawa East One Medical Clinic Institutional Review Board, Shinagawa east one tower 3F, 2-16-1, Konan Minato-ku, Tokyo, 108-0075, Japan.  Chairperson: Sakai, Hideaki |
| Shinada Respiratory and Cardiovascular Clinic, Hashimoto Medical Bldg. 2F, 3-14-1, Hashimoto, Midori-ku, Sagamihara-city, Kanagawa, 252-0143, Japan. | Arima Kondo Memorial Medical Foundation Tomisaka Clinic Institutional Review Board, 2-5-7 Koishikawa, Bunkyo-ku, Tokyo, 112-0002, Japan.  Chairperson: Tsushima, Keizo. |
| Dynamedical Nezu Clinic, Dyna-city Bunkyo Nezu Bldg. 1F, 14-9, Nezu 2-chome, Bunkyo-ku, Tokyo, 113-0031, Japan. | Medical Corp. Jozenkai Shinagawa East One Medical Clinic Institutional Review Board, Shinagawa east one tower 3F, 2-16-1, Konan Minato-ku, Tokyo,108-0075, Japan.  Chairperson: Sakai, Hideaki |
| Yuizen Clinic, NISHI Bldg. 2F, 25-24, Fujimi-cho, 1-chome, Tachikawa-shi, Tokyo, 190-0013, Japan. | Medical Corp. Jozenkai Shinagawa East One Medical Clinic Institutional Review Board, Shinagawa east one tower 3F, 2-16-1, Konan Minato-ku, Tokyo, 108-0075, Japan.  Chairperson: Sakai, Hideaki |
| Kamoike Seikyo Clinic, 5-8, Kamoike-Shinmachi, Kagoshima-city, Kagoshima, 890-0064, Japan. | General Hospital Kagoshima Seikyo Hospital Institutional Review Board, 5-20-10, Taniyamachuo, Kagoshima-city, Kagoshima, 891-0141, Japan.  Chairperson: Morishita, Shigemi |
| K-YOU HEALTH CARE Co. KIRIGAOKA TSUDA HOSPITAL, 3-9-20, Kirigaoka, Kokurakita-ku, Kitakyushu-city, Fukuoka, 802-0052, Japan. | Umezu Clinic Institutional Review Board,  2-6-12 Haruda, Chikushino-city, Fukuoka, 818-0024, Japan.  Chairperson : Inou, Tetsuji |
| Yasuda Clinic, 53, Kuzeoyabu-cho, Minami-ku, Kyoto-city, Kyoto, 601-8206, Japan. | Yasuda Clinic Institutional Review Board, 53, Kuzeoyabu-cho, Minami-ku, Kyoto-city, Kyoto, 601-8206, Japan.  Chairperson : Nakamura, Yoshio |
| **Mexico** |  |
| Hospital del Niño de Alta Especialidad, Centro de Asma Méndez 2832 Colonia Tamulté, Villahermosa, Tabasco, Mexico, CP 86100. | Comite de Etica del Hospital del Niño de Alta Especialidad, Méndez 2832 Colonia Tamulté, Villahermosa, Tabasco, Mexico, CP 86100.  Chairperson: Huerta, Hugo U  Comite Bioetico para la Investigacion Clinica, Puebla No.422-4, Col. Roma, Mexico DF, Mexico, CP 06700.  Chairperson: Savariego, Celia O |
| Torre Italis, Avenida,Copérnico 3817 consultorio 14 esquina Sagitario Colonia Arboledas, Zapopan,Jalisco, Mexico, C.P 45040. | Comite de Bioetica del Instituto IMER de Occidente, Av. Pablo Neruda No. 3148 esquina Victoria, Colonia Providencia, Guadalajara, Jalisco, Mexico CP 44639.  Chairperson: Casillas-Santana, Juan Manuel. MD |
| Instituto Jalisciense de, Investigación Clinica, S.A de C.V. , Penitenciaria 20, Guadalajara, Jalisco, Mexico, CP 44100. | Comite de Etica del Instituto Jalisciense de Investigacion Clinica. S.A. de C.V.  Penitenciaria 20, Guadalajara, Jalisco, Mexico, CP.44100  Chairperson: Avelar Briblesca, Gabriela. MD |
| Hospital Angeles Lindavista, Río Bamba 639 Col. Magdalena de las Salinas, Mexico, DF Mexico CP 07760. | Comite Bioetico para la Investigacion Clinica S.C, Puebla No. 422-4, Col. Roma, Mexico, DF Mexico CP 06700.  Chairperson: Savariego, Celia Ovadía |
| Instituto Nacional de Pediatria, Insurgentes Sur 3700-C Col. Insurgentes Cuicuilco, Mexico DF 04530. | Comite de Etica del lnstituto NAcional de Pediatria  Insurgentes Sur 37OO·C Col. Insurgentes Culculco  CP 01020 Mexico, DF  Chairperson: Yamazaki Nakashimada, Marco Antonio. MD |
| Unidad de Investigación Clínica en Medicina S.C., Av. La Clínica 2520 Int. 520, 522 y 524. Col. Sertoma, Monterrey NL, Mexico CP 64718. | Comite de Etica e Investigación de la Unidad de Investigacion Clínica en Medicina, Av. La Clínica 2520 Int. 520, 522 y 524. Col. Sertoma, Monterrey NL, Mexico CP 64718  Chairperson: Valdovinos-Chavez, Salvador. MD |
| **Philippines** |  |
| Department of Pulmonary Diseases, Veterans Memorial Medical Center, North Avenue, Quezon City 1101, Philippines. | Institutional Review Board, Veterans Memorial Medical Center, North Avenue, Quezon City 1101, Philippines.  Chairperson: Icasiano-Javier, Victoria. MD |
| Mary Mediatrix Medical Center, Rm. 154 Medical Arts Building, J.P. Laurel Highway, Lipa City, Batangas, Philippines 4217. | Institutional Ethics Committee, Mary Mediatrix Medical Center, Rm. 154 Medical Arts Building, J.P. Laurel Highway, Lipa City, Batangas, Philippines 4217.  Chairperson : Comia, Sonia C. MD |
| Center for Respiratory Care, 3rd Floor Spice Building, St. Paul's Hospital, General Luna Street, Iloilo City, Philippines 5000. | Ethics Committee, St. Paul’s Hospital, General Luna Street, Iloilo City, Philippines 5000.  Chairperson : Solomia, Paul FR |
| St. Michael Family Hospital, Poblacion II, Marilao, Bulacan, Philippines 3019. | National Ethics Committee, Room 306 3rd Floor Department of Science and Technology Building, General Santos Avenue, Bicutan, Taguig, Philippines 1631.  Chairperson: Reyes, Marita VT. MD |
| Quirino Memorial Medical Center, Katipunan Road, Project 4, Quezon City1109 Philippines. | Hospital Ethics Committee, Quirino Memorial Medical Center, Katipunan Road, Project 4, Quezon City1109 Philippines.  Chairperson: Reside, Evelyn Victoria E. MD, FPCP, FPCCP |
| **Poland** |  |
| ZOZ, Zespol Poradni Specjalistycznych, Poradnia Alergologiczna, ul. Krakowska 91, 39-200 Debica. | Komisja Bioetyczna, przy Akademii Medycznej w Lublinie, Al. Raclawickie 1, 20-059 Lublin.  Chairperson : Olajossy, Marcin |
| NZOZ Lekarze Specjalisci- J Malolepszy I Partnerzy, ul. Wejherowska 28, 54-239 Wroclow, Poland. | Komisja Bioetyczna, przy Akademii Medycznej w Lublinie, Al. Raclawickie 1, 20-059 Lublin.  Chairperson : Olajossy, Marcin |
| Centrum Alergologii Teresa Hofman ul. Boguslawskiego 16a, 62-023 Poznań, Poland. | Komisja Bioetyczna, przy Akademii Medycznej w Lublinie, Al. Raclawickie 1, 20-059 Lublin.  Chairperson : Olajossy, Marcin |
| Wojewodzka Specjalistyczna Przychodnia Gruzlicy I Chorob Pluc, Poradnia Alergologiczna, ul Jagiellonska 72, 25- 734 Kielce. | Komisja Bioetyczna, przy Akademii Medycznej w Lublinie, Al. Raclawickie 1, 20-059 Lublin.  Chairperson : Olajossy, Marcin |
| Przychodnia Alergologiczno-Pulmonologiczna "Alergopneums", ul. Probostwo 5/1, 20-089 Lublin. | Komisja Bioetyczna, przy Akademii Medycznej w Lublinie, Al. Raclawickie 1, 20-059 Lublin.  Chairperson : Olajossy, Marcin |
| SP ZOZ, Specjalistyczny Zespol Gruzlicy I Chrob Pluc, ul. Rycerska 2, 35-241 Rzeszow. | Komisja Bioetyczna, przy Akademii Medycznej w Lublinie, Al. Raclawickie 1, 20-059 Lublin.  Chairperson : Olajossy, Marcin |
| NZOZ ATOPIA, Al.Slowackiego 39, 31-159 Krakow. | Komisja Bioetyczna, przy Akademii Medycznej w Lublinie, Al. Raclawickie 1, 20-950 Lublin.  Chairperson : Olajossy, Marcin |
| Centrum Medyczne PRO-MED, Poradnia Gruzlicy I Chorob Pluc, ul. Armii Krajowej 64/13, 05-200 Wolomin. | Komisja Bioetyczna, przy Akademii Medycznej w Lublinie, Al. Raclawickie 1, 20-059 Lublin.  Chairperson : Olajossy, Marcin |
| Samodzielny Publiczny Zespol Gruzlicy i Chorob Pluc, ul. Jagiellonska 78, 10-357 Olsztyn. | Komisja Bioetyczna, przy Akademii Medycznej w Lublinie, Al. Raclawickie 1, 20-059 Lublin.  Chairperson : Olajossy, Marcin |
| 10, Wojskowy Szpital Kliniczny z Polikliniką, SP ZOZ, Oddzial Kliniczny Pulmonologiczny, ul. Powstancow, Warszawy 5, 85-681 Bydgoszcz. | Komisja Bioetyczna, przy Akademii Medycznej w Lublinie, Al. Raclawickie 1, 20-059 Lublin.  Chairperson : Olajossy, Marcin |
| Prywatna Przychodnia, Specjalistyczna Alergosan, Poradnia Alergologiczna, ul. Jabloniowa 24 b/c, 75-679, Koszalin. | Komisja Bioetyczna, przy Akademii Medycznej w Lublinie, Al. Raclawickie 1, 20-059 Lublin.  Chairperson : Olajossy, Marcin |
| SNZOZ Alergologia Plus, Osrodek Diagnostyki I Terapii Uczulen, ul Drobnika 49, 60-693 Poznan. | Komisja Bioetyczna, przy Akademii Medycznej w Lublinie, Al. Raclawickie 1, 20-059 Lublin.  Chairperson : Olajossy, Marcin |
| **Romania** |  |
| Spitalul Judetean de Urgenta Pitesti, Ale. Spitalului nr 36, 110084, Pitesti, Romania. | National Drug Agency, 48 Avaitor Sanatescu Street, District 1, postal code 011478, Bucharest, Romania.  Chairperson: Boda, Daniel  National Ethics Committee, 48 Avaitor Sanatescu Street, District 1, postal code 011478, Bucharest, Romania.  Chairperson: Dumitrescu, Sava |
| Spitalul Clinic Municipal Filantropia Craiova, Str. Corneliu Coposu nr. 107, 200341, Craiova, Romania. | National Drug Agency, 48 Avaitor Sanatescu Street, District 1, postal code 011478, Bucharest, Romania.  Chairperson: Boda, Daniel  National Ethics Committee, 48 Avaitor Sanatescu Street, District 1, postal code 011478, Bucharest, Romania.  Chairperson: Dumitrescu, Sava |
| Spitalul Clinic Judetean de Urgenta Deva, Ambulatoriul de Specialitate, Str. 22, Decembrie nr. 58, cod postal 330084, Deva judetul, Hunedoara, Romania. | National Drug Agency, 48 Avaitor Sanatescu Street, District 1, postal code 011478, Bucharest, Romania.  Chairperson: Boda, Daniel  National Ethics Committee, 48 Avaitor Sanatescu Street, District 1, postal code 011478, Bucharest, Romania.  Chairperson: Dumitrescu, Sava |
| Romclinic SRL, Str. Ardeleni nr. 3A, cod postal 020674, Bucharest, Romania. | National Drug Agency, 48 Avaitor Sanatescu Street, District 1, postal code 011478, Bucharest, Romania.  Chairperson: Boda, Daniel  National Ethics Committee, 48 Avaitor Sanatescu Street, District 1, postal code 011478, Bucharest, Romania.  Chairperson: Dumitrescu, Sava |
| Institutul De Pneumoftiziologie Marius Nasta, Sos Viilor nr. 90, 050159 Bucuresti Romania. | National Drug Agency, 48 Avaitor Sanatescu Street, District 1, postal code 011478, Bucharest, Romania.  Chairperson: Boda, Daniel  National Ethics Committee, 48 Avaitor Sanatescu Street, District 1, postal code 011478, Bucharest, Romania.  Chairperson: Dumitrescu, Sava |
| Spitalul clinic de pediatrie Sibiu, Clinica de Pediatrie, str Pompeiu Onofreiu, nr 2-4, cod postal 550166, Sibiu, Romania. | National Drug Agency, 48 Avaitor Sanatescu Street, District 1, postal code 011478, Bucharest, Romania.  Chairperson: Boda, Daniel  National Ethics Committee, 48 Avaitor Sanatescu Street, District 1, postal code 011478, Bucharest, Romania.  Chairperson: Dumitrescu, Sava |
| **Russian Federation** |  |
| Saratov State Medical University, Chair of Clinical Immunology, Proviantskaya, 22, Saratov, 420028, Russian Federation. | Ministry of Healthcare and Social Development of the Russian Federation. Ethics Council, 127994, Moscow, Rahmanovskij pereulok, 3. Russia.  Chairperson: Baybarina, Elena  Ethics Committee under Saratov State Medial University, Bolshaya Sadovaya str. Saratov 137, 410054 Russia.  Chairperson: Voskresenskaya, Olga |
| Kazan Research Institution of Epidemiology and Microbiology of Rospotrebnadzor, 67, Bolshaya Krasnaya ul, Kazan, 420015, Russian Federation. | Ministry of Healthcare and Social Development of the Russian Federation. Ethics Council, 3, Rahmanovskij pereulok, Moscow, 127994, Russian Federation.  Chairperson: Baybarina, Elena  Local Ethic committee of Federal State Institution of Science "Kazon Scientific-Research Institution of Epidemiology and Microbiology of Rospotrebnadzor”, 67, Bolshaya Krasnaya ul, Kazan, 420015, Russian Federation.  Chairperson: Andreev, Sergey |
| Regional Clinical Advisory and Diagnistic Centre, 5a, Lenina, Ploschad, Voronezh, 394108, Russian Federation. | Ministry of Healthcare and Social Development of the Russian Federation. Ethics Council, 3, Rahmanovskij pereulok, Moscow, 127994, Russian Federation.  Chairperson: Baybarina, Elena  Local Ethics Committee of State Healthcare Institution “Voronezh Regional Clinical Consultative Diagnostic Centre" 5a, Lenina Ploschad, 394018, Russian Federation.  Chairperson: Giorgadze, Marina. MD, PhD |
| City Clinical Hospital #4, 81, Ostrovskogo street, Chelyabinsk, 454106, Russian Federation. | Ministry of Healthcare and Social Development of the Russian Federation. Ethics Council, 3, Rahmanovskij pereulok, Moscow, 127994, Russian Federation.  Chairperson: Baybarina, Elena  Local Independent Ethic Committee attached to City Clinical Hospital #4,  81, Ostrovskogo street, Chelyabinsk, 454106, Russia.  Chairperson: Shiklanova, Elena |
| City Hospital #2, Pulmonolgy department, Admiralskogo str., 6, Pyatigorsk, 357538. Russian Federation. | Ministry of Healthcare and Social Development of the Russian Federation. Ethics Council, 3, Rahmanovskij pereulok, Moscow, 127994, Russian Federation.  Chairperson: Baybarina, Elena  Local Independent Ethics Committee attached to City Hospital #2, 6, Admiralskogo str., Pyatigorsk, 357538, Russian Federation.  Chairperson: Ayvazov , Valentin N |
| Novosibirsk State Regional Clinical Hospital, 130, Nemirovicha-Danchenko str, Novosibirsk, 630087, Russia. | Ministry of Healthcare and Social Development of the Russian Federation. Ethics Council, 3, Rahmanovskij pereulok, Moscow, 127994, Russia.  Chairperson: Baybarina, Elena  Local Ethics Committee attached to State Budget Healthcare Institution of Novosibirsk region, Novosibirsk regional Clinical Hospital, 130, Nemirovicha- Danchenko str. Novosibirsk, 630087, Russia.  Chairperson: Oleg, Levin |
| Municipal Hospital 5, 75, Zmeinogorskiy Higway, Barnaul, 656045, Russia. | Ministry of Healthcare and Social Development of the Russian Federation. Ethics Council, 3, Rahmanovskij pereulok, Moscow, 127994, Russia.  Chairperson: Baybarina, Elena  Local Ethics Committee attached to Municipal Healthcare Institution "City Hospital# 5, 75, Zmeinogorskiy Higway, Barnaul, 656045, Russia.  Chairperson: Vladimirovna, Kovchina S |
| Penza City Clinical Hospital #4, 1, Svetlaya str. 440067, Penza, Russian Federation. | Ministry of Healthcare and Social Development of the Russian Federation,Ethics Council, 127994, Moscow, Rahmanovskij pereulok, 3.  Russia.  Chairperson: Baybarina, Elena  Ethics committee under Penza State Institution of Advanced Medical Studies 8A Stasova ul. Penza 440060, Russian Federation.  Chairperson: Iskanderov, Bakhram G |
| Regional children consultative polyclinic, 44 K. Marksa str., Tomsk, 634001, Russia. | Ministry of Healthcare and Social Development of the Russian Federation. Ethics Council, 127994, Moscow, Rahmanovskij pereulok, 3. Russia.  Chairperson: Baybarina, Elena  Ethics committee of State educational institution of higher professional education “Siberian State medical university at Federal agency for healthcare and social development (Roszdrav)" 2, Moskovsky trakt, Tomsk.634050, Russia.  Chairperson: Bukreeva  ,Ekaterina |
| Far East Breath Pathology & Physiology Center, 22, Kalinina str., Blagoveshchensk, 675000, Russia. | Ministry of Healthcare and Social Development of the Russian Federation. Ethics Council, 127994, Moscow, Rahmanovskij pereulok, 3. Russia.  Chairperson: Baybarina, Elena  Biomedical Ethics committee of Far east Breath Pathology and Physiology Centre, 22, Kalinina str., Blagoveshchensk, 675000, Russia.  Chairperson: Nakhamchen, Dmitry |
| St. George the Martyr City Hospital 1, Sevemy pr., 194354,Saint-Petersburg  Russian Federation. | Ministry of Healthcare and Social Development of the Russian Federation. Ethics Council, 127994, Moscow, Rahmanovskij pereulok, 3. Russia.  Chairperson: Baybarina, Elena  Ethics Committee under St. George the Martyr City Hospital  1, Sevemy pr., 194354, Saint-Petersburg, Russian Federation.  Chairperson: Ivanovsky, Eduard |
| Municipal Healthcare institution “City Clinical Hospital #2 named after V.I Razumovsky”, 141, Chernyshevskogo str, Saratov, 410028, Russia. | Ministry of Healthcare and Social Development of the Russian Federation. Ethics Council, 127994, Moscow, Rahmanovskij pereulok, 3. Russia.  Chairperson: Baybarina, Elena |
| City Clinical Hospital #2, 6, korp.2, Krasnyh Partizan, Ul. Krasnodar, 350012 Russian Federation. | Ministry of Healthcare and Social Development of the Russian Federation. Ethics Council, 127994, Moscow, Rahmanovskij pereulok, 3. Russia.  Chairperson: Baybarina, Elena  Local Independent Ethics Committee attached to the Clinical Hospital #2, 6, korp.2, Krasnyh Partizan, Ul. Krasnodar, 350012, Russia.  Chairperson: Kovalevskaya ,Olga |
| City Consultative Diagnostic Center #1, Sikeyrosa street, 10, Saint-Petersburg, 194354, Russian Federation. | Ministry of Healthcare and Social Development of the Russian Federation. Ethics Council, 127994, Moscow, Rahmanovskij pereulok, 3. Russia.  Chairperson: Baybarina, Elena  Ethics Committee under St-Petersburg State Medical University, 10 Rentgena ul, 197101,St. Petersburg, Russian Federation.  Chairperson: Neznanov, Nikolay |
| Clinical Medical sanitary Unit #1, Therapy Dept.68, Gagarina boulevard, Perm, 614077, Russian Federation. | Ministry of Healthcare and Social Development of the Russian Federation. Ethics Council, 127994, Moscow, Rahmanovskij pereulok, 3. Russia.  Chairperson: Baybarina, Elena  Local Ethics Committee attached to Municipal Healthcare Institution Clinical Medical sanitary Unit #1, 68, Gagrina boulevard, Perm, 614010, Russian Federation.  Chairperson: Maslov, Yuriy |
| Research Institute of Clinical Immunology, 6, Zalesskogo street, Novosibirsk, 630047, Russia. | Ministry of Healthcare and Social Development of the Russian Federation. Ethics Council, 127994, Moscow, Rahmanovskij pereulok, 3. Russia.  Chairperson: Baybarina, Elena  Local Ethics Committee of Research Institute of Clinical Immunology, 6, Zalesskogo street, Novosibirsk, 630047, Russia.  Chairperson: Kozhevnikov , Vladimir |
| St. Petersburg State Medical University, Saint. Petersburg, Russian Federation, L. Tolstoy Street. 6/8, 197022, St. Petersburg, Russian Federation. | Ministry of Healthcare and Social Development of the Russian Federation. Ethics Council, 127994, Moscow, Rahmanovskij pereulok, 3. Russia.  Chairperson: Baybarina, Elena  Ethics Committee under St-Petersburg State Medical University, 10 Rentgena ul, 197101,St. Petersburg, Russian Federation.  Chairperson: Neznanov, Nikolay |
| City Outpatient Clinic #3  12 Enthuziastov ul, 347381, Volgodonsk, Russian Federation. | Ministry of Healthcare and Social Development of the Russian Federation. Ethics Council, 127994, Moscow, Rahmanovskij pereulok, 3. Russia.  Chairperson: Baybarina, Elena |
| **Ukraine** |  |
| Central City Clinical Hospital # 3, pediatric pulmonological department, 16 Ovnatanyana str., 83017 Donetsk, Ukraine. | Central Ethics Committee, Ministry of Health of Ukraine  5, Narodnogo Opolchennya street 03151, Kyiv, Ukraine.  Chairperson: Kornatskyi, Vasyl  Local Ethics committee, Central City Clinical Hospital # 3, 16 Ovnatanyana str., 83017 Donetsk, Ukraine.  Chairperson: Oriekhova, Valentina |
| Institute of Phthisiatry and Pulmonology, Dep. of technologies of treatment of NSPD, 10, Amosova str., 03680 Kyiv, Ukraine. | Central Ethics Committee, Ministry of Health of Ukraine  5, Narodnogo Opolchennya street 03151, Kyiv, Ukraine  Chairperson: Kornatskyi, Vasyl  Local Ethics committee, Institute of Phthisiatry and Pulmonology, 10, Amosova Street, 03680, Kyiv, Ukraine.  Chairperson: Melnyk, Vasyl |
| Institute of Phthisiatry and Pulmonology, Department of Pulmonology, 10, Amosova str., 03680 Kyiv, Ukraine. | Central Ethics Committee, Ministry of Health of Ukraine  5, Narodnogo Opolchennya street 03151, Kyiv, Ukraine  Chairperson: Vasyl, Kornatskyi  Local Ethics committee, Institute of Phthisiatry and Pulmonology, 10, Amosova Street, 03680, Kyiv, Ukraine.  Chairperson: Melnyk, Vasyl |
| Institute of Phthisiatry and Pulmonology, Department of Pediatric Pulmonology and Allergology,10, Amosova street, 03680, Kyiv, Ukraine | Central Ethics Committee, Ministry of Health of Ukraine  5, Narodnogo Opolchennya street 03151, Kyiv, Ukraine.  Chairperson: Vasyl , Kornatskyi  Local Ethics committee, Institute of Phthisiatry and Pulmonology, 10, Amosova Street, 03680, Kyiv, Ukraine.  Chairperson: Melnyk, Vasyl |
| Institute of Pediaitrics, Obstetrics and Gynecology, Department of child allergy problems and immunorehabilitation, 8 Manuilskogo street, 04050 Kyiv, Ukraine. | Central Ethics Committee, Ministry of Health of Ukraine  5, Narodnogo Opolchennya street 03151, Kyiv, Ukraine  Chairperson: Vasyl , Kornatskyi  Local Ethics Committee, Institute of Pediatrics, Obstetrics and Gynecology, 8 Manuilskogo street, 04050, Kyiv, Ukraine.  Chairperson: Tatarchuk, Tetiana |
| Kyiv city tuberculosis hospital #1, Dep. of diff. diagnostics of resp. organs diseases, Kharkivske shosse, 121/3, 02091 Kyiv, Ukraine. | Central Ethics Committee, Ministry of Health of Ukraine  5, Narodnogo Opolchennya street 03151, Kyiv, Ukraine  Chairperson: Vasyl , Kornatskyi  Local Ethics Committee, Kyiv city tuberculosis hospital #1, Kharkivske shosse, 121/3, 02091 Kyiv, Ukraine.  Chairperson: Panasiuk, Oleksii |
| Vinnytsia City Clinical Hospital #1, Pulmonology, 96 Khmelnytske Shosse, 21029 Vinnytsia, Ukraine. | Central Ethics Committee, Ministry of Health of Ukraine  5, Narodnogo Opolchennya street 03151, Kyiv, Ukraine  Chairperson: Vasyl , Kornatskyi  Local Ethics Committee, Vinnytsia City Clinical Hospital #1, 96 Khmelnytske Shosse, 21029 Vinnytsia, Ukraine.  Chairperson: Turskyi , Oleksandr |
| Zaporizhia City multi specialty children's hospital # 5, Department of allergology, 28-A, Novgorodskaya Street, 69076 Zaporizhia, Ukraine. | Central Ethics Committee, Ministry of Health of Ukraine  5, Narodnogo Opolchennya street 03151, Kyiv, Ukraine  Chairperson: Vasyl , Kornatskyi  Local Ethics Committee, Zaporizhia City multi specialty children's hospital # 5, 28-A, Novgorodskaya Street, 69076 Zaporizhia, Ukraine.  Chairperson: Bessikalo, Tetiana |
| Zaporizhia regional clinical children's hospital, Pulmonological department, Lenina avenue, 70, 69063. Zaporizhia, Ukraine. | Central Ethics Committee. Ministry of Health of Ukraine, 5, Narodnogo Opolchennya str., 03151, Kyiv, Ukraine  Chairperson: Vasyl , Kornatskyi  Local Ethics Committee, Zaporizhia regional clinical children’s hospital, Lenina avenue, 70, 69063, Zaporizhia, Ukraine.  Chairperson: Chychkanova, Galyna |
| City Clinical Hospital # 6, Department of therapy with pulmonological wards, 13 Batumska str., 49051 Dnipropetrovsk, Ukraine. | Central Ethics Committee. Ministry of Health of Ukraine, 5, Narodnogo Opolchennya str., 03151, Kyiv, Ukraine.  Chairperson: Kornatskyi, Vasyl  Local Ethics Committee, City Clinical Hospital # 6, Department of therapy with pulmonological wards, 13 Batumska str., 49051 Dnipropetrovsk, Ukraine.  Chairperson: Morenets Mykola |
| Kyiv City Clinical Hospital # 8, Allergological department # 1,Kondratyuka Str. 8, 04201, Kyiv, Ukraine. | Central Ethics Committee. Ministry of Health of Ukraine, 5, Narodnogo Opolchennya str., 03151, Kyiv, Ukraine.  Chairperson: Vasyl , Kornatskyi  Local Ethics Committee, Kyiv City Clinical Hospital # 8, Allergological department # 1,Kondratyuka Str. 8, 04201 Kyiv, Ukraine.  Chairperson: Chervak, Igor |
| Research Institute of Physical Methods of Treatment and Medical Climatology, Department of Pulmonology, 8, Mukhina Str., 98603,Yalta, Ukraine. | Central Ethics Committee. Ministry of Health of Ukraine, 5, Narodnogo Opolchennya str., 03151, Kyiv, Ukraine.  Chairperson: Vasyl, Kornatskyi  Local Ethics Committee, Research Institute of Physical Methods of Treatment and Medical Climatology, 8, Mukhina Str., 98603 Yalta, Ukraine.  Chairperson: Kovganko, Oleksii |
| State Institution «Research Centre for Radiation Medicine», AMS of Ukraine therapy of radiation consequences unit pulmonology department,119, 121, Peremogy avenue, Kyiv, 03115, Ukraine. | Central Ethics Committee. Ministry of Health of Ukraine, 5, Narodnogo Opolchennya str., 03151, Kyiv, Ukraine.  Chairperson: Vasyl , Kornatskyi  Local Ethics Committee, State Institution «Research Centre for Radiation Medicine», AMS of Ukraine 119, 121, Peremogy avenue, 03115, Kyiv, Ukraine.  Chairperson: Omelianets, Mykola |
| Crimean Republican Institution “Children`s Clinical Hospital”, Pulmonology department, 77, Titova str., 95034 Simferopol, Ukraine. | Central Ethics Committee. Ministry of Health of Ukraine, 5, Narodnogo Opolchennya str., 03151, Kyiv, Ukraine.  Chairperson: Vasyl , Kornatskyi  Local Ethics Committee, Crimean Republican Institution “Children`s Clinical Hospital”, Pulmonology department, 77, Titova str., 95034 Simferopol, Ukraine.  Chairperson: Usova, Nataliia |
| Kyiv City Children’s Hospital #2, Department of allergology, 3 Alishera Navoi str., 02660 Kyiv, Ukraine. | Central Ethics Committee. Ministry of Health of Ukraine, 5, Narodnogo Opolchennya str., 03151, Kyiv, Ukraine.  Chairperson: Vasyl, Kornatskyi  Local Ethics Committee, Kyiv City Children’s Hospital #2, Department of allergology, 3 Alishera Navoi str., 02660 Kyiv, Ukraine.  Chairperson: Raiosh, Valentina |
| **United States** |  |
| Allergy Asthma Research Institute, 333 Londonderry Drive, Suite No. 110, Waco, TX 76712. | Quorum Research Review, 1601 Fifth Avenue, Suite 1000, Seattle, WA 98101.  Chairperson: Kelley, David |
| Bensch Research Associates, 4632 Georgetown Place, Suite C, Stockton, CA 95207. | Quorum Research Review, 1601 Fifth Avenue, Suite 1000, Seattle, WA 98101.  Chairperson: Kelley, David |
| Southern California Research, 27800 Medical Center Road, Suite 240, Mission Viejo, CA 92691. | Quorum Research Review, 1601 Fifth Avenue, Suite 1000, Seattle, WA 98101.  Chairperson: Kelley David |
| Bernstein Clincal Research Center, LLC, 8444 Winton Road, Cincinnati, OH 45231. | Quorum Research Review, 1601 Fifth Avenue, Suite 1000, Seattle, WA 98101.  Chairperson: Kelley, David |
| CU Pharmaceutical Research, 1005 Thompson Boulevard Union, SC 29379. | Quorum Research Review, 1601 Fifth Avenue, Suite 1000, Seattle, WA 98101.  Chairperson: Kelley, David |
| Georgia Pollens Clinical Research Centers, Inc., 105 Spanish Court, Albany, GA 31707. | Quorum Research Review, 1601 Fifth Avenue, Suite 1000, Seattle, WA 98101.  Chairperson: Kelley, David |
| Brookstone Clinical Research Center, 1220 Brookstone Centre Parkway, Columbus, GA 31904. | Quorum Research Review, 1601 Fifth Avenue, Suite 1000, Seattle, WA 98101.  Chairperson: Kelley, David |
| Allergy & Asthma Specialists, PSC, 3604 Wathens Crossing, Owensboro, KY 42301. | Quorum Research Review, 1601 Fifth Avenue, Suite 1000, Seattle, WA 98101.  Chairperson: Kelley, David |
| Asthma Allergy & Pulmonary Associates, 1015 Chestnut Street Suite 1300, Philadelphia, PA 19107. | Quorum Research Review, 1601 Fifth Avenue, Suite 1000, Seattle, WA 98101.  Chairperson: Kelley, David |
| IPS Research Company, 1111 North Lee, Suite 400, Oklahoma City, OK 73103. | Quorum Research Review, 1601 Fifth Avenue, Suite 1000, Seattle, WA 98101.  Chairperson: Kelley, David |
| Henry D Covelli, MD, 700 W. Ironwood Drive, Suite 336, Coeur d'Alene, ID 83814. | Quorum Research Review, 1601 Fifth Avenue, Suite 1000, Seattle, WA 98101.  Chairperson: Kelley, David |
| Clinical Research Specialists, 3939 Houma Blvd, Bldg 6, Suite 20, Metairie, LA, 70006. | Quorum Research Review, 1601 Fifth Avenue, Suite 1000, Seattle, WA 98101.  Chairperson: Kelley, David |
| Allergy and Asthma Research Center, PA,  Suite 109, 2414 Babcock Road, San Antonio, TX, 78229. | Quorum Research Review, 1601 Fifth Avenue, Suite 1000, Seattle, WA 98101.  Chairperson: Kelley, David |
| Gaffney Pharmaceutical Research, 1529 N. Limestone St., Gaffney, SC 29340. | Quorum Research Review, 1601 Fifth Avenue, Suite 1000, Seattle, WA 98101.  Chairperson: Kelley, David |
| Florida Pulmonary Research Center, LLC, 1788 West Fairbanks Avenue, Suite B, Winter Park, FL 32789. | Quorum Research Review, 1601 Fifth Avenue, Suite 1000, Seattle, WA 98101.  Chairperson: Kelley, David |
| S. Carolina Pharmaceutical Research, 1330 Boiling Springs Rd, Suite 2100, Spartanburg, SC 29303. | Quorum Research Review, 1601 Fifth Avenue, Suite 1000, Seattle, WA 98101.  Chairperson: Kelley, David |
| Dawes Fretzin Clinical Research Group, LLC, 8103 Clearvista Parkway, Suites 220 and 260, Indianapolis, IN 46256. | Quorum Research Review, 1601 Fifth Avenue, Suite 1000, Seattle, WA 98101.  Chairperson: Kelley, David |
| National Allergy, Asthma & Urticaria Centers of Charleston, P.A., 1879 Savage Road, Charleston, SC 29407. | Quorum Research Review, 1601 Fifth Avenue, Suite 1000, Seattle, WA 98101.  Chairperson: Kelley, David |
| Allergy and Respiratory Center, 4048 Dressler Road NW, Canton, OH, 44718. | Quorum Research Review, 1601 Fifth Avenue, Suite 1000, Seattle, WA 98101.  Chairperson: Kelley, David |
| Allergy and Asthma research of NJ, Inc., 2137 Welsh Road, Suite 2B, Philadelphia, PA 19115. | Quorum Research Review, 1601 Fifth Avenue, Suite 1000, Seattle, WA 98101.  Chairperson: Kelley, David |
| Island Medical Research, Suite 401, 242 Merrick Road, Rockville Centre, NY 11570. | Quorum Research Review, 1601 Fifth Avenue, Suite 1000, Seattle, WA 98101.  Chairperson: Kelley, David |
| Advances in Medicine, 42362 Bob Hope Drive, Rancho Mirage, CA 92270. | Western Institutional Review Board, 3535 7th Avenue SW, Olympia, WA 98502.  Chairperson: Schultz, Theodore |
| Colorado Allergy and Asthma Center, PC, 14000 East Arapahoe Road, Suite 260, Centennial, CO 80112. | Quorum Research Review, 1601 Fifth Avenue, Suite 1000, Seattle, WA 98101.  Chairperson: Kelley, David |
| Brandon-Valrico Center for Allergy and Asthma Research, LLC, 3658 Lithia Pinecrest Road, Valrico, FL 33596. | Quorum Research Review, 1601 Fifth Avenue, Suite 1000, Seattle, WA 98101.  Chairperson: Kelley, David |
| The Allergy and Asthma Clinic of Central Texas, 4204 Stan Schlueter Loop, Killeen, TX 76542. | Quorum Research Review, 1601 Fifth Avenue, Suite 1000, Seattle, WA 98101.  Chairperson: Kelley, David |
| Montefiore Medical Center, 1515 Blondell Avenue, Suite 220, Bronx, NY 10461. | Biomedical Research Alliance of New York, 1981 Marcus Avenue, Suite 210, Lake Success, NY 11042  Chairperson: Keith Krasinski |
| NextCare Institute for Clinical Research, 1701 E. Thomas Road, Suite A104, Phoenix, AZ 85016. | Quorum Research Review, 1601 Fifth Avenue, Suite 1000, Seattle, WA 98101.  Chairperson: Kelley, David |
| University Central Medical Specialty Clinic, 2828 Fresno Street, Fresno CA 93721. | Community Medical Centers Institutional Review Board,  155 North Fresno Street, Suite 290, Fresno CA 93701.  Chairperson: Libke, Robert |
| Austin Clinical Research, Inc., 12416 Hymeadow Drive, Suite 101, Austin, TX 78750. | Quorum Research Review, 1601 Fifth Avenue, Suite 1000, Seattle, WA 98101.  Chairperson: Kelley, David |
| Oak Street Medical, P.C, Allergy & Asthma Research Group, 1488 Oak Street, Eugene, OR 97401. | Quorum Research Review, 1601 Fifth Avenue, Suite 1000, Seattle, WA 98101.  Chairperson: Kelley, David |
| Timber Lane Allergy & Asthma Research, LLC  54 Timber Lane,  South Burlington, VT 05403. | Quorum Research Review, 1601 Fifth Avenue, Suite 1000, Seattle, WA 98101.  Chairperson: Kelley, David |
| Clinical Research Institute of Southern Oregon, PC, 3860 Crater Lake Avenue, Suite B, Medford, OR 97504. | Quorum Research Review, 1601 Fifth Avenue, Suite 1000, Seattle, WA 98101.  Chairperson: Kelley, David |
| Trinity Medical Research, Inc, 576 N. Sunrise Avenue, Suite 220, Roseville, CA 95661. | Quorum Research Review, 1601 Fifth Avenue, Suite 1000, Seattle, WA 98101.  Chairperson: Kelley, David |
| Asthma and Allergy Center of Chicago, SC, 7420 Central , Suite 2020, River Forest, IL 60305. | Quorum Research Review, 1601 Fifth Avenue, Suite 1000, Seattle, WA 98101.  Chairperson: Kelley, David |
| San Diego Sports Medicine and Family Health Center, 6699 Alvarado Rd, Suite 2100, San Diego, CA 92120. | Quorum Research Review, 1601 Fifth Avenue, Suite 1000, Seattle, WA 98101.  Chairperson: Kelley, David |
| Medical Research of Arizona, A Division of Alergy, Asthma & Immunology Associates Ltd., 7514 East Monterey Way Suite 1A, Scottsdale, AZ 85251. | Quorum Research Review, 1601 Fifth Avenue, Suite 1000, Seattle, WA 98101.  Chairperson: Kelley, David |
| Rameshwar N Mathur, MD, PA, 6250 North US 1, Cocoa, FL, 32927. | Quorum Research Review, 1601 Fifth Avenue, Suite 1000, Seattle, WA 98101.  Chairperson: Kelley, David |
| Chesapeake Clinical Research, Inc., 7939 Honeygo Boulevard, Suite 219, Baltimore MD 21236. | Quorum Research Review, 1601 Fifth Avenue, Suite 1000, Seattle, WA 98101.  Chairperson: Kelley, David |
| Allergy and Asthma Care Center of So. Calif, 3816 Woodruff Avenue # 209, Long Beach, CA 90808. | Quorum Research Review, 1601 Fifth Avenue, Suite 1000, Seattle, WA 98101.  Chairperson: Kelley, David |
| Allergy & Clinical Immunology Associates, 180 Fort Couch Road, Suite 375, Pittsburgh, PA 15241. | Quorum Research Review, 1601 Fifth Avenue, Suite 1000, Seattle, WA 98101.  Chairperson: Kelley, David |
| Florida Center for Allergy & Asthma Research, 9035 Sunset Drive, Suite 202, Miami, FL 33173. | Quorum Research Review, 1601 Fifth Avenue, Suite 1000, Seattle, WA 98101.  Chairperson: Kelley, David |
| Integrated Research Group. Inc., 4646 Brockton Avenue,  Suites 101/202/203/301,  Riverside, CA 92506. | Quorum Research Review, 1601 Fifth Avenue, Suite 1000, Seattle, WA 98101.  Chairperson: Kelley, David |
| Colorado Allergy & Asthma Centers, PC 125 Rampart Way, Suite 150, Denver, CO, 80230. | Quorum Research Review, 1601 Fifth Avenue, Suite 1000, Seattle, WA 98101.  Chairperson: Kelley, David |
| North Bay Clinical Trials, 1100 Pear Tree Lane, Napa, CA 94558. | Quorum Research Review, 1601 Fifth Avenue, Suite 1000, Seattle, WA 98101.  Chairperson: Kelley, David |
| Pediatric Care Medical Group, Inc, 17822 Beach Boulevard, Suite 278, Huntington Beach, CA 92647. | Quorum Research Review, 1601 Fifth Avenue, Suite 1000, Seattle, WA 98101.  Chairperson: Kelley, David |
| Sunrise Clinical Research  5985 Florence Ave # N  Bell Gardens, CA, 90201. | Quorum Research Review, 1601 Fifth Avenue, Suite 1000, Seattle, WA 98101.  Chairperson: Kelley, David |
| Pulmonary Associates of Mobile PC, 6701 Airport Blvd, Suite B131, Mobile, AL 36608. | Quorum Research Review, 1601 Fifth Avenue, Suite 1000, Seattle, WA 98101.  Chairperson: Kelley, David |
| PA Allergy and Asthma Consultants, P.C, 555 Second Ave, Suite C-750, Collegeville, PA 19425. | Quorum Research Review, 1601 Fifth Avenue, Suite 1000, Seattle, WA 98101.  Chairperson: Kelley, David |
| Peninsula Research Associates, Inc., 550 Deep Valley Drive, Ste 317, Rolling Hills Est, CA 90274. | Quorum Research Review, 1601 Fifth Avenue, Suite 1000, Seattle, WA 98101.  Chairperson: Kelley, David |
| Sunset Medical Research, 990 Napoloean Avenue Sunset, LA 70584. | Quorum Research Review, 1601 Fifth Avenue, Suite 1000, Seattle, WA 98101.  Chairperson: Kelley, David |
| Peninsula Pulmonary Medical Associates, 23550 Hawthorne Blvd, Suite 180, Torrance, CA 90505. | Quorum Research Review, 1601 Fifth Avenue, Suite 1000, Seattle, WA 98101.  Chairperson: Kelley, David |
| Allergy & Asthma Associates, 7707 Fannin, Suite 200, Houston, TX 77054. | Quorum Research Review, 1601 Fifth Avenue, Suite 1000, Seattle, WA 98101.  Chairperson: Kelley, David |
| Alabama Allergy and Asthma Center, LLC, 10 Old Montgomery Highway, Suite 100, Birmingham, AL 35209. | Quorum Research Review, 1601 Fifth Avenue, Suite 1000, Seattle, WA 98101.  Chairperson: Kelley, David |
| Allergy and Asthma Center, LLC, 565 Old Norcross Road, Suite 101, Lawrenceville, GA 30046. | Quorum Research Review, 1601 Fifth Avenue, Suite 1000, Seattle, WA 98101.  Chairperson: Kelley, David |
| Allergy Asthma & Clinical Research Center, 4200 W. Memorial Rd, Ste. 206, Oklahoma City, OK 73120. | Quorum Research Review, 1601 Fifth Avenue, Suite 1000, Seattle, WA 98101.  Chairperson: Kelley, David |
| Southern California Institute for Respiratory Disease, 8635 West 3rd Street, STE 965W Los Angeles, CA 90048. | Quorum Research Review, 1601 Fifth Avenue, Suite 1000, Seattle, WA 98101.  Chairperson: Kelley, David |
| Clinical Trial Network, 8303 SouthWest Freeway, Suite 150, Houston, TX 77074. | Quorum Research Review, 1601 Fifth Avenue, Suite 1000, Seattle, WA 98101.  Chairperson: Kelley, David |
| Toledo Center for Clinical Research, 5860 Alexis Road, Suite B, Sylvania, OH 43560. | Quorum Research Review, 1601 Fifth Avenue, Suite 1000, Seattle, WA 98101.  Chairperson: Kelley, David |

**Study FFA109685: 98 Centres**

| **FFA 109685**  **Hospital/ Institution and Address** | **FFA 109685**  **IEC/IRB Committee Chair and Name of Committee** |
| --- | --- |
| **CANADA** |  |
| Aggarwal and Associates Ltd., 40 Finchgate Blvd, Suite 314, Brampton ON L6T 3J1 Canada | Optimum Clinical Research Inc., 231 King Street East, Oshawa ON L1H 1C5 Canada  Chairperson- Bill Wilson |
| Centre de Recherche Appliquee en Allergie de Quebee, 2590 Boulevard Laurier, 2e etage, Bureau 225, Quebec QC G1V 4M6 Canada | Optimum Clinical Research Inc., 231 King Street East, Oshawa ON L1H 1C5 Canada  Chairperson- Bill Wilson |
| Centre de Pneumologie, Pavillon U ler etage local 1753, 2725 Ch Ste-Foy, Quebec QC G1V 4G5 Canada | Comite d ethique de la recherché de l’hopital Laval 2725 Ch Ste-Foy, Quebec QC G1V 4G5 Canada  Chairperson- Franck Molin |
| Dr. Calvin Powell Professional Medical Corporation, 286 Conception Bay Hwy, P.O. Box 1017, Bay Roberts NL A0A 1G0 Canada | Optimum Clinical Research Inc, 604 Taunton Road West, Oshawa ON L1H 7K4 Canada  Chairperson- Bill Wilson |
| Allergy & Asthma Research Centre, 707-1081 Carling Ave, Ottawa ON K1Y 4G2 Canada | Optimum Clinical Research Inc., 231 King Street East, Oshawa ON L1H 1C5 Canada  Chairperson- Bill Wilson |
| **ESTONIA** |  |
| Tartu University Clinics, Lung Clinic, 167 Riia Str, Tartu 51014, Estonia | Ethics Review Committee (Erc) On Human Research Of The University Of Tartu, Ulikooli Str 18, Tartu, 50090, Estonia  Chairperson- Andres Soosaar |
| Tallinn Children's Hospital, Tervise 28, Tallinn 13419, Estonia | Ethics Review Committee (ERC) On Human Research Of The University If Tartu, Ulikooli str.18, Tartu, 50090, Estonia  Chairperson- Andres Soosaar |
| As Medicum, Punane 61, Tallinn 13619, Estonia | Ethics Review Committee (ERC) On Human Research Of The University If Tartu, Ulikooli str.18, Tartu, 50090, Estonia  Chairperson- Andres Soosaar |
| TARTU UNIVERSITY CLINICS, CHILDREN’S CLINIC, LUNINI 6, TARTU 51014, ESTONIA | Ethics Review Committee (Erc) On Human Research Of The University Of Tartu, Ulikooli Str 18, Tartu, 50090, Estonia  Chairperson- Andres Soosaar |
| **GERMANY** |  |
| Praxis Dr. med. Bamberg, Hebbelstr. 6, 14469 Potsdam, Germany | Ethik-Kommission der, Aerztekammer Schleswig-Holstein, Bismarckallee 8-12, 23795 Bad Segeberg, Germany  Chairperson- Thomas H. Huetteroth |
| Praxis Dr. med. Benedix, Schonstr. 5-7, 13086 Berlin, Germany | Ethik-Kommission der Aerztekammer Schleswig-Holstein, Bismarckallee 8-12, 23795 Bad Segeberg, Germany  Chairperson- Thomas H. Huetteroth |
| Praxis Dr. med. Forster, Mollendorfstr. 111, 10367 Berlin, Germany | Ethik-Kommission der, Aerztekammer Schleswig-Holstein, Bismarckallee 8-12, 23795 Bad Segeberg, Germany  Chairperson- Thomas H. Huetteroth |
| Praxis Dr. med. Gehling, Markgrafenstr. 2, 68723 Schwetzingen, Germany | Ethik-Kommission der, Landesaerztekammer Baden-Wuerttemberg, Jahnstrasse 38 A, 70597 Stuttgart  Chairperson- Georg Hook |
| Praxis Dr. med. Linnhoff, Hohenzollerndamm 2, 10717, Berlin, Germany | Ethik-Kommission der, Aerztekammer Schleswig-Holstein, Bismarckallee 8-12, 23795 Bad Segeberg, Germany  Chairperson- Thomas H. Huetteroth |
| Praxis Dr. med. von Versen, Yorckstr. 71, 10965 Berlin, Germany | Ethik-Kommission der, Aerztekammer Schleswig-Holstein, Bismarckallee 8-12, 23795 Bad Segeberg, Germany. Chairperson- Thomas H. Huetteroth |
| Praxis Dr. med. Zachgo, Norderstr. 12, 21502 Geesthacht, Germany | Ethik-Kommission der Aerztekammer Schleswig-Holstein, Bismarckallee 8-12, 23795 Bad Segeberg, Germany  Chairperson- Thomas H. Huetteroth |
| **GREECE** |  |
| B' Respiratory Clinic, General Hospital of Kavala, 113 Amerikanikou Erythrou Stavrou, 65403, Kavala, Greece | National Drug Organisation (EOF) and National Ethics Committee (EED), 284 Mesoghion Avenue, Cholargos, 15 562, Athens, Greece  Chairperson- Ioannis Papadimitriou |
| 7th Pulmonary Clinic, Chest Hospital of Athens "Sotiria", 152 Mesoghion Avenue, 115 27, Papagos, Athens, Greece | National Drug Organisation (EOF) and National Ethics Committee (EED), 284 Mesoghion Avenue, Cholargos, 15 562, Athens, Greece  Chairperson- Ioannis Papadimitriou |
| University Pulmonary Clinic, "G. Papanikolaou" General Hospital of Thessaloniki, Exohi, Asvestohoriou, 57010, Thessaloniki, Greece | National Drug Organisation (EOF) and National Ethics Committee (EED), 284 Mesoghion Avenue, Cholargos, 15 562, Athens, Greece  Chairperson- Ioannis Papadimitriou |
| University Pulmonary Clinic, University Hospital of Thessalia, Mezourlo, 41110, Larissa, Greece | National Drug Organisation (EOF) , 284 Mesoghion Avenue, Cholargos, 15 562, Athens, Greece  Chairperson- Ioannis Papadimitriou |
| Papageorgiou General Peripheral Hospital, Respiratory Dept, Peripheral Road of Thessaloniki, N. Efkarpia, 564 29, Thessaloniki, Greece | National Drug Organisation (EOF) and National Ethics Committee (EED), 284 Mesoghion Avenue, Cholargos, 15 562, Athens, Greece  Chairperson- Ioannis Papadimitriou |
| General Hospital of Rethymnon, Respiratory Clinic, Trandalidou 17, Rethymnon, 74100, Crete, Greece | National Drug Organisation (EOF) and National Ethics Committee (EED), 284 Mesoghion Avenue, Cholargos, 15 562, Athens, Greece  Chairperson- Ioannis Papadimitriou |
| **KOREA** |  |
| Seoul National University Hospital 28 Yongon-Dong, Chongno-Gu, Seoul 110-744, Korea | Seoul National University Hospital Institutional Review Board, 28 Yongon-Dong, Chongno-gu, Seoul 110-744, Korea  Chairperson- Sang-Goo Shin |
| Chonnam National University Hospital, 8 Hakdong, Dongku Gwangju 501-757 Republic of Korea | Chonnam National University Hospital Institutional Review Board, 8 Hakdong, Dongku Gwangju 501-757 Republic of Korea  Chairperson- Young-Ho Won |
| Chungbuk National University Hopsital, 410 Gaesin-dong, Heungdeok-gu, Cheongju, Chungcheongbuk-do(361-711), Korea | Chungbuk National University Hopsital Institutional Review Board, 410 Gaesin-dong, Heungdeok-gu, Cheongju, Chungcheongbuk-do(361-711), Korea  Chairperson- See Ok Shin |
| Division of Allergy and Pulmonology, Soon Chun Hyang University Bu Cheon Hospital, 1174 Jung-Dong, Wonmi-Gu, Bucheon-Si, Gyeonggi-Do, 420-021, Korea | Institutional Review Board (IRB), Soon Chun Hyang University Bu Cheon Hospital, 1174 Jung-Dong, Wonmi-Gu, Bucheon-Si, Gyeonggi-Do, 420-021, Korea  Chairperson- Hwang , Seung Duk |
| Ajou University Hospital, San 5, Wonchon-dong, Yeongtong-gu Suwon 443-721 Korea | Ajou University Hospital Regional Clinical Trial Center, San 5, Wonchon-dong, Yeongtong-gu Suwon 443-721 Korea  Chairperson- Chun, Mi Son |
| Yonsel University, College of Medicine, Severance Hospital, 134 Shinchon-dong, Seodaemun-gu, Seoul, 120-752 Korea | Institutional Review Board , Yonsel University, College of Medicine, Severance Hospital, 134 Shinchon-dong, Seodaemun-gu, Seoul, 120-752 Korea  Chairperson- Jong Tae Lee |
| **MEXICO** |  |
| Torre ltalis, Avenida Copemico 3817 consultorio 14 esquina Sagitario, Colonia Arboledas, Zapopan, Jalisco, C P. 45040, Mexico | Comité de Etica del Hospital Real: San José Av. Lázaro Cárdenas 4149 Colonia Jardines de San Ignacio Zapopan, Jalisco C.P. 45040 México  Chairperson- Joaquín Jiménez Flores |
| Instituto Jalisciense de Investigacion Clinica, S.A. de C.V., Penitenciaria 20 Colonia Centro, Guadalajara, Jalisco, C.P. 44100, Mexico | Comite de Etica del Instituto Jalisciense de Investigacion Clinica, S.A. de C.V., Penitenciaria 20 Colonia Centro, Guadalajara, Jatisco, C.P. 44100, Mexico  Chairperson- Joaquín Jiménez Flores |
| **PHILIPPINES** |  |
| Veterans Memorial Medical Centre, Department of Pulmonary Diseases, North Avenue, Dillman, Quezon City, Philippines 1101 | Veterans Memorial Medical Centre Research and Ethics Committee, Veterans Memorial Medical Centre, Department of Pulmonary Diseases, North Avenue, Dillman, Quezon City, Philippines 1101  Chairperson- Emerita A. Barrenechea |
| Lung Centre of The Philippines, Department of Pulmonary Medicine, Quezon Avenue, Quezon City, Philippines 1104 | Lung of the Philippines Ethics Review Committee, Lung Center of the Philippines, Department of Pulmonary Medicine, Quezon Avenue, Quezon City, Philippines 1104  Chairperson- Primitivo S. Guinit |
| Philippine Heart Center, Pulmonary and Critical Care Medicine, East Avenue, Quezon City, Philippines 1100 | Philippine Heart Center Institutional Review Board, Philippine Heart Center, Pulmonary and Critical Care Medicine, East Avenue, Quezon City, Philippines 1100  Chairperson- Marcelito L. Durante |
| Quirino Memorial Medical Centre, Katipunan Road Corner P. Tuazon Avenue, Quezon City, Philippines 1109 | Quirino Memorial Medical Centre Research and Ethics Committee, Quirino Memorial Medical Centre, Katipunan Road Corner P. Tuazon Avenue, Quezon City, Philippines 1109  Chairperson- Evelyn Reside |
| **POLAND** |  |
| Klinika Alergologii, Akademickie Centrum Kliniczne - Szpital AMG ul. Debinki 7, 80-952 Gdansk, Poland | Komisja Bioetyczna, Dolnoslaska lzba Lekarska ul. Matejki 6, 50-333 Wroclaw, Poland  Chairperson- Wlodzimierz Bednorz |
| NZOZ ALL-MED Specjalistyczna Opieka Medyczna ul. Pulaskiego 42G, 50-445 Wroclaw, Poland | Komisja Bioetyczna, Dolnoslaska lzba Lekarska ul. Matejki 6, 50-333 Wrociaw, Poland  Chairperson- Wlodzimierz Bednorz |
| **ROMANIA** |  |
| Cabinet Medical, Alergologie. Bd. Basarabiei 19, sector 2, cod postal 022102, Bucuresti, Romania | National Drug Agency, 48 Aviator Sanatescu Street. District 1. postal code 011478, Bucharest, Romania  Chairperson- Sava Dumitrescu  National Ethics Committee, 48 Aviator Sanatescu Street, District 1, postal code 011478, Bucharest, Romania  Chairperson- Sava Dumitrescu |
| SPITALUL JUDETEAN DEVA, STR 22 DECEMBRIE NR. 55, COD POSTAL 330084, DEVA, ROMANIA | National Drug Agency, 48 Aviator Sanatescu Street, District 1, postal code 011478, Bucharest, Romania  Chairperson- Sava Dumitrescu  National Ethics Committee, 48 Aviator Sanatescu Street. District 1. postal code 01 1478. Bucharest, Romania  Chairperson- Sava Dumitrescu |
| Institutul National de Medicina Aeronautica, Str. M. Vulcanescu nr. 88, cod postal 010825, Bucuresti, Romania | National Drug Agency, 48 Aviator Sanatescu Street, District 1, postal code 011478, Bucharest, Romania  Chairperson- Sava Dumitrescu  National Ethics Committee, 48 Aviator Sanatescu Street. District 1. postal code 01 1478. Bucharest, Romania  Chairperson- Sava Dumitrescu |
| Cabinet Medical,MEDRES, Sarguintei nr. 312, 540143, Targu Mures. Romania | National Drug Agency, 48 Aviator Sanatescu Street, District 1, postal code 011478, Bucharest, Romania  Chairperson- Sava Dumitrescu  National Ethics Committee, 48 Aviator Sanatescu Street. District 1. postal code 01 1478. Bucharest, Romania  Chairperson- Sava Dumitrescu |
| **RUSSIAN** **FEDERATION** |  |
| FGU Scientific Research Institute of Pulmonolgy, 11-th Parkovaya str., 32/61 Moscow, Russia, 105077 | Ethics Committee within Federal Service on Surveillance in Healthcare and Social Development of Russian Federation, Russia, 109074, Moscow, Slavyanskaya pl., 4, Bldn. 1  Ethics Committee attached to the FGU Scientific Research Institute of Pulmonolgy, 11-th Parkovaya str., 32/61 Moscow, Russia, 105077  Chairperson- Andrey L. Chernyaev |
| Federal State Institution of Presidential Properly Management Department of the Russian Federation, Out-Patient Clinic #3, 31, Grokholsky str., Moscow, 129090, Russia | Ethics Committee within Federal Service on Surveillance in Healthcare and Social Development of Russian Federation, Russia, 109074, Moscow, Slavyanskaya pl., 4, Bldn. 1  Ethics Committee attached to the Federal State Institution of Presidential Properly Management Department of the Russian Federation, Out-Patient Clinic #3, 31, Grokholsky str., Moscow, 129090, Russia  Chairperson- Elizaveta S. Begotskaya |
| Smolensk City Clinical Hopsital #1, Frunze street, 40, Smolensk, 214008, Russia | Ethics Committee within Federal Service on Surveillance in Healthcare and Social Development of Russian Federation, Russia, 109074, Moscow, Slavyanskaya pl., 4, Bldn. 1  Ethics Committee attached to the State Educational Institution of Higher Professional Education “Smolensk State Medical Academy of Federal Agency of Healthcare and Social Development”, ul. Krupskoy, 28, Smolensk, 214019, Russia  Chairperson- Alexander V. Litvinov |
| Regional Children Consultative Polyclinic, 44, Carl Marx street, Tomsk, 634034, Russia | Ethics Committee within Federal Service on Surveillance in Healthcare and Social Development of Russian Federation, Russia, 109074, Moscow, Slavyanskaya pl., 4, Bldn. 1  Chairperson: None at present  Ethics Committee attached to the State Educational Institution of Higher Professional Education “Siberian State Medical University of Federal Agency for Health and Social Development”, 2, Moskovsky trakt, Tomsk, 634050, Russia  Chairperson- Ekaterina B. Bukreeva |
| City Clinical Hospital #13, Velozavodskaya street, 1/1, Moscow, 115280, Russia | Ethics Committee within Federal Service on Surveillance in Healthcare and Social Development of Russian Federation, Russia, 109074, Moscow, Slavyanskaya pl., 4, Bldn. 1  Ethics Committee attached to the City Clinical Hospital #13, Velozavodskaya street, 1/1, Moscow, 115280, Russia  Chairperson- Olga B. Semeshkina |
| Moscow City Clinical Hopsital #23 n.a. Medsantrud, Pulmonolgy Department, Yauzskaya str., 11, bl. 4, Moscow, 109240, Russia | Ethics Committee within Federal Service on Surveillance in Healthcare and Social Development of Russian Federation, Russia, 109074, Moscow, Slavyanskaya pl., 4, Bldn. 1  Ethics Committee attached to the Moscow City Clinical Hopsital #23 n.a. Medsantrud, Pulmonolgy Department, Yauzskaya str., 11, bl. 4, Moscow, 109240, Russia  Chairperson- Tamara A. Chursina |
| **SLOVAKIA** |  |
| MUDr. Dagmar Klcova,, Klinicka imunologia a alergologia, Halalovka 61, 911 08 Trencin, Slovakia | Eticka komisia FNsP Bratislava, Pracovisko Ruzinov, Ruzinovska 6, 826 06 Bratislava, Slovakia  Chairperson- Anna Krechnakova |
| **SOUTH AFRICA** |  |
| University of Capetown Institute, George Street, Mowbray, 7700, Cape Town, South Africa | University of Cape Town Research Ethics Committee, Health sciences faculty, Room E53-24 Groote Schuur Hospital, Old Main Building, Observatory, 7925, Cape Town, South Africa.  Chairperson- M. Blockman |
| 20 Dorp Street, panorama, 7506, Cape Town, South Africa | Pharma- Ethics Independent Research Ethics Committee, 123 Amcor Road, Lyttelton Manor 0157, Pretoria South Africa  Chairperson- C.S.J. Duvenage |
| **UNITED** **STATES** |  |
| Alatae Medical, LLC, 390 Amwell Road, Building 5, Suite 501, Hillsborough, NJ 08844 | Copernicus Group IRB, One Triangle Drive, Suite 100, PO Box 110605, RTP, NC 27709  Chairperson- Glenn C. Veit |
| Allergy Asthma Research Institute, 333 Londonderry Drive, Suite 110, Waco, TX 76712 | Copernicus Group IRB, One Triangle Drive, Suite 100, PO Box 110605, RTP, NC 27709  Chairperson- Glenn C. Veit |
| Manassas Clinical Research Center, 9001 Digges Road, Suite 105, Manassas, VA 20110 | Copernicus Group IRB, One Triangle Drive, Suite 100, PO Box 110605, RTP, NC 27709  Chairperson- Glenn C. Veit |
| Bernatein Clinical Research Center 8444 Winton Road Cincinnati, Ohio 45231. | Copernicus Group IRB One Triangle Drive, Suite 100 P.O. Box 110605 RTP, NC 27709  Chairperson- Glenn C. Veit |
| Iowa Clinical Research Corporation 225 East Burlington Street Iowa City, IA 52240. | Copernicus Group IRB One Triangle Drive, Suite 100 P.O. Box 110605 RTP, NC 27709  Chairperson- Glenn C. Veit |
| Mayo Clinic 200 First Street SW Rochester, MN 55905 | Mayo Institutional Review Board Mayo Clinic 200 First Street SW Rochester, MN 55905  Chairperson- Bart L.Clark |
| Adviso Medical Research, L.L.C. 7906 S. Crandon Ave. Suite #4 Chicago, IL. 60617. | Copernicus Group IRB One Triangle Drive, Suite 100 P.O. Box 110605 RTP, NC 27709  Chairperson- Glenn C. Veit |
| Florida Pulmonary Research Center 1788 West Fairbanks Ave.. Suite B Winter Park, Florida 32789 | Copernicus Group IRB One Triangle Drive, Suite 100 P.O. Box 110605 RTP, NC 27709  Chairperson- Glenn C. Veit |
| College Park Family Care Center Multi-Speciality Clinical Research 11725 W. 112^th^ Street Overland Park, KS 66210. | Copernicus Group IRB One Triangle Drive, Suite 100 P.O. Box 110605 RTP, NC 27709  Chairperson- Glenn C. Veit |
| Allergy & Respiratory Center 4048 Dressler Road, NW Canton, Ohio 44718. | Copernicus Group IRB One Triangle Drive, Suite 100 P.O. Box 110605 RTP, NC 27709  Chairperson- Glenn C. Veit |
| Island Medical Research, P.C. 242 Merrick Road, Suite 401 Rockville Centre, NY11570 | Copernicus Group IRB One Triangle Drive, Suite 100 P.O. Box 110605 RTP, NC 27709  Chairperson- Glenn C. Veit |
| Northern Illinois Research Associates 625 E. Bethany Road Suite 3 DeKalb, IL 60115. | Copernicus Group IRB One Triangle Drive, Suite 100 P.O. Box 110605 RTP, NC 27709  Chairperson- Glenn C. Veit |
| Marycliff Allergy Specialists 823 W 7^th^ Ave Spokane, WA 99204. | Copernicus Group IRB One Triangle Drive, Suite 100 P.O. Box 110605 RTP, NC 27709  Chairperson- Glenn C. Veit |
| Mississippi Asthma & Allergy Clinic, P.A. 1600 North State Street Suites 101 & 201 Jackson, MS 39202. | Copernicus Group IRB One Triangle Drive, Suite 100 P.O. Box 110605 RTP, NC 27709  Chairperson- Glenn C. Veit |
| West Coast Clinical Trials Phase 2-4, LLC, 2600 Redondo Avnue, 4^th^ Floor, Suite #401, Long Beac, CA 90860 | Copernicus Group IRB One Triangle Drive, Suite 100 P.O. Box 110605 RTP, NC 27709  Chairperson- Glenn C. Veit |
| North Texas Family Medicine 4001 W. 15^th^ street, Suite 445 Plano, TX 75093 | Copernicus Group IRB One Triangle Drive, Suite 100 P.O. Box 110605 RTP, NC 27709  Chairperson- Glenn C. Veit |
| East Carolina University Physicians’ Moye Medical Center 521-A Moye Blvd. Greenville, NC 27834 | University and Medical Center Institutional Review Board, East Carolina University, Ed Warren Life Sciences Building, 600 Moye Boulevard, LSB 104, Greenville, NC 27834  Chairperson- L.Wiley Nifong |
| Allergy & Asthma Research Group 1488 Oak Street Eugene, OR 97401-4043 | Copernicus Group IRB One Triangle Drive, Suite 100 P.O. Box 110605 RTP, NC 27709  Chairperson- Glenn C. Veit |
| Timber Lane Allergy & Asthma Research, LLC 50 Timber Lane South Burlington, VT 05403 | Copernicus Group IRB One Triangle Drive, Suite 100 P.O. Box 110605 RTP, NC 27709  Chairperson- Glenn C. Veit |
| Clinical Research Institute of Southern Oregon, PC 3850 Crater Lake Avenue, Suite B, Medford, OR 97504 | Copernicus Group IRB One Triangle Drive, Suite 100 P.O. Box 110605 RTP, NC 27709  Chairperson- Glenn C. Veit |
| The Clinical Research Center, L.L.C. 1040 North Mason Road, Suite 112 St. Louis, Missouri 63141 | Copernicus Group IRB One Triangle Drive, Suite 100 P.O. Box 110605 RTP, NC 27709  Chairperson- Glenn C. Veit |
| North Carolina Clinical Research 4301 Lake Boone Trail, Suite 309-A Raleigh, NC 27607. | Copernicus Group IRB One Triangle Drive, Suite 100 P.O. Box 110605 RTP, NC 27709  Chairperson- Glenn C. Veit |
| Clinical Research of the Ozarks, Inc 407A East Russell Avenue, Suite 3 Warrensburg, MO 64093 | Copernicus Group IRB One Triangle Drive, Suite 100 P.O. Box 110605 RTP, NC 27709  Chairperson- Glenn C. Veit |
| Rocky Mt Center for Clinical Research 8550 West 38^th^ Ave.,# 202 Wheat Ridge, CO 80033 | Copernicus Group IRB One Triangle Drive, Suite 100 P.O. Box 110605 RTP, NC 27709  Chairperson- Glenn C. Veit |
| San Diego Sports Meducine and Family Health Center 6699 Alvarado Rd. Suite 2100 San Diego Ca 92120 | Copernicus Group IRB One Triangle Drive, Suite 100 P.O. Box 110605 RTP, NC 27709  Chairperson- Glenn C. Veit |
| AARA Research Center 9900 N. Central Expressway Suite 555 Dallas, Texas 75231 | Copernicus Group IRB One Triangle Drive, Suite 100 P.O. Box 110605 RTP, NC 27709  Chairperson- Glenn C. Veit |
| Northeast Georgia Research Center, LLC 520 Jesse Jewell Parkway Gainesville, GA 30501 | Copernicus Group IRB One Triangle Drive, Suite 100 P.O. Box 110605 RTP, NC 27709  Chairperson- Glenn C. Veit |
| Southwest Allergy and Asthma Center, P.A. 7711 Louis Pasteur Ste 901/905 San Antonio, Texas 78229 | Copernicus Group IRB One Triangle Drive, Suite 100 P.O. Box 110605 RTP, NC 27709  Chairperson- Glenn C. Veit |
| Hill Country Family Medical Center 114 Trade Ave. Boerne, TX 78006 | Copernicus Group IRB One Triangle Drive, Suite 100 P.O. Box 110605 RTP, NC 27709  Chairperson- Glenn C. Veit |
| Rameshwar N. Mathur, MD, PA 6250 N. US 1, Cocoa, Florida 32927 | Copernicus Group IRB One Triangle Drive, Suite 100 P.O. Box 110605 RTP, NC 27709  Chairperson- Glenn C. Veit |
| Allergy & Asthma care Center of So. Calif. 3816 Woodruff Ave.#209 Long Beach, CA 90808 | Copernicus Group IRB One Triangle Drive, Suite 100 P.O. Box 110605 RTP, NC 27709  Chairperson- Glenn C. Veit |
| University of Kentucky, Division of Allergy/ Immunology, 135 E Maxwell Street, Suite 250, Lexington, KY 40508 | Office of Research Integrity, 315 Kinkead Hall, University of Kentucky, Lexington, KY 40506-0057  Chairperson- Thomas Foster |
| Nathan E. Nachlas, MD, 1601 Clint Moore Road, Boca Raton, FL 33487 | Copernicus Group IRB One Triangle Drive, Suite 100 P.O. Box 110605 RTP, NC 27709  Chairperson- Glenn C. Veit |
| Children’s Lung Specialists, 3820 Meadows Lane, Las Vegas, NV. 89107 | Copernicus Group IRB One Triangle Drive, Suite 100 P.O. Box 110605 RTP, NC 27709  Chairperson- Glenn C. Veit |
| Integrated Research Group Inc., 4646 Brockton Avenue, Suites 101/201/202/203/302, Riverside, CA 92506 | Copernicus Group IRB One Triangle Drive, Suite 100 P.O. Box 110605 RTP, NC 27709  Chairperson- Glenn C. Veit |
| Princeton Center for Clinical Research, Montgomery Professional Center, 24 Vreeland Drive, Skillman, NJ 08558 | Copernicus Group IRB One Triangle Drive, Suite 100 P.O. Box 110605 RTP, NC 27709  Chairperson- Glenn C. Veit |
| Allergy Medical Group of the North Area, 935 Reserve Drive, Roseville, CA 95678-1340 | Copernicus Group IRB One Triangle Drive, Suite 100 P.O. Box 110605 RTP, NC 27709  Chairperson- Glenn C. Veit |
| Pediatric Care Medical Group, Inc., 17822 Beach Blvd., Suite 278/400, Huntington Beach, CA 92647 | Copernicus Group IRB One Triangle Drive, Suite 100 P.O. Box 110605 RTP, NC 27709  Chairperson- Glenn C. Veit |
| Montefiore Medical Center, Blondell Avenue Suite 220, Bronx, New York 10461 | Biomedical Research Alliance of New York, LLC, Institutional Review Board, 225 Community Drive, Suite 100, Great Neck, New York 11021  Co. Chairperson- Keith Krasinski  Co. Chairperson- Mark Sinnett |
| Montana Allergy & Asthma Specialists, 2900 12^th^ Avenue North, Suite 302E, Billings MT 59101 | Copernicus Group IRB, One Triangle Drive, Suite 100, Po Box 110605, RTP, NC 27709  Chairperson- Glenn C. Veit |
| David L. Schneider, MD, APMC, 3225 Danny Park, Ste 100, Metairie, LA 70002 | Copernicus Group IRB One Triangle Drive, Suite 100 P.O. Box 110605 RTP, NC 27709  Chairperson- Glenn C. Veit |
| Montana Medical Research, Inc., 2687 Palmer St, Suite E, Missoula, MT 59808 | Copernicus Group IRB One Triangle Drive, Suite 100 P.O. Box 110605 RTP, NC 27709  Chairperson- Glenn C. Veit |
| Peninsula Pulmonary Medical Associates, 23550 Hawthorne Blvd., Suite 180, Torrance, CA 90505 | Copernicus Group IRB One Triangle Drive, Suite 100 P.O. Box 110605 RTP, NC 27709  Chairperson- Glenn C. Veit |
| California Allergy &Asthma Group, 41230 11^th^ Street West Suite A, Palmdale, CA 93551 | Copernicus Group IRB One Triangle Drive, Suite 100 P.O. Box 110605 RTP, NC 27709  Chairperson- Glenn C. Veit |
| Allergy, Asthma & Clinical Research Center, Martha M. Tarpay, MD, 4200 W. Memorial Road, Suite 206, Oklahoma City, OK 73120 | Copernicus Group IRB One TrianAgle Drive, Suite 100 P.O. Box 110605 RTP, NC 27709  Chairperson- Glenn C. Veit |
| Clinical Research of The Ozarks, Inc., 509 East 10^th^ Street, Rolla, MO 65401 | Copernicus Group IRB One Triangle Drive, Suite 100 P.O. Box 110605 RTP, NC 27709  Chairperson- Glenn C. Veit |
| Abraham Research, PLLC, 747 Buttermilk Pike, Suite 2, Crescent Springs, KY 41017 | Copernicus Group IRB One Triangle Drive, Suite 100 P.O. Box 110605 RTP, NC 27709  Chairperson- Glenn C. Veit |
| Southern California Institute for Respiratory Disease, Inc., Cedars-Sinai Medical Towers, 8635 West Third Street, Suite 965W, Los Angeles, CA 90048 | Copernicus Group IRB One Triangle Drive, Suite 100 P.O. Box 110605 RTP, NC 27709  Chairperson- Glenn C. Veit |
| Steven F. Weinstein, MD, Allergy & Asthma Specialists Medical Group, 17742 Beach Blvd., Suite 310/340, Huntington Beach, CA 92647 | Copernicus Group IRB One Triangle Drive, Suite 100 P.O. Box 110605 RTP, NC 27709  Chairperson- Glenn C. Veit |

**Study FFA109687: 108 Centres**

| **FFA109687**  **Hospital/ Institution and Address** | **FFA109687 IEC/IRB Committee Chair and Name of Committee** |
| --- | --- |
| **BULGARIA** |  |
| Dispensary of Pulmonary Diseases, 78 St. St. Kiril i Metodii Street, 1202 Sofia Bulgaria | Ethics Committee for Multicenter Trials 26 Yanko Sakazov St. 1504 Sofia, Bulgaria  Chairperson: Dencho Osmanliev |
| MHAT “Sveti Georgi”, 15ª Vassil Aprilov St, 4000 Plovdiv Bulgaria | Ethics Committee for Multicenter Trials 26 Yanko Sakazov St. 1504 Sofia, Bulgaria  Local Ethics Committee at MHAT “Sveti Georgi”, 15ª Vassil Aprilov St, 4000 Plovdiv Bulgaria  Chairperson: Boyko Milev |
| MHAT “Sveta Marina’, 1 Hristo Smirnenski St., 9010 Varna Bulgaria | Ethics Committee for Multicenter Trials 26 Yanko Sakazov St. 1504 Sofia, Bulgaria  Chairperson Assoc.Prof. Dencho Osmanliev  Local Ethics Committee at MHAT “Sveta Marina’, 1 Hristo Smirnenski St., 9010 Varna Bulgaria  Chairperson : Valentin Akabaliev |
| CCB SAl Ministry of Interior, 79 General Skobelev blvd., 1606 Sofia Bulgaria | Ethics Committee for Multicenter Trials 26 Yanko Sakazov St. 1504 Sofia, Bulgaria  Chairperson: Dencho Osmanliev  Local Ethics Committee at CCB SAl Ministry of Interior, 79 General Skobelev blvd., 1606 Sofia Bulgaria  Chairperson: Dimitar Raev |
| **CANADA** |  |
| Aggarwal & Associates Ltd, 40 Finchgate Blvd., Suite 314, Brampton ON L6T 3J1 Canada | Optimum Clinical Research Inc., 231 King Street East, Oshawa ON L1H 1C5 Canada  Chairperson: Bill Wilson |
| Credit Valley Professional Building, 2300 Elinton Ave W, Suite 511, Mississauga ONL5M2V8, Canada | Optimum Clinical Research Inc, 231 King Street East, Oshawa ONL1H1C5, Canada  Chairperson: Bill Wilson |
| Office of Kenneth Buttoo, 601 Harwood Avenue South, Suite 201, Ajax ON L1S 2J5 Canada | Optimum Clinical Research Inc., 231 King Street East, shawa ON L1H 1C5 Canada  Chairperson: Bill Wilson |
| Diex Research, 731 Galit Quest, Suite 120, Sherbrooke QC J1H 1Z1 Canada | Optimum Clinical Research Inc., 231 King Street East, Oshawa ON L1H 1C5 Canada  Chairperson: Bill Wilson |
| Centre de Recherche Appliquee en Allergie de Quebee, 2590 boulevard Laurier, 2e etage, bureau 225, Quebee City QC G1V 4M6 Canada | Optimum Clinical Research Inc., 231 King Street East, Oshawa ON L1H 1C5 Canada  Chairperson: Bill Wilson |
| C.I.C. Mauricie Inc,700 Boul Thibeau, Suite 230, Trois Rivières QC G8T 7A1 Canada | Optimum Clinical Research Inc., 231 King Street East, Oshawa ON L1H 1C5 Canada  Chairperson: Bill Wilson |
| Centre de Pneumologie, Pavillon U ler etage local 1753, 2725 Ch Ste-Foy, Quebee QC G1V 4G5 Canada | Comite d ethique de la recherché de 1 hopital Laval 2725 Ch Ste-Foy, Quebee QC G1V 4G5 Canada  Chairperson: Franck Molin |
| Canadian Phase Onward, Inc, 4646 Dufferin St, Unit 5, Toronto ON M3H 5S4 Canada | Optimum Clinical Research Inc., 231 King Street East, Oshawa ON L1H 1C5 Canada  Chairperson: Bill Wilson |
| Dr. Calvin Powell Professional Medical Corporation, 286 Conception Bay Hwy, P.O. Box 1017, Bay Roberts NL A0A 1G0 Canada | Optimum Clinical Research Inc., 231 King Street East, Oshawa ON L1H 1C5 Canada  Chairperson: Franck Molin |
| Allergy & Asthma Research Centre, 707-1081 Carling Ave, Ottawa ON K1Y 4G2 Canada | Optimum Clinical Research Inc., 231 King Street East, Oshawa ON L1H 1C5 Canada  Chairperson: Bill Wilson |
| **ESTONIA** |  |
| Tallinn Children’s Hospital, Tervise 28, Tallinn 13419, Estonia | Ethics Review Committee (ERC) on Human Research of the University Of Tartu, Ulikooli str 18, Tartu, 50090, Estonia  Chairperson: Andres Soosaar |
| Tartu University Clinics, Children’s Clinic, Lunini 6, Tartu 51014, Estonia | Ethics Review Committee (ERC) on Human Research of the University Of Tartu, Ulikooli str 18, Tartu, 50090, Estonia  Chairperson: Andres Soosaar |
| **FRANCE** |  |
| Groupe Sante Recherche, 35 rue de Ventadour, 31300 Toulouse, France | CPP Sud-Mediterranee IV, Hospital Saint-Eloi, 34295 Montpellier cedex 5, France  Chairperson: Sylvie HANSEL - ESTELLER |
| Cabinet Medical, 16 quai des Eaux Minerales, 42600 Montbrison, France | CPP Sud-Mediterranee IV, Hospital Saint-Eloi, 34295 Montpellier cedex 5, France  Chairperson: Sylvie HANSEL - ESTELLER |
| Hospital Arnaud de Villeneuve, Maladies Respiratoires, 371 avenue du Doyen Giraud, 34295 Montpellier, France | CPP Sud-Mediterranee IV, Hospital Saint-Eloi, 34295 Montpellier cedex 5, France  Chairperson: Sylvie HANSEL - ESTELLER |
| 200 rue Jean Jaures, 59690 Vieux Conde, France | CPP Sud-Mediterranee IV, Hospital Saint-Eloi, 34295 Montpellier cedex 5, France  Chairperson: Sylvie HANSEL - ESTELLER |
| 23 boulevard de Strasbourg, 83000Toulon, France | CPP Sud-Mediterranee IV, Hospital Saint-Eloi, 34295 Montpellier cedex 5, France  Chairperson: Sylvie HANSEL - ESTELLER |
| 1 rue jardin Bontanique, 27000 Evreux, France | CPP Sud-Mediterranee IV, Hospital Saint-Eloi, 34295 Montpellier cedex 5, France  Chairperson: Sylvie HANSEL - ESTELLER |
| **GERMANY** |  |
| Praxis Dr. med. Schuermann, Bahnhofstr. 30, 35037 Marburg, Germany | Ethik- Kommission bei der, Landesaerztekammer Hessen, Im Vogelsgesang 3, 60488 Frankfurt, Germany  Chairperson: Dr. A. Helberg-Lubinski |
| Praxis Dr. med. Steinhauser, Hauptstr. 71, 74889 Sinsheim, Germany | Ethik-Kommission der Landesaerztekammer Baden-Wuerttemberg, Jahnstrasse 38 A, 70597 Stuttgart  Chairperson: Dr. med. Georg Hook  Ethik- Kommission bei der, Landesaerztekammer Hessen, Im Vogelsgesang 3, 60488 Frankfurt, Germany  Chairperson: A. Helberg-Lubinski |
| Praxis Dr. Med von Versen, Yorckstr. 71, 10965 Berlin, Germany | Landesamt für Gesundheit und Soziales in Berlin, Geschaeftstelle der Ethik-Kommission Berlin, Saechsische Strasse 28, 10707 Berlin  Chairperson: PD Dr. Hans-Herbert Fülle  Ethik- Kommission bei der, Landesaerztekammer Hessen, Im Vogelsgesang 3, 60488 Frankfurt, Germany  Chairperson: A. Helberg-Lubinski |
| **KOREA** |  |
| Chonnam National University Hospital, 8 Hakdong, Dongku Gwangju 501-757 Republic of Korea | Chonnam National University Hospital Institutional Review Board, 8 Hakdong, Dongku Gwangju 501-757 Republic of Korea  Chairperson: Young-Ho Won |
| Chungbuk National University Hospital  410 Gasesin-dong, Heungdeok-gu, Cheongju, Chungcheongbuk-do (361-711), Korea | Chungbuk National University Hospital Institutional Review Board, 410 Gasesin-dong, Heungdeok-gu, Cheongju, Chungcheongbuk-do (361-711), Korea  Chairperson: See Ok Shin |
| Division of Allergy and Pulmonology, Soon Chun Hyang University Bu Cheon Hospital, 1174 Jung-Dong, Wonmi-Gu, Bucheon-Si, Gyeonggi-Do, 420-021, Korea | Institutional Review Board (IRB), Soon Chun Hyang University Bu Cheon Hospital, 1174 Jung-Dong, Wonmi-Gu, Bucheon-Si, Gyeonggi-Do, 420-021, Korea  Chairperson: Seung Duk Hwang |
| Ajou University Hospital, San 5, Wonchon-dong, Yeongtong-gu Suwon 443-721 Korea | Ajou University Hospital Regional Clinical Trial Center, San 5, Wonchon-dong, Yeongtong-gu Suwon 443-721 Korea  Chairperson: Mi Son Chun |
| **MEXICO** |  |
| Torre Italis Avonida Copernico 3817 consultorio 14 esquina Sagitario Colonia Arboledas Zapopan, Jalisco C. P. 45040 Mexico | Comité de Etica del Hospital Real: San José Av. Lázaro Cárdenas 4149 Colonia Jardines de San Ignacio Zapopan, Jalisco C.P. 45040 México  Chairperson- Joaquín Jiménez Flor |
| Instituto Jalisciense de Investigación Clínica, S.A. de C.V. Penitenciaria 20 Colonia Centro Guadalajara Jalisco C.P. 44100 México | Comité de Etica del Instituto Jalisciense de Investigación Clínica, S.A. de C.V.: Penitenciaria 20 Colonia Centro Guadalajara, Jalisco C.P. 44100, México  Chairperson- Luz María Coronado-So |
| **PERU** |  |
| Clinica Anglo Americana, Av. Alfredo Salazar 314, Lima 27- Peru | Comite de Etica Asociacion Benefica PRISMA, Carlos Gonzales No 251 Urb. Maranga, Lima 32-Peru  Chairperson: Dr. Salomón Zavala Sarrio |
| Clinica Internacional, Av. Garcilazo de la Vega 1420, Lima 1-Peru | Comite de Etica PRISMA, Carlos Gonzales No 251 Urb. Maranga, Lima 32-Peru  Chairperson: Salomón Zavala Sarrio |
| Clinica Ricardo Palma, Av. Javier Prado Este 1066., Lima 27- Peru | Comite de Etica PRISMA, Carlos Gonzales No 251 Urb. Maranga. Lima 32- Perk  Chairperson: Salomón Zavala Sarrio |
| **PHILIPPINES** |  |
| Pulmonary Section, Medicine Department, UP-Philippine General Hospital, Taft Avenue, Manila 1000, Philippines | Research Implementation and Development Office, College of Medicine – Philippine General Hospital, 547 Pedro Gil Street, Ermita Manila 1000, Philippines  Chairperson: Renato B. Dantes |
| Rm 302 Cebu Doctors College Admin Bldg., Kamuning St. Cebu City 6000, Philippines | Cebu Doctors University – Cebu Doctors University Hospital, lnsitutional Evaluation and Review Committee, Mandaue City, Cebu 6000, Philippines  Chairperson: Letecia Cabrera |
| Rrn 204 Cebu Doctors Hospital, Osmena Boulevard Cebu City, 6000, Philippines | Cebu Doctors University – Cebu Doctors University Hospital, lnsitutional Evaluation and Review Committee, Mandaue City, Cebu 6000, Philippines  Chairperson: Letecia Cabrera |
| De La Salle University Health Sciences Campus, The Angelo King Medical Research Center, Congressional Road, Dasmarinas Cavite 41 14, Philippines | De La Salle University Health Sciences Campus, Institutional and Ethical Review Board, The Angelo King Medical Research Center, Congressional Road, Dasmarinas Cavite 41 14, Philippines  Chairperson: Rev. Fr. Danilo C. Tiong |
| **POLAND** |  |
| Centrum Medyczne Lucyna Andrzej Dymek NZOZ s.c. Ul. Warynskiego 4, 47-120 Zawadzkie, Poland | Komisja Bioetyki Uniwersytetu Medycznego w Lodzi, Ul. Kosciuszki 4, 90-419 Lodz, Poland  Chairperson: Przedzisław Polakowski |
| Uniwersytecki Szpital Kliniczny nr 1 im. Norberta Barlickiego w Lozi, Ui. Kopcinskiego 22, 90-153 Lodz, Poland | Komisja Bioetyki Uniwersytetu Medycznego w Lodzi, Kosciuszki 4, 90-419 Lodz, Poland  Chairperson: Przedzisław Polakowski |
| Uniwersytecki Szpital Kliniczny nr 1 im. Norberta Barlickiego w Lozi, Ui. Kopcinskiego 22, 90-153 Lodz, Poland | Komisja Bioetyki Uniwersytetu Medycznego w Lodzi, Kosciuszki 4, 90-419 Lodz, Poland  Chairperson: Przedzisław Polakowski |
| **RUSSIAN FEDERATION** |  |
| City Clinical Hiospital *#5,* 31 Dimitrova str., 654063, Novokuznetsk, Russia | Ethics Committee within Federal Service on Surveillance in Healthcare and Social Development of Russian Federation, Russia, 109074, Moscow, Slavyanskaya pl., 4, Bldn. 1  Independent Ethics Committee attached to the Municipal Healthcare Institution “Children Clinical Hospitaln” #4 33 Dimitrova str., 654063, Novokuznetsk, Russia  Chairperson: Olga V. Domanskaya |
| Municipal Hospital #5, Zmelnogorskly trakt, 75, 656045, Bamual, Russia | Ethics Committee within Federal Service on Surveillance in Healthcare and Social Development of Russian Federation, Russia, 109074, Moscow, Slavyanskaya pl., 4, Bldn. 1  Independent Ethics Committee attached to the Municipal Hospital #5, Zmelnogorskly trakt, 75, 656045, Bamual, Russia  Chairperson: Svetlana V. Kovchina |
| State Institution for Higher Professional Education “Samara State Medical University” of Roszdrav, Chair of Basic and Clinical Microbiology, Immunology and Allergology, Gagarina str., 18, 443099, Samara, Russia | Ethics Committee within Federal Service on Surveillance in Healthcare and Social Development of Russian Federation, Russia, 109074, Moscow, Slavyanskaya pl., 4, Bldn. 1  Independent Ethics Committee attached to the State Institution of Healthcare Samara Regional Clinical Hospital for War Veterans, ul. XXII Partsjezda, 43, 443063, Samara, Russia  Chairperson: Igor G. Leshchenko |
| GOU VPO Smolensk State Medical Academy of Roszdrav, Center of Clinical Trials of Diagnostic and Medicinal Agents (28, ul. Krupskoy, Smolensk, 214019, Russia; 27, Gagarina prospect, Smolensk, 214018, Russia) | Ethics Committee within Federal Service on Surveillance in Healthcare and Social Development of Russian Federation, Russia, 109074, Moscow, Slavyanskaya pl., 4, Bldn. 1  Independent Ethics Committee attached to the State Educational Institute of higher Professional Education “Smolensk State Medical Academy of Federal Agency of Healthcare and Social Development”, 28, ul. Krupskoy, Smolensk, 214019, Russia  Chairperson: Alexander V. Litvinov |
| **SLOVAKIA** |  |
| Dept. Paediatrics Faculty Hospital Martin, Kollarova 2, 036 59 Martin,Slovakia Republic | Eticka komisia of Faculty Hospital Martin, Kollarova 2, 036 59 Martin, Slovakia Republic  Chairperson: Zelmira Fetisovova |
| Centrum imunologie a alergologie, S.R.O. Pavla Horova 14, 841 08 Bratislava, Slovakia | Eticka komisia Bratislavskeho samospravneho kraja, Sabinovska 16, 82 05 Bratislava, Slovakia  Chairperson: Olga Vesela |
| **SWEDEN** |  |
| OLIN-projektet Boden, Björknäs vårdcentral, Idrottsgatan 3, SE-961 64 Boden, Sweden | Regionala etikprovningsnamnden I Umea, Samverkanshuset, Universitetsomradet, SE-90187 Umea, Sweden  Chairperson: Anders Iacobæus |
| OLIN-projektet Lulea, Stadsvikens vårdcentral, Robertsviksgatan 9, SE-971 89 LULEÅ, Sweden | Regionala etikprovningsnamnden I Umea, Samverkanshuset, Universitetsomradet, SE-90187 Umea, Sweden  Chairperson: Anders Iacobæus |
| **UNITED STATES** |  |
| Allergy Asthma Research Institute 333 Londonderry Drive Suite 110 Waco, TX76712 | Copernicus Group IRB One Triangle Drive Suite 100 PO Box 110605 RTP, NC 27709  Chairperson: Glenn C. Veit, JD |
| Live Oak Allergy & Asthma Clinic 11515 Toepperwein Rd., Suite 202 Live Oak, TX 78233 | Copernicus Group IRB One Triangle Drive Suite 100 PO Box 110605 RTP, NC 27709  Chairperson: Glenn C. Veit, JD |
| Quality Assurance Research Center 343 W. Houston, Suite 802 San Antonio, Texas 78205 | Copernicus Group IRB One Triangle Drive Suite 100 PO Box 110605 RTP, NC 27709  Chairperson: Glenn C. Veit, JD |
| Bernstein Clinical Research Center 3444 Winton Road Cincinnati, Ohio 45231 | Copernicus Group IRB One Triangle Drive Suite 100 PO Box 110605 RTP, NC 27709  Chairperson: Glenn C. Veit, JD |
| Iowa Clinical Research Corporation 225 East Burlington Street Iowa City, Iowa 52240 | Copernicus Group IRB One Triangle Drive Suite 100 PO Box 110605 RTP, NC 27709  Chairperson: Glenn C. Veit, JD |
| Texas Pulmonary and Critical Care Consultants, P. A. Research Center 909 Eighth Avenue Fort Worth, Texas 76104 | Copernicus Group IRB One Triangle Drive Suite 100 PO Box 110605 RTP, NC 27709  Chairperson: Glenn C. Veit, JD |
| Mayo Clinic, 200 First Street SW, Rochester, MN 55905 | Mayo Institutional Review Board, Mayo Clinic, 200 First Street SW, Rochester, MN 55905  Chairperson: Bart L. Clark, MD |
| East Tennessee Center for Clinical Research 801 Weisgarber Road, Suite 200 Knoxville, TN 37909 | Copernicus Group IRB One Triangle Drive Suite 100 PO Box 110605 RTP, NC 27709  Chairperson: Glenn C. Veit, JD |
| AAAL Cenkr, 709 North Clyde Morris, Daytone Beach, Florida 32114 | Copernicus Group IRB One Triangle Drive Suite 100 PO Box 110605 RTP, NC 27709  Chairperson: Glenn C. Veit, JD |
| Allergy and Asthma Research Center, P. A. 2414 Babcock Road, Suite 108-109 San Antonio, TX 78229 | Copernicus Group IRB One Triangle Drive Suite 100 PO Box 110605 RTP, NC 27709  Chairperson: Glenn C. Veit, JD |
| William L. Ebbeling 1646 E. Herndon Ave, Suite 106 Fresno, CA 93720 | Copernicus Group IRB One Triangle Drive Suite 100 PO Box 110605 RTP, NC 27709  Chairperson: Glenn C. Veit, JD |
| Bellingham Asthma, Allergy & Immunology Clinic 3015 Squalicum Pkwy #180 Bellingham, WA 98225 | Copernicus Group IRB One Triangle Drive Suite 100 PO Box 110605 RTP, NC 27709  Chairperson: Glenn C. Veit, JD |
| Central Florida Research Institute, LLC 1788 West Fairbanks Ave, Suite B. Winter Park, Florida 32789 | Copernicus Group IRB One Triangle Drive Suite 100 PO Box 110605 RTP, NC 27709  Chairperson: Glenn C. Veit, JD |
| Montana Allergy & Asthma Specialists 2900 12th Avenue North, Suite 302E Billings, MT 59101 | Copernicus Group IRB One Triangle Drive Suite 100 PO Box 110605 RTP, NC 27709  Chairperson: Glenn C. Veit, JD |
| College Park Family Care Center Multi-Speciality Clinical Research 12208 W. 87^th^ Street, Suite 180 Lenexa, KS 6621 | Copernicus Group IRB One Triangle Drive Suite 100 PO Box 110605 RTP, NC 27709  Chairperson: Glenn C. Veit, JD |
| Allergy & Respiratory Center 4048 Dressler Road NW Canton, Ohio 44718 | Copernicus Group IRB One Triangle Drive Suite 100 PO Box 110605 RTP, NC 27709  Chairperson: Glenn C. Veit, JD |
| Island Medical Research, P.C. 242 Merrick Road, Suite 401 Rockville Center, NY 11570 | Copernicus Group IRB One Triangle Drive Suite 100 PO Box 110605 RTP, NC 27709  Chairperson: Glenn C. Veit, JD |
| Ram S. Goswami , PC 12701 South Telegraph Suite 103 Taylor, MI 48180 | Copernicus Group IRB One Triangle Drive Suite 100 PO Box 110605 RTP, NC 27709  Chairperson: Glenn C. Veit, JD |
| Northen Illinois Research Associates 625 E. Bethany Road Suite 3 DeKalb, IL 60115 | Copernicus Group IRB One Triangle Drive Suite 100 PO Box 110605 RTP, NC 27709  Chairperson: Glenn C. Veit, JD |
| Mississippi Asthma & Allergy Clinic, P.A. 1600 North State Street Suites 101 & 201 Jackson, MS 39202 | Copernicus Group IRB One Triangle Drive Suite 100 PO Box 110605 RTP, NC 27709  Chairperson: Glenn C. Veit, JD |
| West Coast Clinical Trials Phase 2-4, LLC 2600 Redondo Avenue 4^th^ Floor, Suite #401 Long Beac, CA 90806 | Copernicus Group IRB One Triangle Drive Suite 100 PO Box 110605 RTP, NC 27709  Chairperson: Glenn C. Veit, JD |
| North Texas Family Medicine 4001 W. 15^th^ Street, Suite 445 Plano, TX 75093 | Copernicus Group IRB One Triangle Drive Suite 100 PO Box 110605 RTP, NC 27709  Chairperson: Glenn C. Veit, JD |
| Allergy & Asthma Research Group 1488 Oak Street Eugene, OR 97401-403 | Copernicus Group IRB One Triangle Drive Suite 100 PO Box 110605 RTP, NC 27709  Chairperson: Glenn C. Veit, JD |
| Timber Lane Allergy & Asthma Research, LLC 50 Timber Lane South Burlington, VT 05403 | Copernicus Group IRB One Triangle Drive Suite 100 PO Box 110605 RTP, NC 27709  Chairperson: Glenn C. Veit, JD |
| Clinical Research Institute of Southern Oregon, PC 3860 Crater Lake Avenue, Suite B Medford, OR 97504 | Copernicus Group IRB One Triangle Drive Suite 100 PO Box 110605 RTP, NC 27709  Chairperson: Glenn C. Veit, JD |
| UCLA David Geffen School of Medicine 108331 LeConte Avenue 37-131 CHS Los Angeles, CA 90095 | UCLA Office for Protection of Research Subjects Medical Institutional Review Board 11000 Kinross Avenue, Suite 102 Los Angeles, CA 90095  Chairperson: Glenn C. Veit, JD |
| The Clinical Research Center, L.L.C. 1040 North Mason Road, Suite 112 St. Louis, Missouri 63141 | Copernicus Group IRB One Triangle Drive Suite 100 PO Box 110605 RTP, NC 27709  Chairperson: Glenn C. Veit, JD |
| North Carolina Clinical Research 2615 Lake Drive, Suite 301 Raleigh, NC 27607 | Copernicus Group IRB One Triangle Drive Suite 100 PO Box 110605 RTP, NC 27709  Chairperson: Glenn C. Veit, JD |
| Clinical Research of the Ozarks, Inc. 407 A East Russell Avenue, Suite 3 Warrensburg, MO 64093 | Copernicus Group IRB One Triangle Drive Suite 100 PO Box 110605 RTP, NC 27709  Chairperson: Glenn C. Veit, JD |
| Rocky Mt Center for Clinical Research 8550 West 38^th^ Ave., #202 Wheat Ridge, CO 80033 | Copernicus Group IRB One Triangle Drive Suite 100 PO Box 110605 RTP, NC 27709  Chairperson: Glenn C. Veit, JD |
| Jane J. Lee M.D., P.A. Research Center 411 N. Woshington Ave. Suite 2400 Dallas, Texas 75246 | Copernicus Group IRB One Triangle Drive Suite 100 PO Box 110605 RTP, NC 27709  Chairperson: Glenn C. Veit, JD |
| Respiratory Medicine Research Institute of Michigan, PLC 5333 McAuley Drive, Suite R-1018 Ypsilanti MI 48197 | St. Joseph Mercy Health System Clinical Research Committee 5301 East Huron River Drive P.O. Box 995 Ann Arbor, MI 48106-0995  Chairperson**:** James Mitchiner, MD |
| University of South Florida Asthama, Allergy and Immunology Clinical Research Unit, 13801 Bruce B. Downs Blvd., Suite 505, Tampa, FL 33613 | Western IRB, 3535 Seventh Ave SW, Olympia, WA 98502-5010  Chairman: Theodore D. Schultz |
| San Diego Sports Medicine and Family Health Center 6699 Alvarado Rd. Suite 2100 San Diego Ca. 92120 | Copernicus Group IRB One Triangle Drive Suite 100 PO Box 110605 RTP, NC 27709  Chairperson: Glenn C. Veit, JD |
| AARA Research Center 9900 North Central Expressway, Suite 555 Dakas, Texas 75231 | Copernicus Group IRB One Triangle Drive Suite 100 PO Box 110605 RTP, NC 27709  Chairperson: Glenn C. Veit, JD |
| Northeast Georgia Research Center, LLC 520 Jesse Jewell Parkway Gainesville | Copernicus Group IRB One Triangle Drive Suite 100 PO Box 110605 RTP, NC 27709  Chairperson: Glenn C. Veit, JD |
| Southwest Allergy and Asthma Center, P.A. 7711 Louis Pasteur Ste 901/905 San Antonio, Texas 78229 | Copernicus Group IRB One Triangle Drive Suite 100 PO Box 110605 RTP, NC 27709  Chairperson: Glenn C. Veit, JD |
| Hill Country Family Medical Center 114 Trade Ave, Boerne, TX 78006 | Copernicus Group IRB One Triangle Drive Suite 100 PO Box 110605 RTP, NC 27709  Chairperson: Glenn C. Veit, JD |
| Rameshwar N. Mathur 6250 N. US 1 Cocoa, Florida 32927 | Copernicus Group IRB One Triangle Drive Suite 100 PO Box 110605 RTP, NC 27709  Chairperson: Glenn C. Veit, JD |
| Allergy & Asthma Care Center of So. Calif. 3816 Woodruff Ave. #209 Long Beach, CA 90808 | Copernicus Group IRB One Triangle Drive Suite 100 PO Box 110605 RTP, NC 27709  Chairperson: Glenn C. Veit, JD |
| Lowcountry Medical Care 167- C Bluffton Rd. Bluffton S. C. 29910 | Copernicus Group IRB One Triangle Drive Suite 100 PO Box 110605 RTP, NC 27709  Chairperson: Glenn C. Veit, JD |
| PharmQuest 806 Green Valley Road, Ste 305 Greeensborn, NC 27408 | Copernicus Group IRB One Triangle Drive Suite 100 PO Box 110605 RTP, NC 27709  Chairperson: Glenn C. Veit, JD |
| Children’s Lung Specialists 3820 Meadows Lane Las Vegas, NV. 89107 | Copernicus Group IRB One Triangle Drive Suite 100 PO Box 110605 RTP, NC 27709  Chairperson: Glenn C. Veit, JD |
| Clayton Sleep Institute 2531 South Big Bend Blvd St. Louis MO 63143 | Copernicus Group IRB One Triangle Drive Suite 100 PO Box 110605 RTP, NC 27709  Chairperson: Glenn C. Veit, JD |
| Integrated Research Group, Inc. 4646 Brockton Avenue Suites 101/ 201/ 202 /203 / 302 Riverside, CA 92506 | Copernicus Group IRB One Triangle Drive Suite 100 PO Box 110605 RTP, NC 27709  Chairperson: Glenn C. Veit, JD |
| Princeton Center for Clinical Research Montgornery Professional Center 24 Vreeland Drive Skillman, NJ 08558 | Copernicus Group IRB One Triangle Drive Suite 100 PO Box 110605 RTP, NC 27709  Chairperson: Glenn C. Veit, JD |
| Allergy Medical Group of the North Area 935 Reserve Drive Roseville, CA 95678-1340 | Copernicus Group IRB One Triangle Drive Suite 100 PO Box 110605 RTP, NC 27709  Chairperson: Glenn C. Veit, JD |
| Pediatric Care Medical Group, Inc. 178200 Beach Blvd., Suite 278/400 Huntington Beach, CA 92647 | Copernicus Group IRB One Triangle Drive Suite 100 PO Box 110605 RTP, NC 27709  Chairperson: Glenn C. Veit, JD |
| National Allergy, Asthma & Urticaria Centers of Charleston, P.A. 9165 University Blvd. Charleston, SC 29406 | Copernicus Group IRB One Triangle Drive Suite 100 PO Box 110605 RTP, NC 27709  Chairperson: Glenn C. Veit, JD |
| Montana Medical Research, Inc. 2687 palmer St., Suite E. Missoula, MT 59808 | Copernicus Group IRB One Triangle Drive Suite 100 PO Box 110605 RTP, NC 27709  Chairperson: Glenn C. Veit, JD |
| Paul A. Shapero 700 ML Hope Avenue, Suite 430 Bangor, ME 04401 | Copernicus Group IRB One Triangle Drive Suite 100 PO Box 110605 RTP, NC 27709  Chairperson: Glenn C. Veit, JD |
| Peninsula Pulmonary Medical Associates 23550 Hawthorne Blvd., Suite 180 Torrance, CA 90505 | Copernicus Group IRB One Triangle Drive Suite 100 PO Box 110605 RTP, NC 27709  Chairperson: Glenn C. Veit, JD |
| Center for Clinical Trials of San Gabriel, 1300 S. Sunset Ave, Suite 101, West Covina, CA 91790 | Copernicus Group IRB One Triangle Drive Suite 100 PO Box 110605 RTP, NC 27709  Chairperson: Glenn C. Veit, JD |
| California Allergy & Asthma Medical Group 41230 11^th^ Street West Suite A Palmdate, CA. 93551 | Copernicus Group IRB One Triangle Drive Suite 100 PO Box 110605 RTP, NC 27709  Chairperson: Glenn C. Veit, JD |
| Allergy, Asthma & Clinical Research Center Martha M. Tarpay 4200 W. Memorial Road, Suite 206 Oklahoma City, OK 73120 | Copernicus Group IRB One Triangle Drive Suite 100 PO Box 110605 RTP, NC 27709  Chairperson: Glenn C. Veit, JD |
| Clinical Research of The Ozarks, Inc. 509 E. 10^th^ Street Rolla, MO 65401 | Copernicus Group IRB One Triangle Drive Suite 100 PO Box 110605 RTP, NC 27709  Chairperson: Glenn C. Veit, JD |
| Office of Julio H. Urena 1157 Main Avenue Clifton, NJ 07011 | Copernicus Group IRB One Triangle Drive Suite 100 PO Box 110605 RTP, NC 27709  Chairperson: Glenn C. Veit, JD |
| Southern California Institute for Respiratory Diseases, Inc. 8635 West Third Street Suite 965 W Los Angeles, CA 90048 | Copernicus Group IRB One Triangle Drive Suite 100 PO Box 110605 RTP, NC 27709  Chairperson: Glenn C. Veit, JD |
