# Supplementary material for: Ethnic sensitivity assessment of fluticasone furoate/vilanterol in East Asian asthma patients from randomized double-blind multicentre Phase IIb/III trials
Source: BMC Pulm Med. 2015 Dec 24;15:165. doi: 10.1186/s12890-015-0159-z (PMC4690330; doi:10.1186/s12890-015-0159-z)
Supplement: Additional file 4: — Adjusted treatment differences from baseline in trough FEV1 at Week 12 (Efficacy population). (DOCX 1.49 MB) [file 12890_2015_159_MOESM4_ESM.docx]

**Additional File 4 Adjusted treatment differences from baseline in trough FEV_1_ at Week 12 (Efficacy population)**


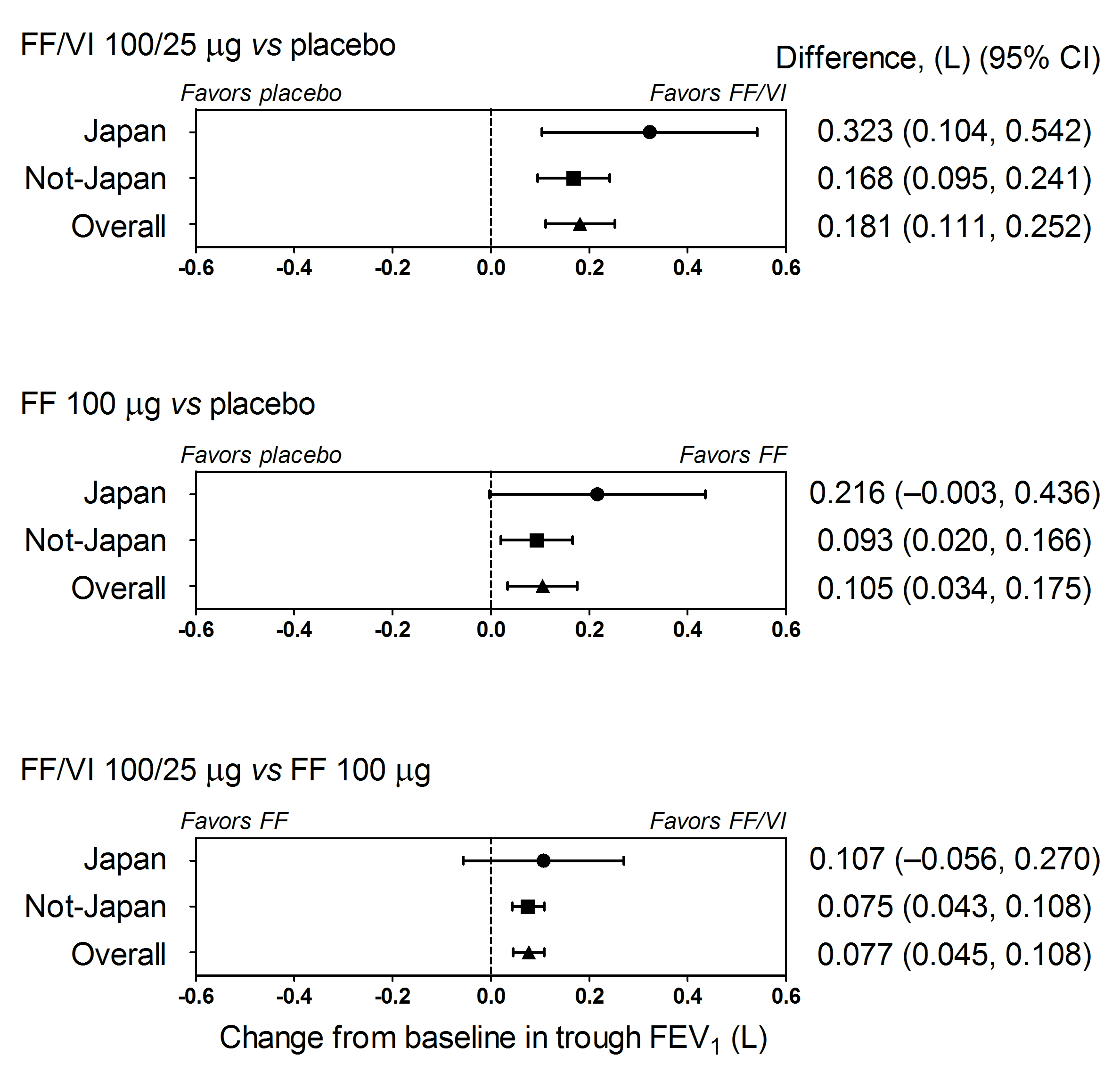


CI, confidence interval; FEV_1_, forced expiratory volume in one second; FF, fluticasone furoate; VI, vilanterol.

Efficacy population consists of data from studies HZA106827, HZA106829, and HZA106837. Data presented are difference in least squares mean change. Number of patients analyzed for a) FF/VI 100/25 μg OD: Japan N = 46, Not-Japan N = 1,155,
Overall N = 1,201; b) FF 100 μg OD: Japan N = 46, Not-Japan N = 1,157, Overall N = 1,203; c) placebo: Japan N = 18, Not-Japan N = 175, Overall N = 193.
