# Supplementary material for: Ethnic sensitivity assessment of fluticasone furoate/vilanterol in East Asian asthma patients from randomized double-blind multicentre Phase IIb/III trials
Source: BMC Pulm Med. 2015 Dec 24;15:165. doi: 10.1186/s12890-015-0159-z (PMC4690330; doi:10.1186/s12890-015-0159-z)
Supplement: Additional file 5: — Ease of use of the ELLIPTA® dry powder inhaler (DPI; ELLIPTA® is a trademark of the GlaxoSmithKline group of companies). (DOCX 22.5 KB) [file 12890_2015_159_MOESM5_ESM.docx]

**Additional File 5 Ease of use of the ELLIPTA^®^ dry powder inhaler (DPI; ELLIPTA^®^ is a trademark of the GlaxoSmithKline group of companies)**

Ease-of use of the ELLIPTA DPI was assessed at the end of 4 weeks of treatment in study HZA106827. Use of the inhaler was demonstrated to the patient up to three times at Week 0 until the patient could use a placebo demonstration inhaler correctly. The correct use of the placebo demonstration inhaler was reassessed after 2 and 4 weeks of treatment, with the entire procedure demonstrated again if the patient did not perform the maneuvers correctly. After 4 weeks of treatment, patients were asked to rate the inhaler by answering the following questions: (1) how do you rate the ease of use of the inhaler? (2) how easily are you able to tell how many doses of medication are left in the inhaler? For each of the questions, possible answers were: (1) very easy; (2) easy; (3) neutral; (4) difficult; (5) very difficult. The results of this questionnaire in the asthma patients in Japan are presented in Additional File 5.
