# Supplementary material for: Ethnic sensitivity assessment of fluticasone furoate/vilanterol in East Asian asthma patients from randomized double-blind multicentre Phase IIb/III trials
Source: BMC Pulm Med. 2015 Dec 24;15:165. doi: 10.1186/s12890-015-0159-z (PMC4690330; doi:10.1186/s12890-015-0159-z)
Supplement: Additional file 6: — Summary of ease of use assessment and responses to questions on the ELLIPTA DPI by patients from Japan (HZA106827 ITT population). (DOCX 25.2 KB) [file 12890_2015_159_MOESM6_ESM.docx]

**Additional File 6 Summary of ease of use assessment and responses to questions on the ELLIPTA DPI by patients from Japan (HZA106827 ITT population)**

|  | Placebo  N = 19 | FF  100 μg OD  N = 16 | FF/VI  100/25 μg OD  N = 15 | Total  N = 50 |
| --- | --- | --- | --- | --- |
| **Week 0** | | | | |
| Patients using inhaler correctly, n (%) | 19 (100) | 16 (100) | 15 (100) | 50 (100) |
| **Week 2** | | | | |
| Patients using inhaler correctly, n (%) | 17 (100)* | 16 (100) | 15 (100) | 48 (100)^†^ |
| **Week 4** | | | | |
| Patients using inhaler correctly, n (%) | 13 (100)^‡^ | 15 (100)^§^ | 14 (100)* | 42 (100)^¶^ |
| **How do you rate the ease of use of the inhaler?** | | | | |
| n | 14 | 15 | 14 | 43 |
| Very easy, n (%) | 8 (57) | 6 (40) | 10 (71) | 24 (56) |
| Easy, n (%) | 5 (36) | 2 (13) | 2 (14) | 9 (21) |
| Neutral, n (%) | 1 (7) | 6 (40) | 2 (14) | 9 (21) |
| Difficult, n (%) | 0 (0) | 1 (7) | 0 (0) | 1 (2) |
| Very difficult, n (%) | 0 (0) | 0 (0) | 0 (0) | 0 (0) |
| **How easily are you able to tell how many doses of medication are left in the inhaler?** | | | | |
| n | 14 | 15 | 14 | 43 |
| Very easy, n (%) | 12 (86) | 10 (67) | 12 (86) | 34 (79) |
| Easy, n (%) | 2 (14) | 4 (27) | 2 (14) | 8 (19) |
| Neutral, n (%) | 0 (0) | 0 (0) | 0 (0) | 0 (0) |
| Difficult, n (%) | 0 (0) | 1 (7) | 0 (0) | 1 (2) |
| Very difficult, n (%) | 0 (0) | 0 (0) | 0 (0) | 0 (0) |

DPI, dry powder inhaler; FF, fluticasone furoate; ITT, intent-to-treat; OD, once daily;
VI, vilanterol.

*N = 17; ^†^N = 48; ^‡^N = 13; ^§^N = 15; ^¶^N = 42.

The results of inhaler-use assessment indicate that 100% of patients in Japan were able to use the ELLIPTA DPI correctly on first usage (95% of Overall population). The DPI was rated as “easy/very easy” to use by 77% of patients in Japan (91% of Overall population) and 98% of patients in Japan reported it was “Easy/Very Easy” to determine how many doses remained (96% of Overall population). Whilst the sample size in the Japanese cohort is limited, the results align with data on ease of use of the ELLIPTA DPI from the Overall population of HZA106827.
