# Supplementary material for: Ethnic sensitivity assessment of fluticasone furoate/vilanterol in East Asian asthma patients from randomized double-blind multicentre Phase IIb/III trials
Source: BMC Pulm Med. 2015 Dec 24;15:165. doi: 10.1186/s12890-015-0159-z (PMC4690330; doi:10.1186/s12890-015-0159-z)
Supplement: Additional file 7: — Summary of exposure in each treatment arm (Safety population). (DOCX 26.5 KB) [file 12890_2015_159_MOESM7_ESM.docx]

**Additional File 7 Summary of exposure in each treatment arm (Safety population)**

|  | Exposure to treatment | | | | | | | | |
| --- | --- | --- | --- | --- | --- | --- | --- | --- | --- |
|  |  | Placebo | FF/VI  100/25 μg OD | FF/VI 200/25 μg OD | FF 100 μg OD | FF 200 μg OD | FP 100 μg BD | FP 250 μg BD | FP 500 μg BD |
| Overall | N | 404 | 1,210 | 197 | 1,430 | 390 | 102 | 100 | 195 |
|  | Total patient exposure (years) | 65.1 | 1,065 | 84.3 | 1,081 | 104 | 14.11 | 14.13 | 80.06 |
|  | Exposure (days) | | | | | | | | |
|  | n | 402 | 1,210 | 196 | 1,429 | 387 | 101 | 100 | 195 |
|  | Mean (SD) | 59.1  (25.0) | 321  (137) | 157  (34.8) | 276  (160) | 98.1 (58.7) | 51.0 (13.6) | 51.6 (13.1) | 150  (46.7) |
|  | Median | 57.0 | 361 | 169 | 353 | 58.0 | 57.0 | 57.0 | 169 |
|  | Range: min, max | 3, 98 | 1, 543 | 9, 189 | 1, 539 | 3, 189 | 8, 60 | 3, 59 | 3, 200 |
| Japan+Korea | N | 29 | 47* | 14* | 55 | 22 | 3^†^ | 7^†^ | 11^†^ |
|  | Total patient exposure (years) | 4.05 | 35.1* | 5.71* | 31.2 | 5.56 | 0.45^†^ | 1.07^†^ | 4.65^†^ |
|  | Exposure (days) | | | | | | | | |
|  | n | 29 | 47* | 14* | 55 | 22 | 3^†^ | 7 ^†^ | 11^†^ |
|  | Mean (SD) | 51.0  (28.8) | 273*  (164) | 149*  (48.0) | 207  (168) | 92.3 (56.8) | 55.3^†^  (1.53) | 55.6^†^  (1.51) | 155^†^  (47.0) |
|  | Median | 56.0 | 316* | 168* | 85.0 | 57.0 | 55.0^†^ | 55.0^†^ | 169^†^ |
|  | Range: min, max | 7, 87 | 10, 490* | 27, 170* | 14, 525 | 29, 171 | 54, 57^†^ | 53, 57^†^ | 13, 171^†^ |
| Not-Japan+Korea | N | 375 | 1,163 | 183 | 1,375 | 368 | 99 | 93 | 184 |
|  | Total patient exposure (years) | 61.1 | 1,029 | 78.6 | 1,050 | 98.3 | 13.7 | 13.1 | 75.4 |
|  | Exposure (days) | | | | | | | | |
|  | n | 373 | 1,163 | 182 | 1,374 | 365 | 98 | 93 | 184 |
|  | Mean (SD) | 59.8  (24.6) | 323  (135) | 158  (33.7) | 279  (159) | 98.4 (58.9) | 50.9 (13.8) | 51.3 (13.5) | 150 (46.8) |
|  | Median | 57.0 | 361 | 169 | 354 | 58.0 | 57.0 | 57.0 | 169 |
|  | Range: min, max | 3, 98 | 1, 543 | 9, 189 | 1, 539 | 3, 189 | 8, 60 | 3, 59 | 3, 200 |

BD, twice daily; FF, fluticasone furoate; FP, fluticasone propionate; min, minimum; max, maximum OD, once daily; SD, standard deviation; VI, vilanterol.

Safety population includes FFA109685, FFA109687, HZA106827, HZA106829, and HZA106837. *Includes patients recruited from Japan only; ^†^Includes patients recruited from Korea only.
