# Supplementary material for: Ethnic sensitivity assessment of fluticasone furoate/vilanterol in East Asian asthma patients from randomized double-blind multicentre Phase IIb/III trials
Source: BMC Pulm Med. 2015 Dec 24;15:165. doi: 10.1186/s12890-015-0159-z (PMC4690330; doi:10.1186/s12890-015-0159-z)
Supplement: Additional file 8: — AEs reported by ≥3 patients in the Japan+Korea cohort of any relevant treatment arm and the corresponding incidence in the Not-Japan+Korea cohort and the Overall population (Safety population). (DOCX 26.9 KB) [file 12890_2015_159_MOESM8_ESM.docx]

Additional File 8 AEs reported by ≥3 patients in the Japan+Korea cohort of any relevant treatment arm and the corresponding incidence in the Not-Japan+Korea cohort and the Overall population (Safety population)

| Preferred term | Number of patients reporting an AE, n (%) | | | | |
| --- | --- | --- | --- | --- | --- |
|  | Placebo | FF/VI  100/25 μg OD | FF/VI  200/25 μg OD | FF  100 μg OD | FF  200 μg OD |
| Range of treatment duration (weeks) of studies integrated | 8–12 | 12–76 | 24 | 8–76 | 8–24 |
| Japan+Korea, n | 29 | 47 | 14 | 55 | 22 |
| Overall, N | 404 | 1210 | 197 | 1430 | 390 |
| Nasopharyngitis | | | | | |
| Japan+Korea | 6 (21) | 23 (49)* | 7 (50)* | 16 (29) | 5 (23) |
| Not-Japan+Korea | 18 (5) | 152 (13) | 18 (10) | 142 (10) | 30 (8) |
| Overall | 24 (6) | 175 (14) | 25 (13) | 158 (11) | 35 (9) |
| Headache | | | | | |
| Japan+Korea | 1 (3) | 3 (6)* | 0* | 4 (7) | 2 (9) |
| Not-Japan+Korea | 23 (6) | 195 (17) | 11 (6) | 205 (15) | 24 (7) |
| Overall | 24 (6) | 198 (16) | 11 (6) | 209 (15) | 26 (7) |
| Bronchitis | | | | | |
| Japan+Korea | 0 | 4 (9)* | 0* | 3 (5) | 1 (5) |
| Not-Japan+Korea | 4 (1) | 56 (5) | 7 (4) | 73 (5) | 6 (2) |
| Overall | 4 (<1) | 60 (5) | 7 (4) | 76 (5) | 7 (2) |
| Upper respiratory tract infection | | | | | |
| Japan+Korea | 0 | 3 (6)* | 0* | 4 (7) | 0 |
| Not-Japan+Korea | 3 (<1) | 73 (6) | 2 (1) | 98 (7) | 6 (2) |
| Overall | 3 (<1) | 76 (6) | 2 (1) | 102 (7) | 6 (2) |
| Abdominal pain upper | | | | | |
| Japan+Korea | 0 | 1 (2)* | 0* | 3 (5) | 0 |
| Not-Japan+Korea | 1 (<1) | 35 (3) | 1 (<1) | 24 (2) | 1 (<1) |
| Overall | 1 (<1) | 36 (3) | 1 (<1) | 27 (2) | 1 (<1) |
| Dermatitis | | | | | |
| Japan+Korea | 0 | 4 (9)* | 0* | 1 (2) | 0 |
| Not-Japan+Korea | 0 | 2 (<1) | 0 | 3 (<1) | 0 |
| Overall | 0 | 6 (<1) | 0 | 4 (<1) | 0 |
| Dizziness | | | | | |
| Japan+Korea | 1 (3) | 4 (9)* | 0* | 1 (2) | 1 (5) |
| Not-Japan+Korea | 2 (<1) | 10 (<1) | 0 | 10 (<1) | 1 (<1) |
| Overall | 3 (<1) | 14 (1) | 0 | 11 (<1) | 2 (<1) |
| Oropharyngeal pain | | | | | |
| Japan+Korea | 0 | 3 (6)* | 0* | 2 (4) | 0 |
| Not-Japan+Korea | 4 (1) | 42 (4) | 4 (2) | 63 (5) | 12 (3) |
| Overall | 4 (<1) | 45 (4) | 4 (2) | 65 (5) | 12 (3) |

AE, adverse event; FF, fluticasone furoate; OD, once daily; VI, vilanterol.

Safety population consists of data from studies HZA106827, HZA106829, HZA106837, FFA109685, and FFA109687. *Only patients recruited from Japan.
