# Supplementary material for: Ethnic sensitivity assessment of fluticasone furoate/vilanterol in East Asian asthma patients from randomized double-blind multicentre Phase IIb/III trials
Source: BMC Pulm Med. 2015 Dec 24;15:165. doi: 10.1186/s12890-015-0159-z (PMC4690330; doi:10.1186/s12890-015-0159-z)
Supplement: Additional file 9: — Region ratios of ‘treatment effect’ in Japan+Korea and Not-Japan+Korea patients, where ‘treatment effect’ is the comparison between treatment arms of 24h urinary cortisol excretion ratio (end of treatment/baseline; Urinary Cortisol population). (DOCX 944 KB) [file 12890_2015_159_MOESM9_ESM.docx]

**Additional File 9 Region ratios of ‘treatment effect’ in Japan+Korea and Not-Japan+Korea patients, where ‘treatment effect’ is the comparison between treatment arms of 24h urinary cortisol excretion ratio (end of treatment/baseline; Urinary Cortisol population)**


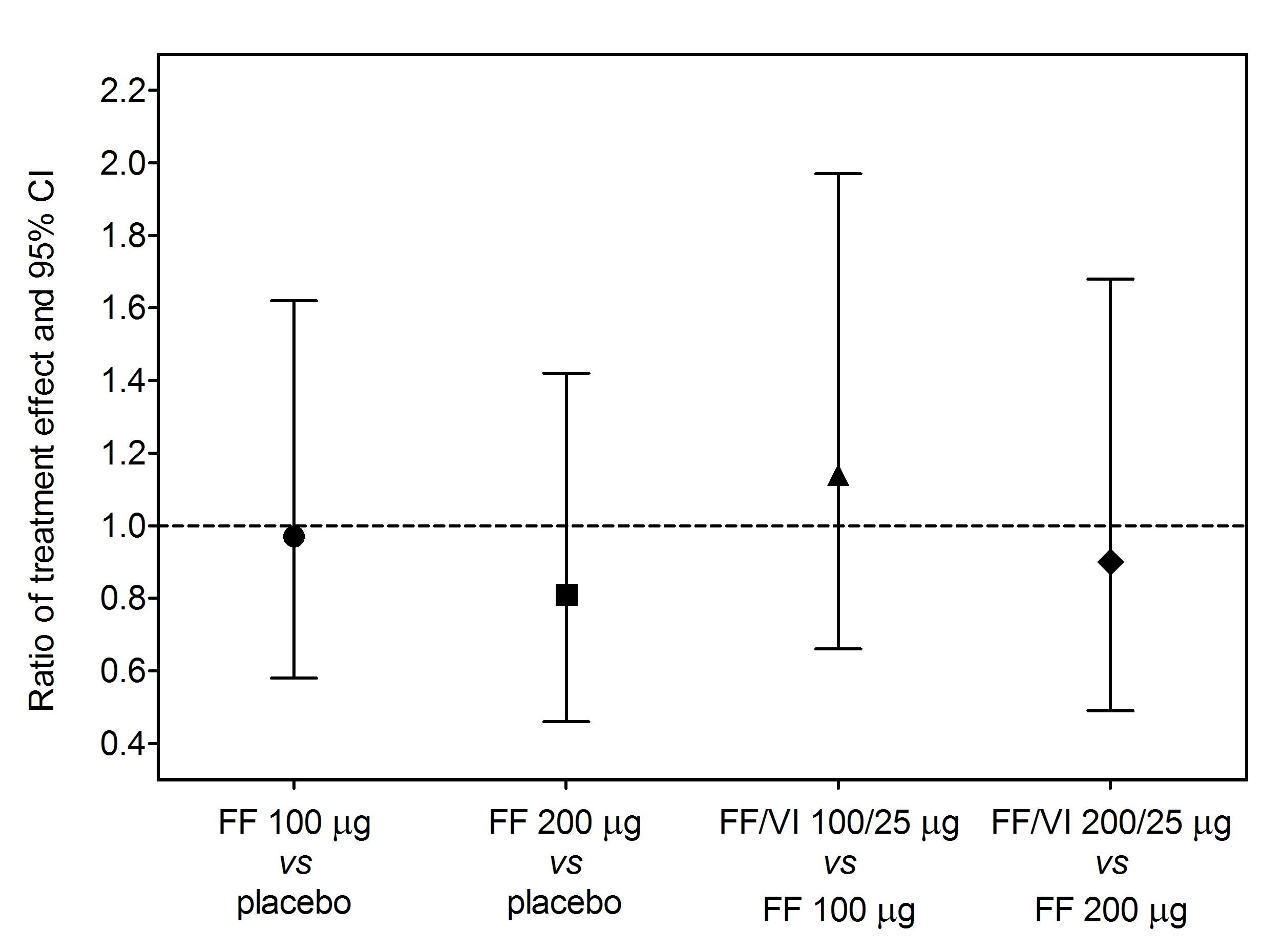


CI, confidence interval; FF, fluticasone furoate; VI, vilanterol.

Urinary cortisol population includes a subset of patients included in studies FFA109685, FFA109687, HZA106827, and HZA106829 for whom urine samples were available and which were not considered to have confounding factors that could have affected the interpretation of the results. Number of patients analyzed in a) Japan/Korea cohort: N = 14 placebo, N = 12 FF/VI 100/25 μg OD, N = 10 FF/VI 200/25 μg OD, N = 21 FF 100 μg OD, N = 14 FF 200 μg OD; b) Not-Japan/Korea cohort: N = 244 placebo, N = 141 FF/VI 100/25 μg OD, N = 130 FF/VI 200/25 μg OD, N = 280 FF 100 μg OD, N = 256 FF 200 μg OD.
